# Supplementary material for: Development of the individualised Comparative Effectiveness of Models Optimizing Patient Safety and Resident Education (iCOMPARE) trial: a protocol summary of a national cluster-randomised trial of resident duty hour policies in internal medicine
Source: BMJ Open. 2018 Sep 21;8(9):e021711. doi: 10.1136/bmjopen-2018-021711 (PMC6157525; doi:10.1136/bmjopen-2018-021711)

Development of the Individualized Comparative Effectiveness of Models Optimizing Patient Safety and Resident Education (iCOMPARE) Trial: A Protocol Summary of a National Cluster-Randomized Trial of Resident Duty Hour Policies in Internal Medicine

Judy A. Shea, PhD,[1] Jeffrey H. Silber, MD, PhD,[2] Sanjay V. Desai, MD,[3] David F. Dinges PhD,[4] Lisa M. Bellini, MD,[1] James Tonascia, PhD,[5] Alice L. Sternberg, ScM,[6] Dylan S. Small, PhD,[7] David M. Shade, JD,[6] Joel T. Katz, MD,[8] Mathias Basner, MD, PhD,[4] Krisda H. Chaiyachati, MD, MPH,[1,10] Orit Even-Shoshan, MS,[2] David W. Bates, MD, MSc,[8] Kevin G. Volpp, MD, PhD,[9,10] David A. Asch, MD, MBA[1,10] and the iCOMPARE Research Group

Supplementary Appendix

Table of Contents

Appendix Materials 1. Rationale and ICD-9 Codes for Principal Diagnoses Qualifying Hospital/Patient for Inclusion in iCOMPARE Randomization/Analysis

Appendix Materials 2. Details for Secondary Outcomes for Patient Safety Aim: Prolonged Length of Stay, Readmissions, Complications, and Costs

Appendix Materials 3. Sleep Actigraphy Scoring

Appendix Figure 1. Overview of Actigraphy and PVT Scoring and Review Process

Appendix Figure 2. Sleep Scoring Matrix

Appendix Materials 1. Rationale and ICD-9 Codes for Principal Diagnoses Qualifying Hospital/Patient for Inclusion in iCOMPARE Randomization/Analysis

The original ICD-9 code list was created by searching for relevant codes for each medical condition that were found to be associated with high mortality rates in our preliminary 2008 data. The outcomes team reviewed the code list and expanded the code list in two ways:

- We consulted the official ICD-9 code book and looked for additional high-volume codes that were related to the medical conditions of interest. The list of proposed expansion codes was reviewed and approved by the coding consultant to the study, Dr. Patrick Romano of the University of California, Davis.
- To account for the presence of ICD-10 codes on claims with discharge dates beginning October 1, 2015, we utilized the General Equivalence Mappings (GEMs) made available by the Centers for Medicare and Medicaid Services, which provide the closest possible approximation of a translation between the ICD-9 and ICD-10 code systems. Our goal was to translate each ICD-10 code back to its most closely equivalent ICD-9 code, and our review of the crosswalk caused us to pick up a small number of additional ICD-9 codes. As with the original code list expansion, these proposed additional codes were reviewed and approved by our coding consultant to the study.

Pneumonia:

481	Pneumococcal Pneumonia [Streptococcus Pneumoniae Pneumonia]
482	Other Bacterial Pneumonia
482.1	Pneumonia Due to Pseudomonas
482.2	Pneumonia Due to Hemophilus Influenzae (H. Influenzae)
482.3	Pneumonia Due to Streptococcus

482.31	Pneumonia Due to Streptococcus Group A
482.32	Pneumonia Due to Streptococcus Group B
482.39	Pneumonia Due to Other Streptococcus
482.41	Methicillin Susceptible Pneumonia Due to Staphylococcus Aureus
482.42	Methicillin Resistant Pneumonia Due to Staphylococcus Aureus
482.49	Other Staphylococcus Pneumonia
482.82	Pneumonia Due to Escherichia Coli [E.Coli]
482.83	Pneumonia Due to Other Gram-Negative Bacteria
482.84	Pneumonia Due to Legionnaires' Disease
482.89	Pneumonia Due to Other Specified Bacteria
482.4	Pneumonia Due to Staphylococcus
482.9	Bacterial Pneumonia Unspecified
483	Pneumonia Due to Other Specified Organism
483.1	Pneumonia Due to Chlamydia
483.8	Pneumonia Due to Other Specified Organism
485	Bronchopneumonia Organism Unspecified
486	Pneumonia Organism Unspecified

Stroke:

430	Subarachnoid Hemorrhage
431	Intracerebral Hemorrhage
432.0	Nontraumatic Extradural Hemorrhage
432.1	Subdural Hemorrhage
432.9	Unspecified Intracranial Hemorrhage

433.01 Occlusion and Stenosis of Basilar Artery with Cerebral Infarction

433.11 Occlusion and Stenosis of Carotid Artery with Cerebral Infarction

433.21 Occlusion and Stenosis of Vertebral Artery with Cerebral Infarction

433.31 Occlusion and Stenosis of Multiple and Bilateral Precerebral Arteries with Cerebral Infarction

433.81 Occlusion and Stenosis of Other Specified Precerebral Artery with Cerebral Infarction

433.91 Occlusion and Stenosis of Unspecified Precerebral Artery with Cerebral Infarction

434.01 Cerebral Thrombosis with Cerebral Infarction

434.11 Cerebral Embolism with Cerebral Infarction

434.91 Cerebral Artery Occlusion Unspecified with Cerebral Infarction

436 Acute but Ill-Defined Cerebrovascular Disease

Acute Myocardial Infarction:

410.01 Acute Myocardial Infarction of Anterolateral Wall Initial Episode of Care

410.11 Acute Myocardial Infarction of Other Anterior Wall Initial Episode of Care

410.21 Acute Myocardial Infarction of Inferolateral Wall Initial Episode of Care

410.31 Acute Myocardial Infarction of Inferoposterior Wall Initial Episode of Care

410.41 Acute Myocardial Infarction of Other Inferior Wall Initial Episode of Care

410.51 Acute Myocardial Infarction of Other Lateral Wall Initial Episode of Care

410.61 True Posterior Wall Infarction Initial Episode of Care

410.71 Subendocardial Infarction Initial Episode of Care

410.81 Acute Myocardial Infarction of Other Specified Sites Initial Episode of Care

410.91 Acute Myocardial Infarction of Unspecified Site Initial Episode of Care

Gastrointestinal Hemmorhage:

456.0 Esophageal Varices with Bleeding

530.7 Gastroesophageal Laceration-Hemorrhage Syndrome

530.82 Esophageal Hemorrhage

531.00 Acute Gastric Ulcer with Hemorrhage Without Obstruction

531.01 Acute Gastric Ulcer with Hemorrhage with Obstruction

531.20 Acute Gastric Ulcer with Hemorrhage and Perforation Without Obstruction

531.21 Acute Gastric Ulcer with Hemorrhage and Perforation with Obstruction

531.40 Chronic or Unspecified Gastric Ulcer with Hemorrhage Without Obstruction

531.41 Chronic or Unspecified Gastric Ulcer with Hemorrhage with Obstruction

531.60 Chronic or Unspecified Gastric Ulcer with Hemorrhage and Perforation Without Obstruction

531.61 Chronic or Unspecified Gastric Ulcer with Hemorrhage and Perforation with Obstruction

532.00 Acute Duodenal Ulcer with Hemorrhage Without Obstruction

532.01 Acute Duodenal Ulcer with Hemorrhage with Obstruction

532.20 Acute Duodenal Ulcer with Hemorrhage and Perforation Without Obstruction

532.21 Acute Duodenal Ulcer with Hemorrhage and Perforation with Obstruction

532.40 Chronic or Unspecified Duodenal Ulcer with Hemorrhage Without Obstruction

532.41 Chronic or Unspecified Duodenal Ulcer with Hemorrhage with Obstruction

532.60 Chronic or Unspecified Duodenal Ulcer with Hemorrhage and Perforation Without Obstruction

532.61 Chronic or Unspecified Duodenal Ulcer with Hemorrhage and Perforation with Obstruction

533.00 Acute Peptic Ulcer of Unspecified Site with Hemorrhage Without Obstruction

533.01 Acute Peptic Ulcer of Unspecified Site with Hemorrhage with Obstruction

533.20 Acute Peptic Ulcer of Unspecified Site with Hemorrhage and Perforation Without Obstruction

533.21 Acute Peptic Ulcer of Unspecified Site with Hemorrhage and Perforation with Obstruction

533.40 Chronic or Unspecified Peptic Ulcer of Unspecified Site with Hemorrhage Without Obstruction

533.41 Chronic or Unspecified Peptic Ulcer of Unspecified Site with Hemorrhage with Obstruction

533.60 Chronic or Unspecified Peptic Ulcer of Unspecified Site with Hemorrhage and Perforation Without Obstruction

533.61 Chronic or Unspecified Peptic Ulcer of Unspecified Site with Hemorrhage and Perforation with Obstruction

534.00 Acute Gastrojejunal Ulcer with Hemorrhage Without Obstruction

534.01 Acute Gastrojejunal Ulcer with Hemorrhage with Obstruction

534.20 Acute Gastrojejunal Ulcer with Hemorrhage and Perforation Without Obstruction

534.21 Acute Gastrojejunal Ulcer with Hemorrhage and Perforation with Obstruction

534.40 Chronic or Unspecified Gastrojejunal Ulcer with Hemorrhage Without Obstruction

534.41 Chronic or Unspecified Gastrojejunal Ulcer with Hemorrhage with Obstruction

534.60 Chronic or Unspecified Gastrojejunal Ulcer with Hemorrhage and Perforation Without Obstruction

534.61 Chronic or Unspecified Gastrojejunal Ulcer with Hemorrhage and Perforation with Obstruction

535.01 Acute Gastritis with Hemorrhage

535.11 Atrophic Gastritis with Hemorrhage

535.21 Gastric Mucosal Hypertrophy with Hemorrhage

535.31 Alcoholic Gastritis with Hemorrhage

535.41 Other Specified Gastritis with Hemorrhage

535.51 Unspecified Gastritis and Gastroduodenitis with Hemorrhage

535.61 Duodenitis with Hemorrhage

537.83 Angiodysplasia of Stomach and Duodenum with Hemorrhage

562.02 Diverticulosis of Small Intestine with Hemorrhage

562.03 Diverticulitis of Small Intestine with Hemorrhage

562.12 Diverticulosis of Colon with Hemorrhage

562.13 Diverticulitis of Colon with Hemorrhage

569.3 Hemorrhage of Rectum and Anus

569.85 Angiodysplasia of Intestine with Hemorrhage

578.0 Hematemesis

578.1 Blood In Stool

578.9 Hemorrhage of Gastrointestinal Tract Unspecified

Heart Failure:

398.91 Rheumatic Heart Failure (Congestive)

402.01 Malignant Hypertensive Heart Disease with Heart Failure

402.11 Benign Hypertensive Heart Disease with Heart Failure

402.91 Unspecified Hypertensive Heart Disease with Heart Failure

404.01 Hypertensive Heart and Chronic Kidney Disease Malignant with Heart Failure with Chronic Kidney Disease Stage I Through Stage Iv or Unspecified

404.03 Hypertensive Heart and Chronic Kidney Disease Malignant with Heart Failure with Chronic Kidney Disease Stage V or End Stage Renal Disease

404.11 Hypertensive Heart and Chronic Kidney Disease Benign with Heart Failure with Chronic Kidney Disease Stage I Through Stage Iv or Unspecified

404.13 Hypertensive Heart and Chronic Kidney Disease Benign with Heart Failure with Chronic Kidney Disease Stage V or End Stage Renal Disease

404.91 Hypertensive Heart and Chronic Kidney Disease Unspecified with Heart Failure with Chronic Kidney Disease Stage I Through Stage Iv or Unspecified

404.93 Hypertensive Heart and Chronic Kidney Disease Unspecified with Heart Failure with Chronic Kidney Disease Stage V or End Stage Renal Disease

428 Heart Failure

428.0	Congestive Heart Failure Unspecified
428.1	Left Heart Failure
428.20	Unspecified Systolic Heart Failure
428.21	Acute Systolic Heart Failure
428.22	Chronic Systolic Heart Failure
428.23	Acute on Chronic Systolic Heart Failure
428.30	Unspecified Diastolic Heart Failure
428.31	Acute Diastolic Heart Failure
428.32	Chronic Diastolic Heart Failure
428.33	Acute on Chronic Diastolic Heart Failure
428.40	Unspecified Combined Systolic and Diastolic Heart Failure
428.41	Acute Combined Systolic and Diastolic Heart Failure
428.42	Chronic Combined Systolic and Diastolic Heart Failure
428.43	Acute on Chronic Combined Systolic and Diastolic Heart Failure
428.9	Heart Failure Unspecified

Septicemia:

038	Septicemia
038.0	Streptococcal Septicemia
038.1	Staphylococcal Septicemia
038.9	Unspecified Septicemia

Kidney Failure:

584	Acute Kidney Failure

584.9	Acute Kidney Failure Unspecified

Cardiac Disease:

427.31	Atrial Fibrillation
427.41	Ventricular Fibrillation
427.5	Cardiac Arrest

Chronic Obstructive Pulmonary Disease:

490	Bronchitis Not Specified as Acute or Chronic
491.21	Obstructive Chronic Bronchitis with (Acute) Exacerbation
491.22	Obstructive Chronic Bronchitis with Acute Bronchitis

Pancreatitis:

577.0	Acute Pancreatitis
577.9	Unspecified Disease of Pancreas

Acute Respiratory Failure:

518.81	Acute Respiratory Failure
518.84	Acute and Chronic Respiratory Failure
518.89	Other Diseases of Lung Not Elsewhere Classified
519.11	Acute Bronchospasm

Chest Pain:

786.5	Chest Pain

786.59	Other Chest Pain

Cellulitis:

682	Other Cellulitis and Abscess
682.6	Cellulitis and Abscess of Leg Except Foot

Coronary Atherosclerosis:

414.01	Coronary Atherosclerosis of Native Coronary Artery
414.1	Aneurysm and Dissection of Heart

Pulmonary Embolism:

415	Acute Pulmonary Heart Disease
415.12	Septic Pulmonary Embolism
415.19	Other Pulmonary Embolism and Infarction

Syncope:

780.0	Alteration of Consciousness
780.2	Syncope and Collapse
780.3	Convulsions
780.01	Coma

Intestinal Infection:

008	Intestinal Infections Due to Other Organisms
008.45	Intestinal Infection Due to Clostridium Difficile

008.49 Intestinal Infection Due to Other Organisms

Obstructive Asthma:

493.9 Asthma Unspecified

493.22 Chronic Obstructive Asthma with (Acute) Exacerbation

Bronchitis:

494.1 Bronchiectasis with Acute Exacerbation

Appendix Materials 2: Details for Secondary Outcomes for Patient Safety Aim: Prolonged Length of Stay, Readmissions, Complications, and Costs

All patient safety measures are based on information made available to researchers by the Centers for Medicare and Medicaid Services (CMS). Health services researchers have conducted research using Medicare claims files for over two decades. This information is uniformly recorded for all Medicare beneficiaries. CMS releases to researchers the de-identified claim files for hospitals' and physicians' services after validating them through an adjudicated process. All the patient safety outcomes are calculated by the outcomes team based on information from the claims data.

The reliability of the data used for calculating patient safety outcomes for mortality, readmissions, and length of stay (LOS) is excellent. The reliability of the data used to calculate complications, costs, and payments is also high, but lower than of the data used in calculating the 30- day mortality, readmissions and LOS outcomes, due to variation across hospitals in the number and may vary across hospitals due in the number of diagnostic and procedure codes recorded in the claim. However, due to the randomized nature of the study, we would expect there to be no difference in these outcomes between the two study arms (FLEX and STD). References regarding the reliability of the various patient safety measures are provided below.

In addition to mortality, the following outcomes measures were collected:

- Readmission: This calculation is based on the admission and discharge dates in the Medicare claims.
- Length of Stay and Prolonged Length of Stay: Calculated based on the admission and discharge dates of the index claim.
- Complications: Calculated using the Agency for Healthcare Research and Quality Patient Safety Indicators (see below).

- Costs: Calculated using inpatient, revenue center, and Part B claims. We utilize a resource costing method (more details are provided below) to estimate the costs associated with accommodations (general floor and intensive care unit), the operating room, post-discharge emergency room visits, and other services indicated by the presence of Current Procedural Terminology codes (which are translated to Relative Value Units).
- Payments: Calculated using the payment variables that appear in the inpatient, outpatient, and Part B claims. The total amounts paid by Medicare, the beneficiary, and the primary payer are summed. Year-based adjustments for inflation are applied to the payment figures.

The Patient Safety Indicators were calculated by the research team using SAS programs provided by the Agency for Healthcare Research and Quality, which were run on the Medicare claims. Some of the Patient Safety Indicators that were considered "postoperative" or "perioperative" were modified for use with the study's population of medical patients. For these Patient Safety Indicators, the portion of the code that required the patient to have had surgery was deleted.

The following Patient Safety Indicators were used:

- PSI 03 - Pressure ulcer rate
- PSI 06 - Iatrogenic pneumothorax rate
- PSI 07 - Central venous catheter-related blood stream infection rate
- PSI 08 - Postoperative hip fracture rate
- PSI 09 - Perioperative hemorrhage or hematoma rate
- PSI 10 - Postoperative physiologic and metabolic derangement rate
- PSI 11 - Postoperative respiratory failure rate
- PSI 12 - Perioperative pulmonary embolism or deep vein thrombosis rate
- PSI 13 - Postoperative sepsis rate

References describing the validity of the claims data used to calculate the patient safety outcomes are in the main manuscript and repeated here:

Readmissions:

- Patel MS, Volpp KG, Small DS, et al. Association of the 2011 ACGME resident duty hour reforms with mortality and readmissions among hospitalized Medicare patients. JAMA 2014;312:2364-73.
- Krumholz HM, Lin Z, Keenan PS, et al. Relationship between hospital readmission and mortality rates for patients hospitalized with acute myocardial infarction, heart failure, or pneumonia. JAMA 2013;309:587-93.

Length of Stay and Prolonged Length of Stay:

- Silber JH, Rosenbaum PR, Even-Shoshan O, et al. Length of stay, conditional length of stay, and prolonged stay in pediatric asthma. Health Serv Res 2003;38:867-86.
- Silber JH, Rosenbaum PR, Kelz RR, et al. Medical and financial risks associated with surgery in the elderly obese. Ann Surg 2012;256:79-86.
- Silber JH, Rosenbaum PR, Koziol LF, et al. Conditional length of stay. Health Serv Res 1999;34:349-63.
- Silber JH, Rosenbaum PR, Rosen AK, et al. Prolonged hospital stay and the resident duty hour rules of 2003. Med Care 2009;47:1191-200.

Patient Safety Indicators (description of PSIs):

- Rosen AK, Loveland SA, Romano PS, et al. Effects of resident duty hour reform on surgical and procedural patient safety indicators among hospitalized Veterans Health Administration and Medicare patients. Med Care 2009;47:723-31.

Patient Safety Indicators (validity):

- Romano PS, Geppert JJ, Davies S, et al. A national profile of patient safety in US hospitals. Health Aff. 2003;22:154–166.
- Rosen AK, Rivard P, Zhao S, et al. Evaluating the patient safety indicators: how well do they perform on Veterans Health Administration data? Med Care. 2005;43:873–884.
- Zhan C, Miller MR. Excess length of stay, charges, and mortality attributable to medical injuries during hospitalization. JAMA. 2003;290:1868–1874.

Costs are calculated using a resource utilization-based method of cost estimation. The following items, which are calculated using the Medicare claims data, are included in the total cost estimate:

- Accommodation costs, which are based on the number of general floor days and the number of intensive care unit days during the index admission. This information comes from the revenue center files.
- Operating room cost, based on the amount of time spent in the operating room (for patients who had a surgical procedure performed). This is determined using Part B claims.
- Emergency room visit fixed costs, based on post-discharge visits to the emergency room. This is determined using Part B claims.
- Costs of services provided, based on Relative Value Units (RVUs), determined using the Current Procedural Terminology codes on bills. This is determined using Part B claims.

In addition, any costs that occurred within 30 days of the index admission date, and all the costs associated with any readmissions that began within 30 days, are also included in the total cost calculation.

Drugs administered during the hospitalization are included in the cost calculation, but drugs outside the hospital are not. Costs of lawsuits are not available in the Medicare claims data.

Hospital medical errors that resulted in a longer length of stay or the use of additional hospital resources, such as the need to admit a patient to the intensive care unit, would be captured in the cost calculation.

Appendix Materials 3: Sleep Actigraphy Scoring

According to conventional standards, actigraphy data were classified in 1-minute epochs as wake, sleep, or missing. The first classification was performed by the algorithm of the device manufacturer (Actilife software, version 6.13.3, standard settings, Sadeh scoring algorithm). Off-wrist periods were identified by an algorithm developed by Pulsar Informatics Inc. (Philadelphia, PA), then visually checked, integrated with sleep log information provided by interns via the smartphone and, if necessary, corrected by co-investigators from Pulsar who were experienced with actigraphy scoring of large scale clinical trials. The classification into sleep, wake, and unknown followed the set of rules outlined in Appendix Figure 1. Pulsar's visual scoring of sleep was then independently checked by study investigators who are experts in sleep research. In an iterative process, any discrepancies were documented and then corrected by Pulsar, until agreement with the study investigators was reached. During this visual scoring process, both Pulsar and the study investigators were blinded to study arm (STD or FLEX). Likewise, PVT-B data were inspected by study sleep experts blinded to arm (Appendix Figure 2). PVT-B performance was classified into three categories as [1] adherent (i.e., PVT-B data reflected an effort to do the task correctly, and comments left by the subject did not suggest non-adherence), [2] possibly non-adherent (i.e., PVT-B data reflected a consistently poor effort to do the task correctly, but comments left by the subject did not suggest non-adherence), and [3] non-adherent (i.e., PVT-B data reflected a consistently poor effort to do the task correctly, and comments left by the subject did suggest non-adherence, e.g., performing the task while brushing teeth). Comments left by interns were inspected for distractions and non-fatigue related impairment and flagged accordingly. Comments that could have revealed the study arm were blacked out by Pulsar prior to classification by the study sleep experts.

Appendix Figure 1. Overview of Actigraphy and PVT Scoring and Review Process

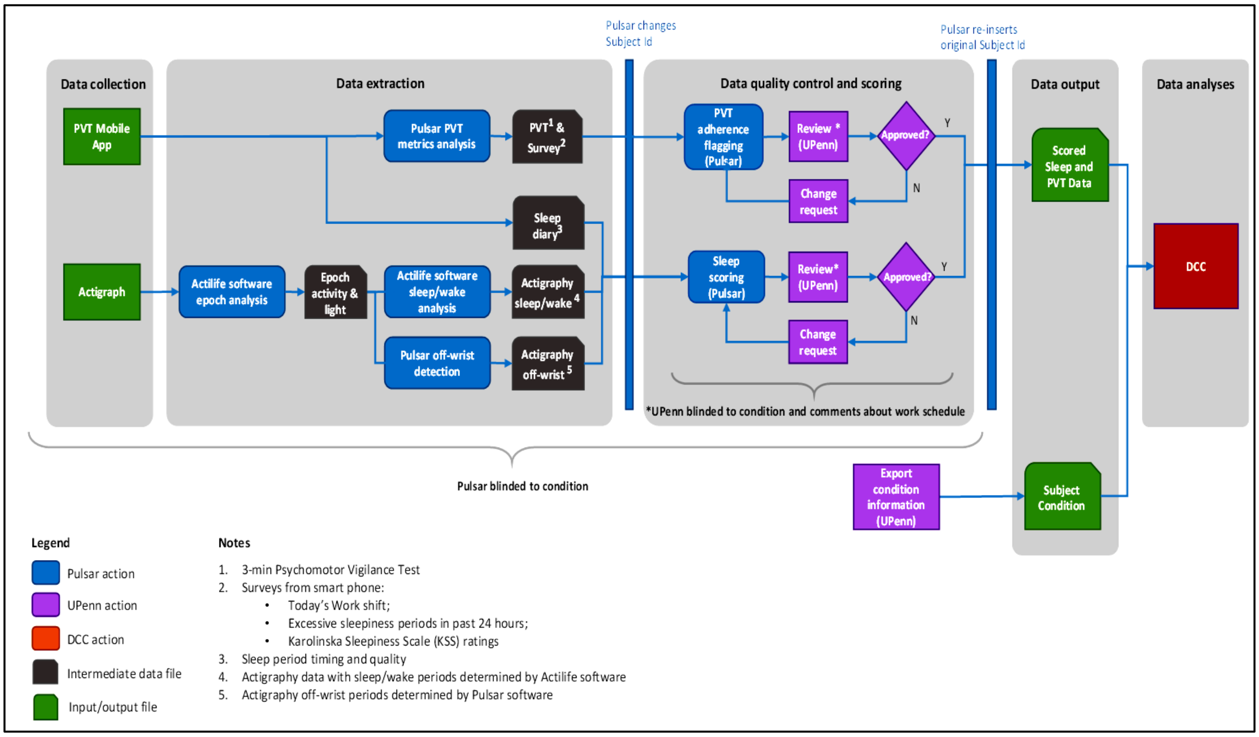

Appendix Figure 2: Sleep Scoring Matrix

	**Sleep Diary had sleep time entered (S)**	**Sleep Diary did not have sleep time entered (W)**
**Actigraphy Reviewed indicated sleep (S)**	Scored as Sleep (S) based on agreement of actigraphy and diary	Scored as Sleep (S) based on actigraphy
**Actigraphy Reviewed indicated wake (W)**	Scored as Wake (W) based on actigraphy	Scored as Wake (W) based on actigraphy
**Actigraphy Reviewed indicated missing or off-wrist (O)**	Scored as Sleep (S) based on diary	Scored as Unknown (U)

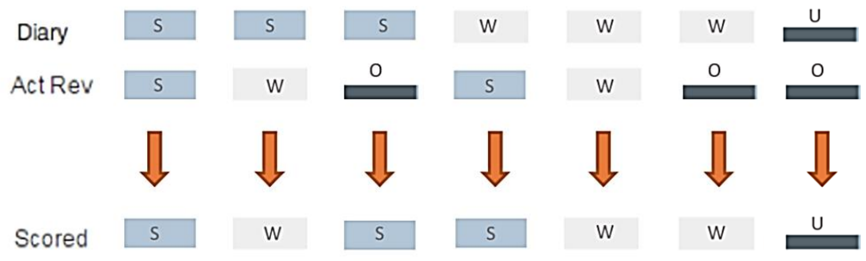

Supplement: Supplementary file 1 [file bmjopen-2018-021711supp001.pdf]
