# Supplementary material for: Development of the individualised Comparative Effectiveness of Models Optimizing Patient Safety and Resident Education (iCOMPARE) trial: a protocol summary of a national cluster-randomised trial of resident duty hour policies in internal medicine
Source: BMJ Open. 2018 Sep 21;8(9):e021711. doi: 10.1136/bmjopen-2018-021711 (PMC6157525; doi:10.1136/bmjopen-2018-021711)
Supplement: Author's manuscript [file bmjopen-2018-021711.draft_revisions.pdf]

BMJ Open

BMJ Open is committed to open peer review. As part of this commitment we make the peer review history of every article we publish publicly available.

When an article is published we post the peer reviewers' comments and the authors' responses online. We also post the versions of the paper that were used during peer review. These are the versions that the peer review comments apply to.

The versions of the paper that follow are the versions that were submitted during the peer review process. They are not the versions of record or the final published versions. They should not be cited or distributed as the published version of this manuscript.

BMJ Open is an open access journal and the full, final, typeset and author-corrected version of record of the manuscript is available on our site with no access controls, subscription charges or pay-per-view fees (http://bmjopen.bmj.com).

If you have any questions on BMJ Open's open peer review process please email editorial.bmjopen@bmj.com

BMJ Open

Development of the Individualized Comparative Effectiveness of Models Optimizing Patient Safety and Resident Education (iCOMPARE) Trial: A Protocol Summary of a National Cluster-Randomized Trial of Resident Duty Hour Policies in Internal Medicine

Journal:	*BMJ Open*
Manuscript ID	bmjopen-2018-021711
Article Type:	Protocol
Date Submitted by the Author:	16-Jan-2018

Complete List of Authors:	Shea, Judy; University of Pennsylvania Perelman School of Medicine, Medicine<br>Silber, JH; The Children's Hospital of Philadelphia, Pediatrics<br>Desai, Sanjay; The Johns Hopkins University, Medicine<br>Dinges, David ; University of Pennsylvania Perelman School of Medicine, Psychiatry<br>Bellini, Lisa; University of Pennsylvania Perelman School of Medicine, Medicine<br>Tonascia, James; John Hopkins , Biostatistics<br>Sternberg, Alice; Johns Hopkins Hospital and Health System, Epidemiology<br>Small, Dylan; University of Pennsylvania, Wharton Statistics<br>Shade, David ; Johns Hopkins University Bloomberg School of Public Health, Epidemiology<br>Katz, Joel; Brigham and Women's Hospital , Medicine<br>Basner, Mathias; University of Pennsylvania Perelman School of Medicine, Psychiatry<br>Chaiyachati, Krisda; University of Pennsylvania Perelman School of Medicine, Medicine ; Cpl Michael J Crescenz VA Medical Center<br>Even-Shoshan, Orit; Children's Hospital of Philadelphia , Pediatrics<br>Bates, David ; Brigham and Women's Hospital, Medicine<br>Volpp, Kevin ; University of Pennsylvania Perelman School of Medicine, Medical Ethics and Policy; Cpl Michael J Crescenz VA Medical Center<br>Asch, David; University of Pennsylvania Perelman School of Medicine, Medicine ; Cpl Michael J Crescenz VA Medical Center

Keywords:	resident education, duty hours, resident work hours

SCHOLARONE™ Manuscripts

Development of the Individualized Comparative Effectiveness of Models Optimizing Patient Safety and Resident Education (iCOMPARE) Trial: A Protocol Summary of a National Cluster-Randomized Trial of Resident Duty Hour Policies in Internal Medicine

Judy A. Shea, PhD,[1] Jeffrey H. Silber, MD, PhD,[2] Sanjay V. Desai, MD,[3] David F. Dinges PhD,[4] Lisa M. Bellini, MD,[1] James Tonascia, PhD,[5] Alice L. Sternberg, ScM,[6] Dylan S. Small, PhD,[7] David M. Shade, JD,[6] Joel T. Katz, MD,[8] Mathias Basner, MD, PhD,[4] Krisda H. Chaiyachati, MD, MPH,[1,10] Orit Even-Shoshan, MS,[2] David W. Bates, MD, MSc,[8] Kevin G. Volpp, MD, PhD,[9,10] David A. Asch, MD, MBA[1,10] and the iCOMPARE Research Group

[1]Department of Medicine, University of Pennsylvania, Philadelphia PA, USA

[2]Department of Pediatrics, Children's Hospital of Philadelphia, Philadelphia PA, USA

[3]Department of Medicine, the Johns Hopkins University, Baltimore MD, USA

[4]Department of Psychiatry, University of Pennsylvania, Philadelphia PA, USA

[5]Department of Biostatistics, the Johns Hopkins University, Baltimore MD, USA

[6]Department of Epidemiology, the Johns Hopkins University, Baltimore MD, USA

[7]Wharton Statistics Department, University of Pennsylvania, Philadelphia PA, USA

[8]Department of Medicine, Brigham and Women's Hospital, Boston, MA, USA

[9]Department of Medical Ethics and Policy, University of Pennsylvania, Philadelphia PA, USA

[10]the Cpl Michael J Crescenz VA Medical Center, Philadelphia PA

For peer review only - http://bmjopen.bmj.com/site/about/guidelines.xhtml

Acknowledgements: The iCOMPARE team extends their appreciation to the support provided by the National Heart, Lung, and Blood Institute (NHLBI), project number 5U01HL125388, the participating Program Directors, Jeremy M Asch, BA, Amanda K Bertram, MS, Michele M Carlin BA, Sara C Coats, BS, Adrian J. Ecker, Kelsey A. Gangemi, MPH, Alexander S. Hill, BS, Susan K. Malone, PhD, RN, Alyssa M. Yeager, MD.

Corresponding author:
Judy A. Shea, Ph.D.
1317 Blockley Hall
423 Guardian Drive
Philadelphia, PA 19104-6021
215 573-5111
215 573-8778 (fax)
sheaja@pennmedicine.upenn.edu

For peer review only

This paper is subject to NIH Public Access Policy

Trial Registration: clinicaltrials.org Identifier: NCT02274818

Running title: iCOMPARE Trial of Resident Duty Hour Policies

Number of references: 46

Number of tables: 3

Number of figures: 2

Number of supplementary appendices: 2

Word Count: 4359

Word count abstract: 253

Key words: resident education, duty hours, resident work hours,

For peer review only - http://bmjopen.bmj.com/site/about/guidelines.xhtml

Abstract

Introduction: Resident duty hours have been implemented without evidence simultaneously reflecting competing concerns about patient safety and physician education. The objective is to prospectively evaluate the implications of alternative resident duty hour rules for patient safety, trainee education, and intern sleep and alertness.

Methods and Analysis. This is a cluster randomized trial of 63 United States internal medicine training programs from 2015-16. Programs were randomly assigned 1:1 to the 2011 Accreditation Council for Graduate Medical Education (ACGME) resident duty hour rules (STD) or to rules more flexible in intern shift length, and number of hours off between shifts (FLEX). The primary outcome is the difference in difference between pre and post intervention year 30-day any-site mortality between STD and FLEX programs among fee-for-service Medicare beneficiaries with any of several pre-specified principal diagnoses. Additional safety outcomes include readmission rates, complication rates, prolonged length of stay, and Medicare costs. Measures derived from trainees and program faculty from existing and study-specific surveys and from time motion studies of interns compare the educational experiences of residents. Measures derived from wrist actigraphy, sleep diaries, subjective ratings and psychomotor vigilance testing of interns compare the sleep and alertness of residents. Outcomes will be assessed by intention-to-treat analyses.

Ethics and dissemination. The University of Pennsylvania Institutional Review Board (IRB) approved the iCOMPARE protocol and served as the IRB of record for all participating programs that agreed to sign an Institutional Affiliation Agreement. Forty programs chose this option and submitted the required paperwork. Twenty-three programs opted for a local review process.

For peer review only - http://bmjopen.bmj.com/site/about/guidelines.xhtml

Trial Registration: clinicaltrials.org Identifier: NCT02274818

Article Summary:

Strengths and limitations of this study:

iCOMPARE is the largest randomized trial examining the impact of duty hours in internal medicine training program.

The aims are broad are include patient safety, trainees sleep and alertness and a host of education outcomes.

The protocol draws on a numbers of data collection methods including real time direct observation of trainee activities, multiple surveys, robust recording of sleep periods over a 14 day period and patient medical record data.

The lag between the observation period and data release for patient safety data in extensive.

iCOMPARE promises narrow but substantive evidence to inform resident duty hour standards in internal medicine and signals new interest in methodologically stronger research in medical education.

For peer review only - http://bmjopen.bmj.com/site/about/guidelines.xhtml

Introduction

The long hours worked by resident physicians received some academic attention in the 1970s,[1] but it was not until 1984 when those long hours became publicly linked with concerns about patient safety.[2] Those concerns resonated with a public who found it self-evident that the often 30-hour shifts of resident physicians would lead to fatigue, and that fatigue would lead to errors that would harm patients. Regulation of duty hours across all specialties occurred first in New York State as a reaction to the Libby Zion case, and then nationally in 2003 when the Accreditation Council for Graduate Medical Education (ACGME)—the organization that oversees resident education in the United States—limited residents to 80 hours of work per week averaged over 4 weeks, and limited the length of individual shifts to 24 hours, with an additional 4 hours to safely transfer care to the next resident. Partly on the basis of an Institute of Medicine report and a trial from Brigham and Women's Hospital,[3,4] those regulations were further tightened for first year residents (interns) in 2011, limiting their maximum shift length to 16 hours. This change prompted a charged debate.[5] Proponents argued that the restrictions did not go far enough. Others argued that the regulations were overly restrictive and inflexible, and that they harbored increased risk to patients by increasing patient handoffs.[6,7]

Meanwhile, large observational studies, using data from Medicare and the Veterans Administration across millions of hospitalizations, found essentially no difference in important patient outcomes following implementation of either the 2003 [8-16] or 2011 duty hour standards.[17] Several studies associated the 2011 standards with less direct patient contact, increased perception of medical errors, increased transitions of care, decreased educational opportunities, and only modestly increased quantities of sleep.[18-20] Others found no changes in trainees' educational test scores.[21] Program directors and trainees expressed concern that the rules reduced training quality and increased rather than decreased medical errors.[22-24] The increasing recognition of the importance of supervision –

For peer review only

with separate mandates implemented by the ACGME over the same period – added further uncertainty to the debate. In the end, a well-meaning effort to manage resident fatigue was perceived by many to promote burnout, increase handoffs, decrease educational opportunities and delay the professional maturation required to produce competent, independent physicians. Currently, the evidence available to resolve these controversies is limited to a patchwork of laboratory and in vivo studies of sleep deprivation, large scale epidemiological observations of patient outcomes, surveys of resident and educator opinions, and often single-center trials of unique residency duty hour designs that focused on workload or sleep but not patient outcomes.

In this context, we created the iCOMPARE (Individualized Comparative Effectiveness of Models Optimizing Patient Safety and Resident Education) trial, a cluster randomized trial carried out by internal medicine (IM) residency programs in the United States. For the 2015-2016 academic year, participating residency programs were randomized to one of two groups: 1) maintain standard (STD) duty hour rules, or 2) permit a more flexible (FLEX) set of duty hour rules, noted principally for removing the 16-hour shift length restriction for interns and allowing them to work up to 24 hours with an additional 4 hours for care transitions. In contrast to prior work, iCOMPARE was designed to simultaneously assess the impact of duty hour rules on patient safety, resident education, and intern sleep and alertness.

While the iCOMPARE trial was being planned, leaders in surgical education developed a roughly parallel trial, the Flexibility in Duty Hours Requirements for Surgical Trainees (FIRST) trial, which was fielded in the 2014-2015 academic year. The design of the FIRST trial and its initial results have been published.[25,26] Here we describe the design of the iCOMPARE trial.

Funding and Organization

The iCOMPARE trial is funded primarily by the National Heart, Lung, and Blood Institute (NHLBI), with additional funding from the ACGME. The NHLBI appointed an independent Data and Safety Monitoring

For peer review only - http://bmjopen.bmj.com/site/about/guidelines.xhtml

Board (DSMB) to advise the Institute regarding the trial's progress, monitor data quality and also to safeguard the interests of study participants.

Study Aims and Hypotheses

Study hypotheses are presented in Table 1. The primary hypothesis for the trial is that 30-day any-site patient mortality under FLEX will not exceed (will not be inferior to) 30-day patient mortality under STD, measured as the difference in difference across STD and FLEX programs between a program's 30-day patient mortality rate in the trial year and that rate in the pre-trial year (i.e., 30-day patient mortality rate at a program during the trial year minus 30-day patient mortality rate at the program in the pre-trial year). Secondary outcomes related to patient safety include 7-day and 30-day hospital readmission rates, complication rates, the probability of a prolonged length of stay, total resource utilization, and Medicare payments.

ICOMPARE's education hypotheses are that trainees in the FLEX arm will spend more time in direct patient care and education and have greater satisfaction with educational experiences compared to their STD arm peers; that standardized test scores for interns in FLEX will not be lower than such scores for interns in the STD arm; that faculty in the FLEX arm will report greater satisfaction with their clinical teaching experiences and greater perceptions of safety, teamwork and supervision than faculty in the STD arm.

ICOMPARE's sleep hypotheses are that the average daily sleep would not be less among interns in the FLEX arm compared to those in the STD arm, and that interns in the FLEX arm would not have greater subjective sleepiness or lower behavioral alertness than interns in the STD arm.

Methods and Analysis

Study Design

For peer review only - http://bmjopen.bmj.com/site/about/guidelines.xhtml

iCOMPARE is a cluster randomized trial to compare two alternative duty hour standards in 63 IM training programs in the United States fielded in academic year 2015-2016. Table 2 compares the duty hour standards between the FLEX and STD arms.

Cluster randomization occurred at the level of the residency program, the level at which duty hour policies are implemented. Although duty hour standards are mandated, individual programs vary considerably in how they schedule their trainees within those standards. The trial is pragmatic in that the intervention arm effectively represents the exposure of residency programs to an alternative set of duty hour standards. The exposure is the policy change, not the actual duty hours that are implemented in response. This approach is akin to clinical trials of outpatient pharmaceuticals in which the exposure is the prescription of the control or intervention drug, regardless of participant adherence.

Hypotheses regarding how interns spend their time require detailed time-motion observations, and hypotheses about interns' sleep and alertness require detailed observations of sleep cycles and psychomotor vigilance—both accomplished through substudies deployed in a sample of participating programs.

Sample Size and Power

The non-inferiority 30-day patient mortality hypothesis (H1a) is the trial's primary hypothesis. Defining the outcome as the trial year rate minus the pre-trial year rate adjusts each program's outcome for secular trends in 30-day mortality, as well as for differential program-to-program patient risk profiles. The variability in this outcome and the 30-day mortality rate in the STD group was estimated using Medicare data from 2008 for the population of target IM programs (i.e., all IM programs meeting the study-specific size and population criteria). Using a two-sample t-test for non-inferiority of the between group mean year over year difference in mortality and assuming 80% power, Type I error of 5%, a non-

For peer review only - http://bmjopen.bmj.com/site/about/guidelines.xhtml

inferiority margin of 1%, and a 30-day mortality rate of 11% ± 1.5% in STD, we calculated a required sample of 58 programs, 29 per treatment group.

This number of programs and the associated trainees and faculty provide excellent power for analyses investigating the education hypotheses which use person-level outcomes. Assuming 30 interns, 50 second year or higher trainees, 10 faculty members, and 1 director per program, iCOMPARE was projected to include 1740 interns, 2900 second year or higher trainees, 580 faculty members, and 58 program directors.

Hypothesis H2a addresses the Flex versus STD difference in percent of time spent by interns in direct patient care and was assessed with a Time-Motion study. The sample size of the Time-Motion study is 6 programs (3 FLEX, 3 STD; 60 interns, 10 interns per program). Preliminary data [27,28] suggested that the mean percent time spent in direct patient care in STD programs would be 13% ± 4%. The Time-Motion study has at least 80% power to detect a 3% difference between FLEX and STD in time spent in direct care.

Hypothesis H3a addresses the FLEX versus STD difference in average intern daily sleep over a 14-day period. Targeting 90% power, one-sided Type I error of 5%, a non-inferiority margin of 0.5 hours, and assuming expected average daily sleep in STD of 6.9 ± 1.5 hours [29] the sample size for the Sleep and Alertness study was calculated to be 290 interns (145 per treatment group) increased to 384 interns (192 per treatment group) to anticipate data loss related to non-adherence and dropouts.

Study Population and Inclusion Criteria

The CONSORT diagram is provided in Figure 1. In 2014 there were 379 IM ACGME-accredited training programs in the country not on probation. The 54 programs in New York State were excluded because that state's legislated duty hour standards were not subject to the waiver required for the intervention

For peer review only - http://bmjopen.bmj.com/site/about/guidelines.xhtml

arm. Since the patient safety outcomes would be measured in Medicare patients, Veterans Administration hospitals did not qualify for inclusion. Sufficiently precise estimates of our patient safety outcome measures necessitated exclusion of 84 programs training only at hospitals in the bottom 50% by trainee-to-bed ratio or the bottom 25% by patient volume in the measured diagnoses. An additional 62 programs in the lowest quartile of number of trainees were excluded to ensure measurement of enough trainees. Two exceptions were allowed by the Steering Committee. One program was selected for inclusion despite deviations in size of its two affiliated hospitals; together the two hospitals met the qualifying hospital size. A second program was selected for inclusion because the hospital it was affiliated with had far exceeded the trainee-to-bed ratio even though the patient volume was slightly low. The 179 resulting programs were eligible for inclusion.

Recruitment

Directors of eligible programs were invited to participate by letters of invitation and informational sessions during the Fall 2014 and Spring 2015 Association of Program Directors in Internal Medicine (APDIM) meetings. Participation required agreement to support study needs and a participation agreement signed by the Designated Institutional Official indicating approval from the institution's Graduate Medical Education Committee. Program directors indicated the hospitals, and the trainee rotations within those hospitals, which would participate in iCOMPARE. Each program was reviewed by the study team to confirm that at least one of the hospitals indicated by the program director qualified for inclusion in the trial. The study team checked the list of hospitals provided by the program director for shared staffing with another training program eligible for iCOMPARE so that those programs could be randomized together to avoid having different duty hour schedules for trainees at a common hospital. This occurred for 3 pairs of hospitals. In total, 63 programs agreed to participate and were randomized.

For peer review only

For peer review only - http://bmjopen.bmj.com/site/about/guidelines.xhtml

Institutional Review Board Determinations

The University of Pennsylvania Institutional Review Board (IRB) approved the iCOMPARE protocol and served as the IRB of record for all participating programs that agreed to sign an Institutional Affiliation Agreement. Forty programs chose this option and submitted the required paperwork. Twenty-three programs opted for a local review process.

Randomization

Randomization was 1:1 into the STD and FLEX arms. Participating programs in iCOMPARE were not masked to their assignment. Sixty programs completed the application process quickly and were randomized in November 2014 so that they could provide applicants interviewing for positions in academic year 2015-2016 information about the duty hour arm assignment. Randomization of programs ended in March 2015, judged to be the latest date by which a program could be randomized to the FLEX arm and have adequate time to adjust their residency schedules in anticipation of the 2015-2016 intervention year.

Data Sources

Hypothesis testing required outcome data from patients, trainees, and program directors and faculty. Because of the available and highly reliable patient mortality information (both in hospital and after discharge) in the Medicare fee for service (FFS) program, the iCOMPARE patient population was limited to Medicare FFS beneficiaries with a qualifying principal diagnosis on hospital admission (Supplementary Appendix Table 1); these diagnoses were chosen for their common treatment on internal medicine services (excluding oncology and neurology diagnoses) and their elevated mortality rates. Because similar data are not available for patients enrolled in a Medicare managed care program, patients

For peer review only - http://bmjopen.bmj.com/site/about/guidelines.xhtml

For peer review only

enrolled in a Medicare managed care program six months before or one month post discharge for the index hospital admission were excluded.

Randomized programs were further invited to participate in the observational Time-Motion and the Sleep and Alertness sub-studies, described below

Timeline

Trial development began in 2013. The Medicare data required for the analyses of the patient safety outcomes are not expected to be available until mid 2018 (Figure 2).

Outcomes, Measures, and Data Collection

Aim 1 – Patient Safety

Patient safety measures are derived from Medicare data, collected as part of routine clinical practice and administration and obtained through ResDAC (https://www.resdac.org/). The primary outcome is based on 30-day (from admission) all-location mortality, a patient-centered measure unaffected by length of hospitalization.[8,9,13,16,17,30,31] Secondary outcomes included prolonged length of stay,[12,32-34] 30-day readmission rate, [17,35] complication rate,[11,32,36,37] and resource utilization measures (cost) [15,33] and payment among fee-for-service Medicare beneficiaries with specific diagnoses.

Aim 2 – Education

Education measures are derived from multiple sources. The primary education measures are specified in Table 3 and come from the Time-Motion study, the ACGME year-end trainee and core faculty surveys, and the interns' In-Training Examination (ITE) scores provided by the American College of Physicians

For peer review only - http://bmjopen.bmj.com/site/about/guidelines.xhtml

(ACP). Secondary education measures include iCOMPARE-generated surveys and survey data provided by the Association of Program Directors in Internal Medicine (APDIM).

Time-Motion study. We recruited 3 IM programs randomized to STD and 3 randomized to FLEX to participate in direct observations of some of their interns, targeting programs in Philadelphia or Baltimore for operational convenience. Participating programs received $3,000 to support these sub-study activities. We recruited interns rotating on General Medicine services between March-May 2016. Eligible and interested interns provided written consent.

Twenty three observers (medical students and undergraduates) were trained to follow participating interns. They used a custom-built tablet-based software program to document start and stop times for various intern activities: direct patient care, indirect patient care, education, rounds, work, handoffs, and miscellaneous, each with various subcategories reflecting greater specificity of tasks. For example, direct patient care had subcategories for patient interactions, family interactions, and physical contact (e.g., physical exam). At least one activity had to be selected at all times, although more than one could be selected to reflect multitasking. At the start and stop of the shift an observer completed brief surveys that summarized total patient census numbers for that intern, including the numbers of transfers, discharges, admissions, and patients received at the beginning of a shift and handed-off at the end.

Shifts were selected at each site aiming to capture 30 shifts in a proportion mirroring – specific for each site – how interns generally spend their time on a general medicine inpatient rotation in a given week. Observers shadowed approximately 10-15 interns for 1-3 shifts per intern. Extended shifts (e.g., 24 hour call cycles) were often split by two observers. A 10% sample of shifts was observed simultaneously by two observers to estimate inter-rater reliability. Interns received a $50 Amazon gift card for each observed shift.

For peer review only - http://bmjopen.bmj.com/site/about/guidelines.xhtml

ACGME end of year survey data. The ACGME provided iCOMPARE with summary data derived from their end-of-year surveys of trainees and core faculty in May 2014, May 2015, and May 2016; all data provided by the ACGME to iCOMPARE was at the level of treatment group arm (FLEX or STD) and year collected (2014, 2015, 2016). All data were stripped of program identifiers and individual respondent identifiers. iCOMPARE identified the programs enrolled in iCOMPARE and their treatment group assignments to the ACGME and received summary treatment-group level information for the single question items specified in hypotheses H2b and H2c and many content areas (combined responses over several questions covering related content) that comprise secondary education outcomes.

ITE score data from ACP. The ACP provided iCOMPARE with trainee level ITE scores from 2015 and 2016; identifying information for a score was limited to PGY year and program identifier. The ACP provided scores for those trainees who had given permission to share their score for research purposes.

APDIM annual survey data. The APDIM provided data from its survey of program directors in fall 2015 and fall 2016. The deidentified data set did not include name of program or program director but did have a flag for STD or FLEX or not in study.

End-of-Shift Surveys. From August 31, 2015 to April 26, 2016, iCOMPARE conducted 16 two-week cycles of daily surveys administered to all trainees at all participating programs. At the start of each cycle, we randomized each trainee into one of 14 groups and then allocated each group to a day of the 2-week period to receive a survey so that each trainee was surveyed once every two weeks.

Surveys alternated between two sets of questions. Survey 1 was sent every other day to trainees asking: [1] the name of the rotation a trainee was on in the past 24 hours, specifying details such as inpatient or not, type of inpatient rotation, at main teaching hospital or other setting, or not in hospital; and if the trainee was on an inpatient rotation; [2] number of new patient evaluations completed in past 24 hours; [3] number of handoffs experienced in past 24 hours; and [4] the number of patients for which the

For peer review only - http://bmjopen.bmj.com/site/about/guidelines.xhtml

trainee was the primary provider. These questions provide another view of how interns spend their time.

Survey 2 was sent on the alternate days and asked the same first question as Daily Survey 1, as well as the trainee's ratings (too little, just right, too much) for [1] time spent in educational conference and related activities, [2] sense of ownership of patients, [3] work intensity, and [4] continuity of care. Data for these questions are related to satisfaction and complement the study's End-of-Year survey and ACGME survey described below.

Trainees were entered into an incentive lottery designed so that in each 2-week cycle, one intern and one resident at each of the 63 IM programs were each awarded either a $25 or $100 Amazon gift card if they had completed their survey during that period.

End-of-Year surveys. All trainees and program directors received iCOMPARE study-specific surveys in May-June 2015 (baseline) and again in May-June 2016 (post-intervention), administered via Lime Survey (https://www.limesurvey.org/). Invitations with personalized links were emailed during May of 2015 and May of 2016 with up to 6 reminders to nonrespondents. At the end of the intervention year, a $2,500 cash incentive was provided to each of the 9 programs with the highest response rates.

The trainee survey was administered to all trainees with only slight differences between versions for interns and PGY2 and higher trainees. The instrument was initially developed for the FIRST trial[26] (available online) and included items on trainee satisfaction, experience of duty hours, supervision, fatigue management, and resident and patient safety, and ended with the Maslach Burnout Inventory-Human Services Survey (MBI-HSS).[38] The MBI-HSS is a 22-item rating scale assessing three domains: emotional exhaustion (9 items), depersonalization (5 items), and lack of personal accomplishment (8 items). Items are answered on a frequency scale of 0 to 6 where 0 indicates never and 6 indicates every day.

For peer review only - http://bmjopen.bmj.com/site/about/guidelines.xhtml

The program director survey (available online)was modeled from an earlier survey to program directors [39] and included items on resident and faculty workload, resident morale, continuity, education, patient safety, and program finances and administration.

Aim 3 – Sleep and alertness

Outcomes for the third aim include sleep duration and both subjective and objective measures of alertness among interns at 6 sites randomized to STD and 6 sites randomized to FLEX. At each of these 12 sites, program coordinators recruited interns scheduled to be on general medicine, medical intensive care, cardiology, or cardiac care rotations. Each participating program received $8,000 to cover the costs associated with scheduling interns for data acquisition and managing study equipment. Data collection spanned November 5, 2015 to May 31, 2016.

Interns providing written consent underwent 14-days of continuous measurements of rest and activity via wrist actigraphs (model wGT3X-BT, The Actigraph Corp., Pensacola, FL).[40-42] Interns were instructed to wear the actigraph continuously, even on days off, except during activities that might damage it (e.g., water immersion or contact sports) or that would impede the delivery of clinical care. They were asked to remove the actigraph for up to 2 waking hours for recharging on days 1 and 7. Each participating intern received a $10 Amazon gift card for each day for which data were received.

Each morning, interns completed a brief online survey including the name of the shift an intern was working; a log reflecting sleep times and quality, and experiences of excessive sleepiness; and the Karolinska Sleepiness Scale.[43] Interns then completed a 3-minute Psychomotor Vigilance Test (PVT-B) [44,45] on an Android Smartphone (Samsung Galaxy SIII Neo). The PVT-B is based on simple reaction time to stimuli occurring at random inter-stimulus intervals and is the gold standard for measuring the neurobehavioral effects of acute and chronic sleep loss and circadian misalignment.[46] Actigraphy, survey, and PVT-B data were automatically encrypted and uploaded to a secure server and checked daily

For peer review only - http://bmjopen.bmj.com/site/about/guidelines.xhtml

for protocol adherence and potential technical issues with the equipment. If problems were detected or no data were received at all, interns were contacted by the study team to resolve any difficulties interns were having with equipment. Details of scoring are presented in the supplementary appendix materials 2: Sleep Actigraphy Scoring and supplementary appendix Figure 1 and Figure 2.

Statistical Considerations

Non-inferiority tests will be one-sided and superiority tests will be two-sided. All primary analyses will compare the FLEX and STD treatment groups as randomized, regardless of adherence to the assigned duty hour standards, according to the intention-to-treat (ITT) principle. Since directors at programs assigned to FLEX have considerable latitude in design of trainee schedules, we expect variation amongst the duty-hour schedules followed in the FLEX group. Protocol-specified secondary analyses addressing the degree of difference between FLEX and STD schedules will be completed. We will also report mortality results adjusting for the clinical condition associated with patient's principal diagnosis as well as demographic variables and comorbidities determined using a 6-month look-back period. Similar approaches will be used for the other patient safety outcomes specified in hypotheses H1b-H1e.

The outcome measures for the education hypotheses (H2a-H2d) and the sleep and alertness hypotheses (H3a-H3b) are person-level (intern, trainee, or faculty) measures. For each outcome, the repeated measures on an individual will be averaged, which eliminates within-person correlations. Each person-level mean will be the response variable in a mixed random effects linear regression model with a single fixed term for treatment group and with restricted maximum likelihood estimation of the random IM program effect as implemented in SAS (Cary, NC) or STATA (College Station, TX) to account for within program correlations in outcome measures. When pre-trial year data are available, additional analyses will adjust an individual's trial year mean response for the pre-trial year program-level mean response (i.e., a difference of differences analysis). The pre-trial program level mean response must be used since

For peer review only - http://bmjopen.bmj.com/site/about/guidelines.xhtml

the trainees and faculty providing data in the pre-trial year are not the same trainees and faculty providing data in the trial year, but are at the same IM program.

Ethics and Dissemination

The University of Pennsylvania Institutional Review Board (IRB) approved the iCOMPARE protocol and served as the IRB of record for all participating programs that agreed to sign an Institutional Affiliation Agreement. Forty programs chose this option and submitted the required paperwork. Twenty-three programs opted for a local review process.

Plans for dissemination include submitting results for the various aims to academic meetings and peer-reviewed publications as they become available. A Data Safety Monitoring Board will review and approve results related to the primary hypotheses before dissemination.

Discussion

iCOMPARE is a one-year, cluster-randomized, pragmatic trial designed to evaluate the availability of an alternative resident duty hour schedule for effects on patient safety, resident education, and intern sleep and alertness. iCOMPARE's design is similar to that of the FIRST trial conducted among surgical trainees. What distinguishes both of these trials from prior work looking at duty hour schedules, in addition to their large size, comprehensive approach, and immediate policy relevance, is that they help elevate the standards of evidence applied to graduate medical education policy more generally. Although randomized trials provide strong evidence for the causal inference required for good policy, they typically answer narrow questions. Those questions today revolve around the length of shifts within an 80-hour work week. Inside the field of graduate medical education, these issues have been hotly debated.

For peer review only - http://bmjopen.bmj.com/site/about/guidelines.xhtml

With the results of the FIRST trial demonstrating non-inferiority in patient outcomes when a more flexible schedule was available, the ACGME issued new duty hour standards, effective July 2018, that correspond to the intervention arm of iCOMPARE.[47]

Given that surgery and internal medicine are large fields with many residents caring for many patients, it is important to study the duty hour rules in both specialties as surgical and medical training programs differ in structure, process, culture, the kinds of residents they attract, the patients they serve, and the duties of trainees.

Conclusions

There is considerable interest in understanding concerns in the US health care system, such as why US health care is so expensive, why its overall effects on health lag behind those of peer nations in particular with respect to safety, and why its individual effects are so unevenly distributed. In contrast, relatively little effort has been devoted to studying how physicians are trained—surprising because although physicians are certainly not the only reason for the concerns, it seems evident that physician training must play an important role in most outcomes related to US health care. In that broad context, iCOMPARE promises narrow but substantive evidence to inform resident duty hour standards in internal medicine and signals new interest in methodologically stronger research in medical education.

For peer review only - http://bmjopen.bmj.com/site/about/guidelines.xhtml

References

1. Friedman RC, Bigger JT, Kornfeld DS. The intern and sleep loss. *N Engl J Med* 1971;285(4):201–3.
2. Asch DA, Parker RM. The Libby Zion case: One step forward or two steps backward? *N Engl J Med* 1988;318:771-5.
3. Ulmer C, Wolman DM, Johns MME, eds. Resident Duty Hours: Enhancing Sleep, Supervision, and Safety. National Academies Press: Washington, DC. 2009.
4. Landrigan CP, Rothschild JM, Cronin JW, et al. Effects of reducing interns' work hours on serious medical errors in intensive care units. *N Engl J Med* 2004;351(18):1838-48.
5. Asch DA, Bilimoria KY, Desai SV. Resident duty hours and medical education policy — raising the evidence bar. *N Engl J Med* 2017;376:1704-6.
6. Petersen LA, Brennan TA, O'Neil AC, Cook EF, Lee TH. Does housestaff discontinuity of care increase the risk for preventable adverse events? *Ann Intern Med* 1994;121(11):866-72.
7. Kitch BT, Cooper JB, Zapol WM, et al. Handoffs causing patient harm: a survey of medical and surgical house staff. *Jt Comm J Qual Patient Saf* 2008;34(10):563-70.
8. Volpp KG, Rosen AK, Rosenbaum PR, et al. Mortality among hospitalized Medicare beneficiaries in the first 2 years following ACGME resident duty hour reform. *JAMA* 2007;298:975-83.
9. Volpp KG, Rosen AK, Rosenbaum PR, et al. Mortality among patients in VA hospitals in the first 2 years following ACGME resident duty hour reform. *JAMA* 2007;298:984-92.
10. Prasad M, Iwashyna TJ, Christie JD, et al. Effect of work-hours regulations on intensive care unit mortality in United States teaching hospitals. *Crit Care Med* 2009; 37:2564-69.
11. Rosen AK, Loveland SA, Romano PS, et al. Effects of resident duty hour reform on surgical and procedural patient safety indicators among hospitalized Veterans Health Administration and Medicare patients. *Med Care* 2009; 47:723-31.
12. Silber JH, Rosenbaum PR, Rosen AK, et al. Prolonged hospital stay and the resident duty hour

For peer review only - http://bmjopen.bmj.com/site/about/guidelines.xhtml

rules of 2003. *Med Care* 2009; 47:1191-200.

13. Volpp KG, Rosen AK, Rosenbaum PR, et al. Did duty hour reform lead to better outcomes among the highest risk patients? *J Gen Intern Med* 2009; 24:1149-55.
14. Press MJ, Silber JH, Rosen AK, et al. The impact of resident duty hour reform on hospital readmission rates among Medicare beneficiaries. *J Gen Intern Med* 2011; 26:405-11.
15. Navathe AS, Silber JH, Small DS, et al. Teaching hospital financial status and patient outcomes following ACGME duty hour reform. *Health Serv Res* 2013; 48:476-98.
16. Volpp KG, Small DS, Romano PS, et al. Teaching hospital five-year mortality trends in the wake of duty hour reforms. *J Gen Intern Med* 2013; 28:1048-55.
17. Patel MS, Volpp KG, Small DS, et al. Association of the 2011 ACGME resident duty hour reforms with mortality and readmissions among hospitalized Medicare patients. *JAMA* 2014; 312:2364-73.
18. Block L, Habicht R, Wu AW, et al. In the wake of the 2003 and 2011 duty hours regulations, how do internal medicine interns spend their time? *J Gen Intern Med* 2013;28(8):1042-47.
19. Desai SV, Feldman L, Brown L, et al. Effect of the 2011 vs 2003 duty hour regulation-compliant models on sleep duration, trainee education, and continuity of patient care among internal medicine house staff: a randomized trial. *JAMA Intern Med* 2013;173(8):649-55.
20. Sen S, Kranzler HR, Didwania AK, et al. Effects of the 2011 duty hour reforms on interns and their patients: a prospective longitudinal cohort study. *JAMA Intern Med* 2013;173(8):657-63.
21. Silber JH, Romano PS, Itani KM, et al. Assessing the effects of the 2003 resident duty hours reform on internal medicine board scores. *Acad Med* 2014; 89:644-51.
22. Drolet BC, Christopher DA, Fischer SA. Residents' response to duty-hour regulations--a follow-up national survey. *N Engl J Med* 2012;366(24):e35.

For peer review only - http://bmjopen.bmj.com/site/about/guidelines.xhtml

For peer review only

23. Drolet BC, Khokhar MT, Fischer SA. The 2011 duty-hour requirements--a survey of residency program directors. *N Engl J Med* 2013;368(8):694-97.
24. Drolet BC, Spalluto LB, Fischer SA. Residents' perspectives on ACGME regulation of supervision and duty hours--a national survey. *N Engl J Med* 2010;363(23):e34.
25. Bilimoria KY, Chung JW, Hedges LV, et al. Development of the flexibility in duty hour requirements for surgical trainees (FIRST) trial protocol: A national cluster-randomized trial of resident duty hour policies. *JAMA Surg* 2016;151:273-81.
26. Bilimoria KY, Chung JW, Hedges LV, et al. National cluster-randomized trial of duty-hour flexibility in surgical training. *N Engl J Med* 2016; 374:713-27.
27. Fletcher KE, Visotcky AM, Slagle JM, et al. The composition of intern work while on call*. J Gen Intern Med* 2012;27(11):1432-7.
28. Block L, Habicht R, Wu AW, et al. In the wake of the 2003 and 2011 duty hours regulations, how do internal medicine interns spend their time? *J Gen Intern Med* 2013;28:1042-7.
29. Volpp KG, Shea JA, Small DS, et al. Effect of a protected sleep period on hours slept during extended overnight in-hospital duty hours among medical interns. *JAMA* 2012;308(21):2208-17.
30. Drye E, Normand SL, Wang Y, et al. Comparison of hospital risk-standardized mortality rates calculated by using in-hospital and 30-day models: implications for hospital profiling. *Ann Intern Med* 2012;156:19-26.
31. Meehan TP, Fine MJ, Krumholz HM, et al. Quality of care, process, and outcomes in elderly patients with pneumonia. *JAMA* 1997;278:2080-4.
32. Silber JH, Rosenbaum PR, Even-Shoshan O, et al. Length of stay, conditional length of stay, and prolonged stay in pediatric asthma. *Health Serv Res* 2003;38:867-86.
33. Silber JH, Rosenbaum PR, Kelz RR, et al. Medical and financial risks associated with surgery in the elderly obese. *Ann Surg* 2012;256:79-86.

For peer review only - http://bmjopen.bmj.com/site/about/guidelines.xhtml

34. Silber JH, Rosenbaum PR, Koziol LF, Sutaria N, Marsh RR, Even-Shoshan O. Conditional length of stay. *Health Serv Res* 1999;34:349-63.

35. Krumholz HM, Lin Z, Keenan PS, et al. Relationship between hospital readmission and mortality rates for patients hospitalized with acute myocardial infarction, heart failure, or pneumonia. *JAMA* 2013;309:587-93.

36. Iezzoni LI, Daley J, Heeren T, et al. Using administrative data to screen hospitals for high complication rates. *Inquiry* 1994;31:40-55.

37. McCarthy EP, Iezzoni LI, Davis RB, et al. Does clinical evidence support ICD-9-CM diagnosis coding of complications? *Med Care* 2000;38:868-76.

38. Maslach C, Jackson SE, Leiter MP. Maslach Burnout Inventory. 3rd Ed. Palo Alto: Consulting Psychologists Press. 1996.

39. Shea JA, Willett LL, Borman KR, et al. Anticipated consequences of the 2011 duty hour standards: A view from surgery and medicine program directors. *Acad Med* 2012:87;895-903.

40. Ancoli-Israel S, Cole R, Alessi C, et al. The role of actigraphy in the study of sleep and circadian rhythms. *Sleep* 2003;26(3):342-92

41. Malmberg B, Kecklund G, Karlson B, et al. Sleep and recovery in physicians on night call: a longitudinal field study. *BMC Health Serv Res* 2010;10(239).

42. Basner M, Dinges DF, Shea JA, et al. Sleep and Alertness in Medical Interns and Residents: An observational study on the role of extended shifts. *Sleep* 2017;40(4). doi: 10.1093/sleep/zsx027

43. Akerstedt T, Gillberg M. Subjective and objective sleepiness in the active individual. *Int J Neurosci* 1990;52(1-2):29-37.

44. Basner M, Mollicone DJ, Dinges DF. Validity and sensitivity of a brief Psychomotor Vigilance Test (PVT-B) to total and partial sleep deprivation. *Acta Astronautica* 2011;69:949-59.

45. Basner M, Dinges DF. Maximizing sensitivity of the Psychomotor Vigilance Test (PVT) to sleep

For peer review only - http://bmjopen.bmj.com/site/about/guidelines.xhtml

For peer review only

loss. *Sleep* 2011;34(5):581-91.

46. Dinges DF, Maislin G, Hanowski RJ, et al. Commercial Motor Vehicle (CMV) Driver Restart Study: Final Report. U.S. DoT; Federal Motor Carrier Safety Administration; Office of Analysis, Research, and Technology. 2016.

47. The ACGME Common Program Requirements Section VI revisions from TJ Nasca, MD. ACGME, 2017. (Accessed August 15, 2017, at http://www.acgme.org/Portals/0/PDFs/Nasca-Community/Section-VI-Memo-3-10-17.pdf.)

For peer review only

For peer review only - http://bmjopen.bmj.com/site/about/guidelines.xhtml

Authors' contributions

Judy A. Shea, PhD is a co-investigator. She took the lead in drafting this paper and incorporating all co-authors' suggestions on subsequent drafts. She also worked closely with Drs. Desai and Bellini to design the refine the aims and methods related to the education aim.

Jeffrey H. Silber, MD, PHD took the lead on writing the protocol for the patient safety component of the protocol. He reviewed and commented on multiple version of this manuscript.

Sanjay V. Desai, MD worked with Drs. Shea and Bellini to develop the details for the education aim and outcomes. He reviewed and commented on multiple version of this manuscript.

David F Dinges, PhD, took the lead and worked with Dr. Basner to develop the details for the sleep and alertness aim and outcomes. He reviewed and commented on multiple version of this manuscript.

Lisa M.Bellini, MD worked with Dr. Shea and Desai to develop the details for the education aim and outcomes. She reviewed and commented on multiple version of this manuscript.

James Tonascia, PhD took the lead and worked with Alice L Sternberg, ScM, Dylan S. Small, PhD and David M Shade, JD to develop the sample size calculations and analytic plan for all study aims. They each reviewed and commented on multiple version of this manuscript.

Joel T. Katz, MD participated in design of the direct observation portion of the protocol and commented on multiple versions of the manuscript.

Mathias Basner, MD, PhD worked with Dr. Dinges to develop the details for the sleep and alertness aim and outcomes. He reviewed and commented on multiple version of this manuscript.

For peer review only

Krisda H. Chaiyachati, MD, MPH took the lead in developing the details for the direct observation potion of the protocol. He reviewed and commented on multiple version of this manuscript.

Orit Even-Shoshan, MS worked with Dr. Silber to design the patient safety portion of the protocol.

David W. Bates, MD participated in design of the direct observation portion of the protocol and commented on multiple versions of the manuscript.

Kevin G. Volpp, MD, PhD worked on the overall development of the protocol and commented on versions of the manuscript.

David A. Asch, MD, MBA is the principal investigator for the iCOMPARE protocol and has been instrumental in all phases of protocol design and study implementation.

This work is supported by the National Heart, Lung, and Blood Institute (NHLBI), project number 5U01HL125388. This paper is subject to NIH Public Access Policy

Trial Registration: clinicaltrials.org Identifier: NCT02274818.

The authors report no competing interests.

For peer review only

For peer review only - http://bmjopen.bmj.com/site/about/guidelines.xhtml

Figure 1 Legend: Consort Diagram

Figure 2 Legend: iCOMPARE Trial Timeline.

Supplementary Appendix Figure 1: Overview of Actigraphy and PVT scoring and review process

Supplementary Appendix Figure 2: Sleep Scoring Matrix

For peer review only

For peer review only - http://bmjopen.bmj.com/site/about/guidelines.xhtml

Table 1 Study Aims and Hypotheses

Primary Hypothesis:

Specific Aim 1: Examine patient safety and costs of care under STD and FLEX duty hour schedules.

H1a: (Primary hypothesis) 30-day patient mortality under FLEX will not exceed (will not be inferior to) mortality under STD.

Secondary Hypotheses:

H1b: 7-day and 30-day hospital readmission rates under FLEX will not exceed (will not be inferior to) the rates under STD.

H1c: Complication rates, defined by selected AHRQ Patient Safety Indicators, under Flex will not exceed (will not be inferior to) complication rates under STD.

H1d: The rate of prolonged length of stay under FLEX will not exceed (will not be inferior to) the rate of prolonged length of stay under STD.

H1e: Total costs and Medicare payments, under FLEX will not exceed (will not be inferior to) overall costs under STD.

Specific Aim 2: Examine the quality of education under STD and FLEX duty hour schedules.

Secondary Hypotheses:

H2a: Interns in FLEX will spend greater time in direct patient care and education compared to interns in STD.

H2b: Trainees in FLEX will report greater satisfaction with their educational experience (greater ownership, greater continuity and lower burnout) than trainees in STD.

H2c: Faculty in FLEX will report greater satisfaction with their clinical teaching experiences and greater perceptions of safety, teamwork and supervision than faculty in STD.

H2d: Standardized test scores for interns in Flex will not be less than (inferior to) those for interns in STD.

For peer review only

For peer review only - http://bmjopen.bmj.com/site/about/guidelines.xhtml

Specific Aim 3: Examine intern sleep time and alertness under STD and FLEX duty hour schedules.

Secondary Hypotheses:

H3a: Average daily sleep obtained by interns in FLEX will not be less than (will not be inferior to) that of interns in STD, as determined by a 14-day period of sleep monitoring using actigraphy and daily sleep diaries.

H3b: Interns in FLEX will not have (will not be inferior to) greater average subjective sleepiness via the Karolinska Sleepiness Scale (KSS)(24), or lower average behavioral alertness via the Psychomotor Vigilance Test (PVT)(25) than interns in STD, as determined by a 14-day period of morning sleepiness-alertness monitoring.

For peer review only

For peer review only - http://bmjopen.bmj.com/site/about/guidelines.xhtml

Table 2. Overview of Usual Care and Intervention Arm Duty Hour Requirements

Requirement	Usual Care (STD; current duty hour standards)	Intervention (FLEX; flexible duty hour standards)
80 hours per week (averaged over 4 weeks)	No change	No change
1 day off per week (averaged over 4 weeks)	No change	No change
In-house call no more than every 3rd night (averaged over 4-weeks)	No change	No change
PGY-1 resident duty hour periods must not exceed 16 hours	No change	Eliminated
PGY-2 and above must not work more than 24 hours with an additional 4 hours permitted for transitions in care	No change	Eliminated
Residents must have 14 hours off after 24 hours in-house duty and at least 8-10 hours off after a regular shift	No change	Eliminated

Adapted from Bilimoria KY, Chung JW, Hedges LV, et al. Development of the flexibility in duty hour requirements for surgical trainees (FIRST) trial protocol. JAMA Surg. 2016;151:273-81.

For peer review only

For peer review only - http://bmjopen.bmj.com/site/about/guidelines.xhtml

Table 3: Operationalization of Primary Education Measures

Hypothesis	Primary Data Source	Who	Definition
2a – time in direct patient care and education	Time-motion study	PGY1	percent of observed shift minutes spent in each activity
2b – Trainee satisfaction	ACGME year-end trainee survey	PGY1-3	a single item on the ACGME resident survey re: appropriate balance for education with response options of never, rarely, sometimes, often, very often
2c – Program director satisfaction	ACGME core faculty survey	Core faculty	a single item on the ACGME faculty survey that asked about residents'/fellows' workload exceeding the capacity to do the work with response options of never, rarely, sometimes, often, very often
2d- Standardized test scores	ACP in-training examination scores	PGY1	percent of questions answered correctly

For peer review only

For peer review only - http://bmjopen.bmj.com/site/about/guidelines.xhtml

For peer review only

For peer review only - http://bmjopen.bmj.com/site/about/guidelines.xhtml

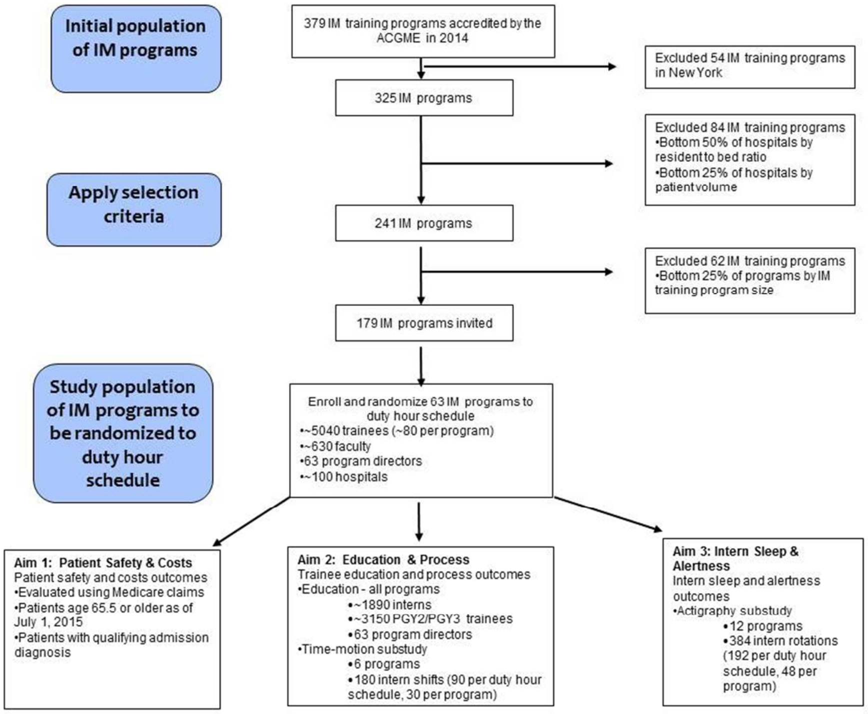

Figure 1 Legend: Consort Diagram

254x190mm (96 x 96 DPI)

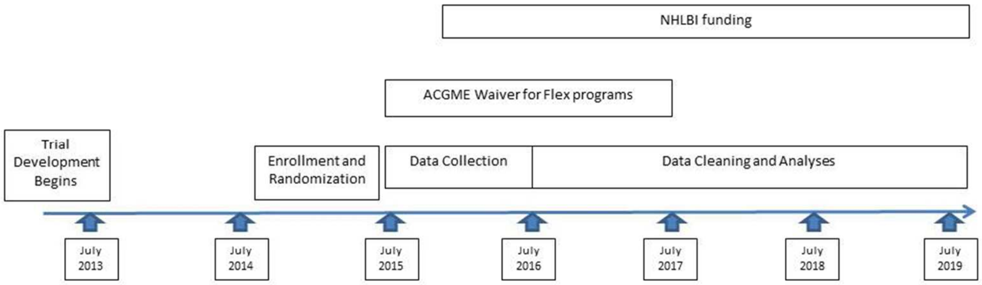

Figure 2 Legend: iCOMPARE Trial Timeline

254x190mm (96 x 96 DPI)

Supplementary materials

Appendix Table 1. ICD-9 codes for principal diagnoses qualifying hospital/patient for inclusion in iCOMPARE randomization/analysis

Pneumonia:

481	Pneumococcal Pneumonia [Streptococcus Pneumoniae Pneumonia]
482	Other Bacterial Pneumonia
482.1	Pneumonia Due to Pseudomonas
482.2	Pneumonia Due to Hemophilus Influenzae (H. Influenzae)
482.3	Pneumonia Due to Streptococcus
482.31	Pneumonia Due to Streptococcus Group A
482.32	Pneumonia Due to Streptococcus Group B
482.39	Pneumonia Due to Other Streptococcus
482.41	Methicillin Susceptible Pneumonia Due to Staphylococcus Aureus
482.42	Methicillin Resistant Pneumonia Due to Staphylococcus Aureus
482.49	Other Staphylococcus Pneumonia
482.82	Pneumonia Due to Escherichia Coli [E.Coli]
482.83	Pneumonia Due to Other Gram-Negative Bacteria
482.84	Pneumonia Due to Legionnaires' Disease
482.89	Pneumonia Due to Other Specified Bacteria
482.4	Pneumonia Due to Staphylococcus
482.9	Bacterial Pneumonia Unspecified
483	Pneumonia Due to Other Specified Organism

For peer review only - http://bmjopen.bmj.com/site/about/guidelines.xhtml

483.1	Pneumonia Due to Chlamydia
483.8	Pneumonia Due to Other Specified Organism
485	Bronchopneumonia Organism Unspecified
486	Pneumonia Organism Unspecified

Stroke:

430	Subarachnoid Hemorrhage
431	Intracerebral Hemorrhage
432.0	Nontraumatic Extradural Hemorrhage
432.1	Subdural Hemorrhage
432.9	Unspecified Intracranial Hemorrhage
433.01	Occlusion and Stenosis of Basilar Artery with Cerebral Infarction
433.11	Occlusion and Stenosis of Carotid Artery with Cerebral Infarction
433.21	Occlusion and Stenosis of Vertebral Artery with Cerebral Infarction
433.31	Occlusion and Stenosis of Multiple and Bilateral Precerebral Arteries with Cerebral Infarction
433.81	Occlusion and Stenosis of Other Specified Precerebral Artery with Cerebral Infarction
433.91	Occlusion and Stenosis of Unspecified Precerebral Artery with Cerebral Infarction
434.01	Cerebral Thrombosis with Cerebral Infarction
434.11	Cerebral Embolism with Cerebral Infarction
434.91	Cerebral Artery Occlusion Unspecified with Cerebral Infarction
436	Acute but Ill-Defined Cerebrovascular Disease

For peer review only

For peer review only - http://bmjopen.bmj.com/site/about/guidelines.xhtml

Acute Myocardial Infarction:

410.01 Acute Myocardial Infarction of Anterolateral Wall Initial Episode of Care

410.11 Acute Myocardial Infarction of Other Anterior Wall Initial Episode of Care

410.21 Acute Myocardial Infarction of Inferolateral Wall Initial Episode of Care

410.31 Acute Myocardial Infarction of Inferoposterior Wall Initial Episode of Care

410.41 Acute Myocardial Infarction of Other Inferior Wall Initial Episode of Care

410.51 Acute Myocardial Infarction of Other Lateral Wall Initial Episode of Care

410.61 True Posterior Wall Infarction Initial Episode of Care

410.71 Subendocardial Infarction Initial Episode of Care

410.81 Acute Myocardial Infarction of Other Specified Sites Initial Episode of Care

410.91 Acute Myocardial Infarction of Unspecified Site Initial Episode of Care

Gastrointestinal Hemmorhage:

456.0 Esophageal Varices with Bleeding

530.7 Gastroesophageal Laceration-Hemorrhage Syndrome

530.82 Esophageal Hemorrhage

531.00 Acute Gastric Ulcer with Hemorrhage Without Obstruction

531.01 Acute Gastric Ulcer with Hemorrhage with Obstruction

531.20 Acute Gastric Ulcer with Hemorrhage and Perforation Without Obstruction

531.21 Acute Gastric Ulcer with Hemorrhage and Perforation with Obstruction

531.40 Chronic or Unspecified Gastric Ulcer with Hemorrhage Without Obstruction

531.41 Chronic or Unspecified Gastric Ulcer with Hemorrhage with Obstruction

531.60 Chronic or Unspecified Gastric Ulcer with Hemorrhage and Perforation Without Obstruction

For peer review only - http://bmjopen.bmj.com/site/about/guidelines.xhtml

531.61	Chronic or Unspecified Gastric Ulcer with Hemorrhage and Perforation with Obstruction
532.00	Acute Duodenal Ulcer with Hemorrhage Without Obstruction
532.01	Acute Duodenal Ulcer with Hemorrhage with Obstruction
532.20	Acute Duodenal Ulcer with Hemorrhage and Perforation Without Obstruction
532.21	Acute Duodenal Ulcer with Hemorrhage and Perforation with Obstruction
532.40	Chronic or Unspecified Duodenal Ulcer with Hemorrhage Without Obstruction
532.41	Chronic or Unspecified Duodenal Ulcer with Hemorrhage with Obstruction
532.60	Chronic or Unspecified Duodenal Ulcer with Hemorrhage and Perforation Without Obstruction
532.61	Chronic or Unspecified Duodenal Ulcer with Hemorrhage and Perforation with Obstruction
533.00	Acute Peptic Ulcer of Unspecified Site with Hemorrhage Without Obstruction
533.01	Acute Peptic Ulcer of Unspecified Site with Hemorrhage with Obstruction
533.20	Acute Peptic Ulcer of Unspecified Site with Hemorrhage and Perforation Without Obstruction
533.21	Acute Peptic Ulcer of Unspecified Site with Hemorrhage and Perforation with Obstruction
533.40	Chronic or Unspecified Peptic Ulcer of Unspecified Site with Hemorrhage Without Obstruction
533.41	Chronic or Unspecified Peptic Ulcer of Unspecified Site with Hemorrhage with Obstruction
533.60	Chronic or Unspecified Peptic Ulcer of Unspecified Site with Hemorrhage and Perforation Without Obstruction

For peer review only

For peer review only - http://bmjopen.bmj.com/site/about/guidelines.xhtml

533.61	Chronic or Unspecified Peptic Ulcer of Unspecified Site with Hemorrhage and Perforation with Obstruction
534.00	Acute Gastrojejunal Ulcer with Hemorrhage Without Obstruction
534.01	Acute Gastrojejunal Ulcer with Hemorrhage with Obstruction
534.20	Acute Gastrojejunal Ulcer with Hemorrhage and Perforation Without Obstruction
534.21	Acute Gastrojejunal Ulcer with Hemorrhage and Perforation with Obstruction
534.40	Chronic or Unspecified Gastrojejunal Ulcer with Hemorrhage Without Obstruction
534.41	Chronic or Unspecified Gastrojejunal Ulcer with Hemorrhage with Obstruction
534.60	Chronic or Unspecified Gastrojejunal Ulcer with Hemorrhage and Perforation Without Obstruction
534.61	Chronic or Unspecified Gastrojejunal Ulcer with Hemorrhage and Perforation with Obstruction
535.01	Acute Gastritis with Hemorrhage
535.11	Atrophic Gastritis with Hemorrhage
535.21	Gastric Mucosal Hypertrophy with Hemorrhage
535.31	Alcoholic Gastritis with Hemorrhage
535.41	Other Specified Gastritis with Hemorrhage
535.51	Unspecified Gastritis and Gastroduodenitis with Hemorrhage
535.61	Duodenitis with Hemorrhage
537.83	Angiodysplasia of Stomach and Duodenum with Hemorrhage
562.02	Diverticulosis of Small Intestine with Hemorrhage
562.03	Diverticulitis of Small Intestine with Hemorrhage
562.12	Diverticulosis of Colon with Hemorrhage
562.13	Diverticulitis of Colon with Hemorrhage

For peer review only

For peer review only - http://bmjopen.bmj.com/site/about/guidelines.xhtml

569.3	Hemorrhage of Rectum and Anus
569.85	Angiodysplasia of Intestine with Hemorrhage
578.0	Hematemesis
578.1	Blood In Stool
578.9	Hemorrhage of Gastrointestinal Tract Unspecified

Heart Failure:

398.91	Rheumatic Heart Failure (Congestive)
402.01	Malignant Hypertensive Heart Disease with Heart Failure
402.11	Benign Hypertensive Heart Disease with Heart Failure
402.91	Unspecified Hypertensive Heart Disease with Heart Failure
404.01	Hypertensive Heart and Chronic Kidney Disease Malignant with Heart Failure with Chronic Kidney Disease Stage I Through Stage Iv or Unspecified
404.03	Hypertensive Heart and Chronic Kidney Disease Malignant with Heart Failure with Chronic Kidney Disease Stage V or End Stage Renal Disease
404.11	Hypertensive Heart and Chronic Kidney Disease Benign with Heart Failure with Chronic Kidney Disease Stage I Through Stage Iv or Unspecified
404.13	Hypertensive Heart and Chronic Kidney Disease Benign with Heart Failure with Chronic Kidney Disease Stage V or End Stage Renal Disease
404.91	Hypertensive Heart and Chronic Kidney Disease Unspecified with Heart Failure with Chronic Kidney Disease Stage I Through Stage Iv or Unspecified
404.93	Hypertensive Heart and Chronic Kidney Disease Unspecified with Heart Failure with Chronic Kidney Disease Stage V or End Stage Renal Disease
428	Heart Failure

For peer review only - http://bmjopen.bmj.com/site/about/guidelines.xhtml

428.0	Congestive Heart Failure Unspecified
428.1	Left Heart Failure
428.20	Unspecified Systolic Heart Failure
428.21	Acute Systolic Heart Failure
428.22	Chronic Systolic Heart Failure
428.23	Acute on Chronic Systolic Heart Failure
428.30	Unspecified Diastolic Heart Failure
428.31	Acute Diastolic Heart Failure
428.32	Chronic Diastolic Heart Failure
428.33	Acute on Chronic Diastolic Heart Failure
428.40	Unspecified Combined Systolic and Diastolic Heart Failure
428.41	Acute Combined Systolic and Diastolic Heart Failure
428.42	Chronic Combined Systolic and Diastolic Heart Failure
428.43	Acute on Chronic Combined Systolic and Diastolic Heart Failure
428.9	Heart Failure Unspecified

Septicemia:

038	Septicemia
038.0	Streptococcal Septicemia
038.1	Staphylococcal Septicemia
038.9	Unspecified Septicemia

Kidney Failure:

584	Acute Kidney Failure

For peer review only

For peer review only - http://bmjopen.bmj.com/site/about/guidelines.xhtml

584.9	Acute Kidney Failure Unspecified

Cardiac Disease:

427.31	Atrial Fibrillation
427.41	Ventricular Fibrillation
427.5	Cardiac Arrest

Chronic Obstructive Pulmonary Disease:

490	Bronchitis Not Specified as Acute or Chronic
491.21	Obstructive Chronic Bronchitis with (Acute) Exacerbation
491.22	Obstructive Chronic Bronchitis with Acute Bronchitis

Pancreatitis:

577.0	Acute Pancreatitis
577.9	Unspecified Disease of Pancreas

Acute Respiratory Failure:

518.81	Acute Respiratory Failure
518.84	Acute and Chronic Respiratory Failure
518.89	Other Diseases of Lung Not Elsewhere Classified
519.11	Acute Bronchospasm

Chest Pain:

786.5	Chest Pain

For peer review only

For peer review only - http://bmjopen.bmj.com/site/about/guidelines.xhtml

786.59	Other Chest Pain

Cellulitis:

682	Other Cellulitis and Abscess
682.6	Cellulitis and Abscess of Leg Except Foot

Coronary Atherosclerosis:

414.01	Coronary Atherosclerosis of Native Coronary Artery
414.1	Aneurysm and Dissection of Heart

Pulmonary Embolism:

415	Acute Pulmonary Heart Disease
415.12	Septic Pulmonary Embolism
415.19	Other Pulmonary Embolism and Infarction

Syncope:

780.0	Alteration of Consciousness
780.2	Syncope and Collapse
780.3	Convulsions
780.01	Coma

Intestinal Infection:

008	Intestinal Infections Due to Other Organisms
008.45	Intestinal Infection Due to Clostridium Difficile

For peer review only

For peer review only - http://bmjopen.bmj.com/site/about/guidelines.xhtml

008.49	Intestinal Infection Due to Other Organisms

Obstructive Asthma:

493.9	Asthma Unspecified
493.22	Chronic Obstructive Asthma with (Acute) Exacerbation

Bronchitis:

494.1	Bronchiectasis with Acute Exacerbation

For peer review only - http://bmjopen.bmj.com/site/about/guidelines.xhtml

For peer review only

Appendix Materials 2: Sleep Actigraphy Scoring

According to conventional standards, actigraphy data were classified in 1-minute epochs as wake, sleep, or missing. The first classification was performed by the algorithm of the device manufacturer (Actilife software, version 6.13.3, standard settings, Sadeh scoring algorithm). Off-wrist periods were identified by an algorithm developed by Pulsar Informatics Inc. (Philadelphia, PA), then visually checked, integrated with sleep log information provided by interns via the smartphone and, if necessary, corrected by co-investigators from Pulsar who were experienced with actigraphy scoring of large scale clinical trials. The classification into sleep, wake, and unknown followed the set of rules outlined in Appendix Figure 1. Pulsar's visual scoring of sleep was then independently checked by sleep experts from the CCC. In an iterative process, any discrepancies were documented and then corrected by Pulsar, until agreement with the CCC was reached. During this visual scoring process, both Pulsar and sleep experts from the CCC were blinded to study arm (STD or FLEX). Likewise, PVT-B data were inspected by sleep experts from the CCC blinded to arm (Appenfix Figure 2). PVT-B performance was classified into three categories as [1] adherent (i.e., PVT-B data reflected an effort to do the task correctly, and comments left by the subject did not suggest non-adherence), [2] possibly non-adherent (i.e., PVT-B data reflected a consistently poor effort to do the task correctly, but comments left by the subject did not suggest non-adherence), and [3] non-adherent (i.e., PVT-B data reflected a consistently poor effort to do the task correctly, and comments left by the subject did suggest non-adherence, e.g., performing the task while brushing teeth). Comments left by interns were inspected for distractions and non-fatigue related impairment and flagged accordingly. Comments that could have revealed the study arm were blacked out by Pulsar prior to classification by the CCC.

For peer review only - http://bmjopen.bmj.com/site/about/guidelines.xhtml

Appendix Figure 1. Overview of Actigraphy and PVT scoring and review process

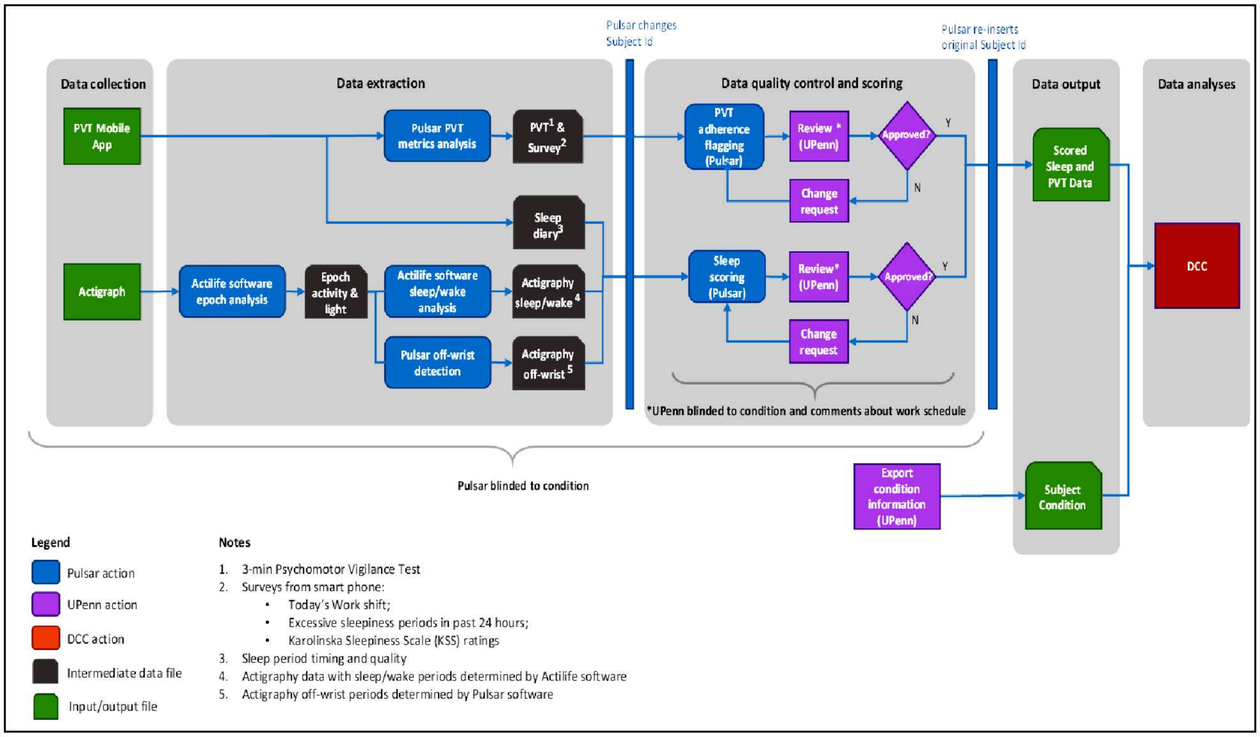

For peer review only - http://bmjopen.bmj.com/site/about/guidelines.xhtml

Appendix Figure 2: Sleep Scoring matrix

	**Sleep Diary had sleep time entered (S)**	**Sleep Diary did not have sleep time entered (W)**
**Actigraphy Reviewed indicated sleep (S)**	Scored as Sleep (S) based on agreement of actigraphy and diary	Scored as Sleep (S) based on actigraphy
**Actigraphy Reviewed indicated wake (W)**	Scored as Wake (W) based on actigraphy	Scored as Wake (W) based on actigraphy
**Actigraphy Reviewed indicated missing or off-wrist (O)**	Scored as Sleep (S) based on diary	Scored as Unknown (U)

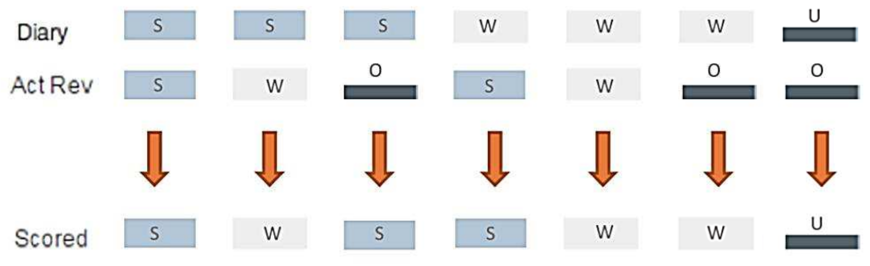

review only

For peer review only - http://bmjopen.bmj.com/site/about/guidelines.xhtml

SPIRIT

STANDARD PROTOCOL ITEMS: RECOMMENDATIONS FOR INTERVENTIONAL TRIALS

SPIRIT 2013 Checklist: Recommended items to address in a clinical trial protocol and related documents*

Section/item	Item No	Description
**Administrative information**		
Title 1	1	Descriptive title identifying the study design, population, interventions, and, if applicable, trial acronym
Trial registration 2,4	2a	Trial identifier and registry name. If not yet registered, name of intended registry
	2b	All items from the World Health Organization Trial Registration Data Set
Protocol version	3	Date and version identifier NA
Funding 2	4	Sources and types of financial, material, and other support
Roles and responsibilities 1	5a	Names, affiliations, and roles of protocol contributors
	5b	Name and contact information for the trial sponsor
*	5c	Role of study sponsor and funders, if any, in study design; collection, management, analysis, and interpretation of data; writing of the report; and the decision to submit the report for publication, including whether they will have ultimate authority over any of these activities
*	5d	Composition, roles, and responsibilities of the coordinating centre, steering committee, endpoint adjudication committee, data management team, and other individuals or groups overseeing the trial, if applicable (see Item 21a for data monitoring committee)
**Introduction**		
Background and rationale 5-6	6a	Description of research question and justification for undertaking the trial, including summary of relevant studies (published and unpublished) examining benefits and harms for each intervention
	6b	Explanation for choice of comparators NA
Objectives 7	7	Specific objectives or hypotheses
Trial design 8,11	8	Description of trial design including type of trial (eg, parallel group, crossover, factorial, single group), allocation ratio, and framework (eg, superiority, equivalence, noninferiority, exploratory)

*In protocol, could be added to summary

For peer review only - http://bmjopen.bmj.com/site/about/guidelines.xhtml

Methods: Participants, interventions, and outcomes

Study setting 8	9	Description of study settings (eg, community clinic, academic hospital) and list of countries where data will be collected. Reference to where list of study sites can be obtained
Eligibility criteria 9-11	10	Inclusion and exclusion criteria for participants. If applicable, eligibility criteria for study centres and individuals who will perform the interventions (eg, surgeons, psychotherapists)
Interventions — Table 2, 30	11a	Interventions for each group with sufficient detail to allow replication, including how and when they will be administered
*	11b	Criteria for discontinuing or modifying allocated interventions for a given trial participant (eg, drug dose change in response to harms, participant request, or improving/worsening disease)
	11c	Strategies to improve adherence to intervention protocols, and any procedures for monitoring adherence (eg, drug tablet return, laboratory tests)
	11d	Relevant concomitant care and interventions that are permitted or prohibited during the trial
Outcomes Table 3, 31 12-17	12	Primary, secondary, and other outcomes, including the specific measurement variable (eg, systolic blood pressure), analysis metric (eg, change from baseline, final value, time to event), method of aggregation (eg, median, proportion), and time point for each outcome. Explanation of the clinical relevance of chosen efficacy and harm outcomes is strongly recommended
Participant timeline 12, Figure 2	13	Time schedule of enrolment, interventions (including any run-ins and washouts), assessments, and visits for participants. A schematic diagram is highly recommended (see Figure)
Sample size 8-9	14	Estimated number of participants needed to achieve study objectives and how it was determined, including clinical and statistical assumptions supporting any sample size calculations
Recruitment 10	15	Strategies for achieving adequate participant enrolment to reach target sample size

Methods: Assignment of interventions (for controlled trials)

Allocation:

Sequence generation 11	16a	Method of generating the allocation sequence (eg, computer-generated random numbers), and list of any factors for stratification. To reduce predictability of a random sequence, details of any planned restriction (eg, blocking) should be provided in a separate document that is unavailable to those who enrol participants or assign interventions

For peer review only - http://bmjopen.bmj.com/site/about/guidelines.xhtml

Allocation concealment mechanism	16b	Mechanism of implementing the allocation sequence (eg, central telephone; sequentially numbered, opaque, sealed envelopes), describing any steps to conceal the sequence until interventions are assigned	
Implementation	16c	Who will generate the allocation sequence, who will enrol participants, and who will assign participants to interventions	
Blinding (masking)	17a	Who will be blinded after assignment to interventions (eg, trial participants, care providers, outcome assessors, data analysts), and how	
	17b	If blinded, circumstances under which unblinding is permissible, and procedure for revealing a participant's allocated intervention during the trial	

Methods: Data collection, management, and analysis

Data collection methods	18a	Plans for assessment and collection of outcome, baseline, and other trial data, including any related processes to promote data quality (eg, duplicate measurements, training of assessors) and a description of study instruments (eg, questionnaires, laboratory tests) along with their reliability and validity, if known. Reference to where data collection forms can be found, if not in the protocol	10, 12-16
	18b	Plans to promote participant retention and complete follow-up, including list of any outcome data to be collected for participants who discontinue or deviate from intervention protocols	NA
Data management	19	Plans for data entry, coding, security, and storage, including any related processes to promote data quality (eg, double data entry; range checks for data values). Reference to where details of data management procedures can be found, if not in the protocol	
Statistical methods	20a	Statistical methods for analysing primary and secondary outcomes. Reference to where other details of the statistical analysis plan can be found, if not in the protocol	17
	20b	Methods for any additional analyses (eg, subgroup and adjusted analyses)	
	20c	Definition of analysis population relating to protocol non-adherence (eg, as randomised analysis), and any statistical methods to handle missing data (eg, multiple imputation)	

Methods: Monitoring

Data monitoring	21a	Composition of data monitoring committee (DMC); summary of its role and reporting structure; statement of whether it is independent from the sponsor and competing interests; and reference to where further details about its charter can be found, if not in the protocol. Alternatively, an explanation of why a DMC is not needed	6-7

	21b		Description of any interim analyses and stopping guidelines, including who will have access to these interim results and make the final decision to terminate the trial
Harms	22	X	Plans for collecting, assessing, reporting, and managing solicited and spontaneously reported adverse events and other unintended effects of trial interventions or trial conduct
Auditing	23		Frequency and procedures for auditing trial conduct, if any, and whether the process will be independent from investigators and the sponsor
**Ethics and dissemination**			
Research ethics approval	24	11	Plans for seeking research ethics committee/institutional review board (REC/IRB) approval
Protocol amendments	25	NA	Plans for communicating important protocol modifications (eg, changes to eligibility criteria, outcomes, analyses) to relevant parties (eg, investigators, REC/IRBs, trial participants, trial registries, journals, regulators)
Consent or assent	26a	X	Who will obtain informed consent or assent from potential trial participants or authorised surrogates, and how (see Item 32)
	26b	X	Additional consent provisions for collection and use of participant data and biological specimens in ancillary studies, if applicable
Confidentiality	27	X	How personal information about potential and enrolled participants will be collected, shared, and maintained in order to protect confidentiality before, during, and after the trial
Declaration of interests	28	X	Financial and other competing interests for principal investigators for the overall trial and each study site
Access to data	29	X	Statement of who will have access to the final trial dataset, and disclosure of contractual agreements that limit such access for investigators
Ancillary and post-trial care	30	X	Provisions, if any, for ancillary and post-trial care, and for compensation to those who suffer harm from trial participation
Dissemination policy	31a	X	Plans for investigators and sponsor to communicate trial results to participants, healthcare professionals, the public, and other relevant groups (eg, via publication, reporting in results databases, or other data sharing arrangements), including any publication restrictions
	31b	X	Authorship eligibility guidelines and any intended use of professional writers
	31c	6	Plans, if any, for granting public access to the full protocol, participant-level dataset, and statistical code

For peer review only - http://bmjopen.bmj.com/site/about/guidelines.xhtml

Appendices

Informed consent materials	32	Model consent form and other related documentation given to participants and authorised surrogates
Biological specimens	33	Plans for collection, laboratory evaluation, and storage of biological specimens for genetic or molecular analysis in the current trial and for future use in ancillary studies, if applicable

*It is strongly recommended that this checklist be read in conjunction with the SPIRIT 2013 Explanation & Elaboration for important clarification on the items. Amendments to the protocol should be tracked and dated. The SPIRIT checklist is copyrighted by the SPIRIT Group under the Creative Commons "Attribution-NonCommercial-NoDerivs 3.0 Unported" license.

BMJ Open

Development of the Individualized Comparative Effectiveness of Models Optimizing Patient Safety and Resident Education (iCOMPARE) Trial: A Protocol Summary of a National Cluster-Randomized Trial of Resident Duty Hour Policies in Internal Medicine

Journal:	*BMJ Open*
Manuscript ID	bmjopen-2018-021711.R1
Article Type:	Protocol
Date Submitted by the Author:	29-May-2018

Complete List of Authors:	Shea, Judy; University of Pennsylvania Perelman School of Medicine, Medicine<br>Silber, JH; The Children's Hospital of Philadelphia, Pediatrics<br>Desai, Sanjay; The Johns Hopkins University, Medicine<br>Dinges, David ; University of Pennsylvania Perelman School of Medicine, Psychiatry<br>Bellini, Lisa; University of Pennsylvania Perelman School of Medicine, Medicine<br>Tonascia, James; John Hopkins , Biostatistics<br>Sternberg, Aice ; Johns Hopkins Hospital and Health System, Epidemiology<br>Small, Dylan; University of Pennsylvania, Wharton Statistics<br>Shade, David ; Johns Hopkins University Bloomberg School of Public Health, Epidemiology<br>Katz, Joel; Brigham and Women's Hospital , Medicine<br>Basner, Mathias; University of Pennsylvania Perelman School of Medicine, Psychiatry<br>Chaiyachati, Krisda; University of Pennsylvania Perelman School of Medicine, Medicine ; Cpl Michael J Crescenz VA Medical Center<br>Even-Shoshan, Orit; Children's Hospital of Philadelphia , Pediatrics<br>Bates, David ; Brigham and Women's Hospital, Medicine<br>Volpp, Kevin ; University of Pennsylvania Perelman School of Medicine, Medical Ethics and Policy; Cpl Michael J Crescenz VA Medical Center<br>Asch, David; University of Pennsylvania Perelman School of Medicine, Medicine ; Cpl Michael J Crescenz VA Medical Center<br>Research Group , iCOMPARE

**Primary Subject Heading**:	Medical education and training
Secondary Subject Heading:	Medical education and training, Evidence based practice, Health services research
Keywords:	resident education, duty hours, resident work hours

For peer review only

Development of the Individualized Comparative Effectiveness of Models Optimizing Patient Safety and Resident Education (iCOMPARE) Trial: A Protocol Summary of a National Cluster-Randomized Trial of Resident Duty Hour Policies in Internal Medicine

Judy A. Shea, PhD,[1] Jeffrey H. Silber, MD, PhD,[2] Sanjay V. Desai, MD,[3] David F. Dinges PhD,[4] Lisa M. Bellini, MD,[1] James Tonascia, PhD,[5] Alice L. Sternberg, ScM,[6] Dylan S. Small, PhD,[7] David M. Shade, JD,[6] Joel T. Katz, MD,[8] Mathias Basner, MD, PhD,[4] Krisda H. Chaiyachati, MD, MPH,[1,10] Orit Even-Shoshan, MS,[2] David W. Bates, MD, MSc,[8] Kevin G. Volpp, MD, PhD,[9,10] David A. Asch, MD, MBA[1,10] and the iCOMPARE Research Group

[1]Department of Medicine, University of Pennsylvania, Philadelphia PA, USA

[2]Department of Pediatrics, Children's Hospital of Philadelphia, Philadelphia PA, USA

[3]Department of Medicine, the Johns Hopkins University, Baltimore MD, USA

[4]Department of Psychiatry, University of Pennsylvania, Philadelphia PA, USA

[5]Department of Biostatistics, the Johns Hopkins University, Baltimore MD, USA

[6]Department of Epidemiology, the Johns Hopkins University, Baltimore MD, USA

[7]Wharton Statistics Department, University of Pennsylvania, Philadelphia PA, USA

[8]Department of Medicine, Brigham and Women's Hospital, Boston, MA, USA

[9]Department of Medical Ethics and Policy, University of Pennsylvania, Philadelphia PA, USA

[10]the Cpl Michael J Crescenz VA Medical Center, Philadelphia PA

For peer review only - http://bmjopen.bmj.com/site/about/guidelines.xhtml

Acknowledgements: The iCOMPARE team extends their appreciation to the participating Program Directors, Jeremy M Asch, BA, Amanda K Bertram, MS, Michele M Carlin BA, Sara C Coats, BS, Adrian J. Ecker, Kelsey A. Gangemi, MPH, Alexander S. Hill, BS, Susan K. Malone, PhD, RN, Alyssa M. Yeager, MD and Pulsar Informatics for their support of the trial. The trial was funded by grants from the National Heart, Lung, and Blood Institute (NHLBI); grant number U01HL125388 to Dr. Asch and grant number U01HL126088 to Dr. Tonascia) and the Accreditation Council for Graduate Medical Education.

Corresponding author:
Judy A. Shea, Ph.D.
1317 Blockley Hall
423 Guardian Drive
Philadelphia, PA 19104-6021
215 573-5111
215 573-8778 (fax)
sheaja@pennmedicine.upenn.edu

For peer review only

This paper is subject to NIH Public Access Policy

Trial Registration: clinicaltrials.gov Identifier: NCT02274818

Running title: iCOMPARE Trial of Resident Duty Hour Policies

Number of references: 49

Number of tables: 3

Number of figures: 2

Number of supplementary appendices: 1

Word Count: 5116

Word count abstract: 274

For peer review only - http://bmjopen.bmj.com/site/about/guidelines.xhtml

Key words: resident education, duty hours, resident work hours,

For peer review only

For peer review only - http://bmjopen.bmj.com/site/about/guidelines.xhtml

Abstract

Introduction: Medical trainees' duty hours have received attention globally; restrictions in Europe, New Zealand and some Canadian provinces are much lower than the 80-hours per week enforced in the United States (U.S.). In the U.S., resident duty hours have been implemented without evidence simultaneously reflecting competing concerns about patient safety and physician education. The objective is to prospectively evaluate the implications of alternative resident duty hour rules for patient safety, trainee education, and intern sleep and alertness.

Methods and Analysis. 63 U.S. internal medicine training programs were randomly assigned 1:1 to the 2011 Accreditation Council for Graduate Medical Education resident duty hour rules or to rules more flexible in intern shift length, and number of hours off between shifts for academic year 2015-2016. The primary outcome is calculated for each program as the difference, 30 day mortality rate among Medicare beneficiaries with any of several pre-specified principal diagnoses in the pre intervention year minus 30-day mortality in the intervention year among Medicare beneficiaries with any of several pre-specified principal diagnoses. Additional safety outcomes include readmission rates, prolonged length of stay, and costs. Measures derived from trainees' and faculty responses to surveys and from time-motion studies of interns compare the educational experiences of residents. Measures derived from wrist actigraphy, subjective ratings and psychomotor vigilance testing compare the sleep and alertness of interns. Differences between duty hour groups in outcomes will be assessed by intention-to-treat analyses.

Ethics and dissemination. The University of Pennsylvania Institutional Review Board (IRB) approved the protocol and served as the IRB of record for 40 programs that agreed to sign an Institutional Affiliation Agreement. Twenty-three programs opted for a local review process.

For peer review only - http://bmjopen.bmj.com/site/about/guidelines.xhtml

Trial Registration: clinicaltrials.gov Identifier: NCT02274818

Article Summary:

Strengths and limitations of this study:

iCOMPARE is the largest randomized trial examining the impact of duty hours in internal medicine training programs.

The aims are broad and include patient safety, trainees sleep and alertness and a host of education outcomes.

The protocol draws on a number of data collection methods including real time direct observation of trainee activities, existing and study administered surveys, robust recording of sleep periods over a 14-day period and patient medical record data.

The lag between the observation period and data release for patient medical record data is substantial.

iCOMPARE promises narrow but substantive evidence to inform resident duty hour standards in internal medicine and signals new interest in methodologically stronger research in medical education.

For peer review only - http://bmjopen.bmj.com/site/about/guidelines.xhtml

Introduction

The long hours worked by resident physicians received some academic attention in the 1970s in the United States (U.S.) [1]. It was not until 1984 when those long hours became publicly linked with concerns about patient safety.[2] Safety concerns resonated with a public who found it self-evident that the often 30-hour shifts of resident physicians would lead to fatigue, and that fatigue would lead to errors that would harm patients. Notably, the possible link between duty hours and patient safety was not just a U.S. concern. Many countries began to limit hours in the 1990s. For example, New Zealand has had a limit of 72 hours a week since 1985. [3] The European Working Time Directive has included doctors in training in their limits of 48 hours per week since 2000 (but Denmark has a normal work week of 37 hours). Australia and many Canadian provinces set limits on some shift lengths given concerns for patient and trainee safety. [4] The U.S. was a little slower to come to the table. Although regulation of duty hours across all specialties occurred first in New York State as a reaction to the Libby Zion case, [2] it was not until 2003 when the Accreditation Council for Graduate Medical Education (ACGME)—the organization that oversees resident education in the U.S.—limited residents to 80 hours of work per week averaged over 4 weeks, and limited the length of individual shifts to 24 hours, with an additional 4 hours to safely transfer care to the next resident. Partly on the basis of an Institute of Medicine report and a trial from Brigham and Women's Hospital,[5,6] those regulations were further tightened for first year residents (interns) in 2011, limiting their maximum shift length to 16 hours. This change prompted a charged debate.[7] Proponents argued that the restrictions did not go far enough. Others argued that the regulations were overly restrictive and inflexible, and harbored increased risk to patients by increasing patient handoffs.[8,9]

Meanwhile, large observational studies, using data from Medicare and the Veterans Administration across millions of hospitalizations, found essentially no difference in important patient outcomes

For peer review only

For peer review only - http://bmjopen.bmj.com/site/about/guidelines.xhtml

following implementation of either the 2003 [10-18] or 2011 duty hour standards.[19] Several studies associated the 2011 standards with less direct patient contact, increased perception of medical errors, increased transitions of care, decreased educational opportunities, and only modestly increased quantities of sleep.[20-22] Others found no changes in trainees' educational test scores.[23] Program directors and trainees expressed concern that the rules reduced training quality and increased rather than decreased medical errors.[24-26] The increasing recognition of the importance of supervision – with separate mandates implemented by the ACGME over the same period – added further uncertainty to the debate. In the end, a well-meaning effort to manage resident fatigue was perceived by many to promote burnout, increase handoffs, decrease educational opportunities and delay the professional maturation required to produce competent, independent physicians. Currently, the evidence available to resolve these controversies is limited to a patchwork of laboratory and in vivo studies of sleep deprivation, large scale epidemiological observations of patient outcomes, surveys of resident and educator opinions, and often single-center trials of unique residency duty hour designs that focused on workload or sleep but not patient outcomes.

In this context, we created the iCOMPARE (individualized Comparative Effectiveness of Models Optimizing Patient Safety and Resident Education) trial, a cluster-randomized trial carried out by internal medicine (IM) residency programs in the U.S. during the 2015-2016 academic year. Participating residency programs were randomized to one of two groups: 1) maintain standard (STD) duty hour rules, or 2) permit a more flexible (FLEX) set of duty hour rules, noted principally for removing the 16-hour shift length restriction for interns and allowing them to work up to 24 hours with an additional 4 hours for care transitions. In contrast to prior work, iCOMPARE was designed to simultaneously assess the impact of duty hour rules on patient safety, resident education, and intern sleep and alertness.

For peer review only - http://bmjopen.bmj.com/site/about/guidelines.xhtml

While the iCOMPARE trial was being planned, leaders in surgical education developed a roughly parallel trial, the Flexibility in Duty Hours Requirements for Surgical Trainees (FIRST) trial, which was fielded in the 2014-2015 academic year. The design of the FIRST trial and its initial results have been published.[27,28] Here we describe the design of the iCOMPARE trial.

Funding and Organization

The iCOMPARE trial is funded primarily by the National Heart, Lung, and Blood Institute (NHLBI), with additional funding from the ACGME. The NHLBI appointed an independent Data and Safety Monitoring Board (DSMB) to advise the Institute regarding the trial's progress, monitor data quality and safeguard the interests of study participants.

Study Aims and Hypotheses

Study hypotheses are presented in Table 1. The primary hypothesis for the trial is that 30-day any-site patient mortality under FLEX will not exceed (will not be inferior to) 30-day patient mortality under STD, measured as the difference in difference across STD and FLEX programs between a program's 30-day patient mortality rate in the trial year and that rate in the pre-trial year (i.e., 30-day patient mortality rate at a program during the trial year minus 30-day patient mortality rate at the program in the pre-trial year). Secondary outcomes related to patient safety include 7-day and 30-day hospital readmission rates, complication rates, the probability of a prolonged length of stay, total resource utilization, and Medicare payments.

ICOMPARE's education hypotheses are that trainees in the FLEX arm will spend more time in direct patient care and education and have greater satisfaction with educational experiences compared to their STD arm peers; that standardized test scores for interns in FLEX will not be lower than such scores for interns in the STD arm; that faculty in the FLEX arm will report greater satisfaction with their clinical

For peer review only - http://bmjopen.bmj.com/site/about/guidelines.xhtml

teaching experiences and greater perceptions of safety, teamwork and supervision than faculty in the STD arm.

ICOMPARE's sleep hypotheses are that the average daily sleep will not be less among interns in the FLEX arm compared to those in the STD arm, and that interns in the FLEX arm will not have greater subjective sleepiness or lower behavioral alertness than interns in the STD arm.

Methods and Analysis

Study Design

iCOMPARE is a cluster-randomized trial to compare two alternative duty hour standards in 63 IM training programs in the United States fielded in academic year 2015-2016. Table 2 compares the duty hour standards between the FLEX and STD arms.

Cluster randomization occurred at the level of the residency program, the level at which duty hour policies are implemented. Although duty hour standards are mandated, individual programs vary considerably in how they schedule their trainees within those standards. The trial is pragmatic in that the intervention arm effectively represents the exposure of residency programs to an alternative set of duty hour standards. The exposure is the policy change, not the actual duty hours that are implemented in response. This approach is akin to clinical trials of outpatient pharmaceuticals in which the exposure is the prescription of the control or intervention drug, regardless of participant adherence.

Hypotheses regarding how interns spend their time require detailed time-motion observations, and hypotheses about interns' sleep and alertness require detailed observations of sleep cycles and psychomotor vigilance—both accomplished through sub-studies deployed in a sample of participating programs.

Sample Size and Power

For peer review only - http://bmjopen.bmj.com/site/about/guidelines.xhtml

For peer review only

The non-inferiority 30-day patient mortality hypothesis (H1a) is the trial's primary hypothesis. Defining the outcome as the trial year rate minus the pre-trial year rate adjusts each program's outcome for secular trends in 30-day mortality, as well as for differential program-to-program patient risk profiles. Using a two-sample t-test for non-inferiority of the between group mean year over year difference in 30-day mortality and assuming 80% power, Type I error of 5%, a non-inferiority margin of 1%, pooled SD for the outcome of 1.5%, and a 30-day mortality rate of 11%, we calculated a required sample of 58 programs, 29 per treatment group. The pooled SD of the outcome (year over year difference in 30-day mortality) and the 30-day mortality rate in the STD group were estimated using available Medicare claims from 2007-2008 for the population of target IM programs (i.e., all IM Programs meeting the trial's size and population criteria).

This number of programs and the associated trainees and faculty provide excellent power for analyses investigating the education hypotheses which use person-level outcomes. Assuming 30 interns, 50 second year or higher trainees, 10 faculty members, and 1 director per program, iCOMPARE was projected to include 1740 interns, 2900 second year or higher trainees, 580 faculty members, and 58 program directors.

Hypothesis H2a addresses the Flex versus STD difference in percent of time spent by interns in direct patient care and was assessed with a Time-Motion study. The sample size of the Time-Motion study is 6 programs (3 FLEX, 3 STD; 60 interns, 10 interns per program). Preliminary data [29,30] suggested that the mean percent time spent in direct patient care in STD programs would be 13% ± 4%. The Time-Motion study has at least 80% power to detect a 3% difference between FLEX and STD in time spent in direct care.

For peer review only - http://bmjopen.bmj.com/site/about/guidelines.xhtml

Hypothesis H3a addresses the FLEX versus STD difference in average intern daily sleep over a 14-day period. Targeting 90% power, one-sided Type I error of 5%, a non-inferiority margin of 0.5 hours, and assuming expected average daily sleep in STD of 6.9 ± 1.5 hours [31], the sample size for the Sleep and Alertness study was calculated to be 290 interns (145 per treatment group) and increased to 384 interns (192 per treatment group) to anticipate data loss related to non-adherence and dropouts.

Study Population and Inclusion Criteria

The CONSORT diagram is provided in Figure 1. In 2014 there were 379 IM ACGME-accredited training programs in the country not on probation. The 54 programs in New York State were excluded because that state's legislated duty hour standards were not subject to the waiver required for the intervention arm. Since the patient safety outcomes would be measured in Medicare patients, Veterans Administration hospitals did not qualify for inclusion. Sufficiently precise estimates of our patient safety outcome measures necessitated exclusion of 84 programs training only at hospitals in the bottom 50% by trainee-to-bed ratio or the bottom 25% by patient volume in the measured diagnoses. In about 50% of teaching hospitals, the number of residents per bed is so few that the residents' impact on patient care is minimal, and so any changes in the impact of those residents' schedules would be too insignificant to measure.

An additional 62 programs in the lowest quartile of number of trainees were excluded to ensure measurement of enough trainees. Two other programs in the lowest quartile of number of trainees were allowed by the Steering Committee. One program was approved for inclusion despite deviations in size of its two affiliated hospitals because together the two hospitals met the qualifying hospital size. A second program was approved for inclusion because the hospital it was affiliated with had far exceeded the trainee-to-bed ratio even though the patient volume was slightly low. A total of 179 programs were eligible for inclusion.

For peer review only - http://bmjopen.bmj.com/site/about/guidelines.xhtml

Recruitment

Directors of eligible programs were invited to participate by letters of invitation and informational sessions during the Fall 2014 and Spring 2015 Association of Program Directors in Internal Medicine (APDIM) meetings. Participation required agreement to support study needs and a participation agreement signed by the Designated Institutional Official indicating approval from the institution's Graduate Medical Education Committee. Program directors indicated the hospitals, and the trainee rotations within those hospitals, which would participate in iCOMPARE. Each program was reviewed by the study team to confirm that at least one of the hospitals indicated by the program director qualified for inclusion in the trial. The study team checked the list of hospitals provided by the program director for shared staffing with other training programs eligible for and interested in iCOMPARE so that those programs could be randomized together and avoid having different duty hour schedules for trainees at a common hospital. This occurred for 3 pairs of hospitals. In total, 63 programs agreed to participate and were randomized.

Patient and Public Involvement

The study was based on a fundamental concern about the impact of training schedules on patient, trainee and program director experiences. However, no patients were directly involved in the design and conduct of the study. Program directors from each participating program were involved and essential to recruitment which was at the training program level. Program directors were fully informed about the intervention and individual trainees had the opportunity to decline participation in surveys and other education outcomes. Results will be presented at national meetings to program directors and published in peer-reviewed journals.

For peer review only - http://bmjopen.bmj.com/site/about/guidelines.xhtml

Institutional Review Board Determinations

The University of Pennsylvania Institutional Review Board (IRB) approved the iCOMPARE protocol and served as the IRB of record for all participating programs that agreed to sign an Institutional Affiliation Agreement. Forty programs chose this option and submitted the required paperwork. Twenty-three programs opted for a local review process.

Randomization

Randomization was 1:1 into the STD and FLEX arms. Participating programs in iCOMPARE were not masked to their assignment. Sixty programs completed the application process quickly and were randomized in November 2014 so that they could provide applicants interviewing for positions in academic year 2015-2016 information about the duty hour arm assignment. Randomization of programs ended in March 2015, judged to be the latest date by which a program could be randomized to the FLEX arm and have adequate time to adjust their residency schedules in anticipation of the 2015-2016 intervention year.

Data Sources

Hypothesis testing required outcome data from patients, trainees, and program directors and faculty. Because of the available and highly reliable patient mortality information (both in hospital and after discharge) in the Medicare fee for service (FFS) program, the iCOMPARE patient population was limited to Medicare FFS beneficiaries with a qualifying principal diagnosis on hospital admission (Supplementary Appendix: Appendix Materials 1); these diagnoses were chosen for their common treatment on internal medicine services (excluding oncology and neurology diagnoses) and their elevated mortality rates. Because similar data are not available for patients enrolled in a Medicare managed care program,

For peer review only - http://bmjopen.bmj.com/site/about/guidelines.xhtml

patients enrolled in a Medicare managed care program six months before or one month post discharge for the index hospital admission were excluded.

Randomized programs were further invited to participate in the observational Time-Motion and the Sleep and Alertness sub-studies, described below.

Timeline

Trial development began in 2013. The Medicare data required for the analyses of the patient safety outcomes are not expected to be available until mid 2018 (Figure 2).

Outcomes, Measures, and Data Collection

Aim 1 – Patient Safety

Patient safety measures are derived from Medicare data, collected as part of routine clinical practice and administration and obtained through ResDAC (https://www.resdac.org/). The primary outcome is based on 30-day (from admission) all-location mortality, a patient-centered measure unaffected by length of hospitalization.[10,11,15,18,19,32,33] Secondary outcomes include prolonged length of stay,[14,34-36] 30-day readmission rate, [19,37] complication rate,[13,34,38,39] and resource utilization measures (cost) [17,35] and payment among fee-for-service Medicare beneficiaries with specific diagnoses. Details regarding justification for and operationalization of these outcomes are provided in Supplementary Appendix: Appendix Materials 2.

Aim 2 – Education

Education measures are derived from multiple sources. The primary education measures are specified in Table 3 and come from the Time-Motion sub-study, the ACGME year-end trainee and core faculty surveys, and the interns' In-Training Examination (ITE) scores provided by the American College of

For peer review only - http://bmjopen.bmj.com/site/about/guidelines.xhtml

Physicians (ACP). Secondary education measures include iCOMPARE-generated surveys and survey data provided by the Association of Program Directors in Internal Medicine (APDIM).

Time-Motion sub-study. We recruited 3 IM programs randomized to STD and 3 randomized to FLEX to participate in direct observations of some of their interns, targeting programs in the mid-Atlantic region for operational convenience; programs with tertiary hospitals as well as community based programs were included in both arms. Participating programs received $3,000 to support these sub-study activities. We recruited interns rotating on General Medicine services between March-May 2016. Eligible and interested interns provided written consent. Among the 129 interns invited to participate, 120 (93%) consented.

Twenty-three observers (medical students and undergraduates) were trained to follow participating interns. They used a custom-built tablet-based software program to document start and stop times for various intern activities: direct patient care, indirect patient care, education, rounds, work, handoffs, and miscellaneous, each with various subcategories reflecting greater specificity of tasks. For example, direct patient care had subcategories for patient interactions, family interactions, and physical contact (e.g., physical exam). At least one activity had to be selected at all times, although more than one could be selected to reflect multitasking. At the start and stop of the shift an observer completed brief surveys that summarized total patient census numbers for that intern, including the numbers of transfers, discharges, admissions, and patients received at the beginning of a shift and handed-off at the end.

Shifts were selected at each site aiming to capture 30 shifts in a proportion mirroring – specific for each site – how interns generally spend their time on a general medicine inpatient rotation in a given week. Observers shadowed approximately 8-10 shifts, for 1-3 shifts per intern. Extended shifts (e.g., 24 hour call cycles) were often split by two observers. A 10% sample of shifts was observed simultaneously by two observers to estimate inter-rater reliability. The methods for the Time-Motion sub-study were

For peer review only - http://bmjopen.bmj.com/site/about/guidelines.xhtml

based on previous research in terms of the structure of observations (e.g. categories used), process of observations (e.g. platform used and scheduling strategies), and the training of observers [27,28]. Inter-rater reliability was high for pairs of observers using a training video (median kappa = .67 and median agreement of 90%) as well as the real-time observations (median kappa of .74 and median agreement of 89%). Interns received a $50 Amazon gift card for each observed shift.

ACGME end-of year-survey data. The ACGME provided iCOMPARE with summary data derived from their end-of-year surveys of trainees and core faculty in May 2014, May 2015, and May 2016; all data provided by the ACGME to iCOMPARE was at the level of treatment group arm (FLEX or STD) and year collected (2014, 2015, 2016). All data were stripped of program identifiers and individual respondent identifiers. iCOMPARE received summary treatment-group level information for the single question items specified in hypotheses H2b and H2c and many content areas (combined responses over several questions covering related content) that comprise secondary education outcomes.

ITE score data from ACP. The ACP provided iCOMPARE with trainee level ITE scores from 2015 and 2016 for those trainees who had given permission to share their score for research purposes. Identifying information for a score was limited to PGY year and program identifier.

APDIM annual survey data. The APDIM provided data from its survey of program directors in fall 2015 and fall 2016. The deidentified data set did not include name of program or program director but did have a flag for STD or FLEX or not in study.

End-of-Shift Surveys. From August 31, 2015 to April 26, 2016, iCOMPARE conducted 16 two-week cycles of daily surveys administered to all trainees at all participating programs. At the start of each cycle, we randomized each trainee into one of 14 groups and then allocated each group to a day of the 2-week period to receive a survey. Thus, each trainee was surveyed once every two weeks.

For peer review only - http://bmjopen.bmj.com/site/about/guidelines.xhtml

Surveys alternated between two sets of questions. Survey 1 was sent every other day and asked: [1] the name of the rotation a trainee was on in the past 24 hours, specifying details such as inpatient or not, type of inpatient rotation, at main teaching hospital or other setting, or not in hospital; and if the trainee was on an inpatient rotation; [2] number of new patient evaluations completed in past 24 hours; [3] number of handoffs experienced in past 24 hours; and [4] the number of patients for which the trainee was the primary provider. These questions provide another view of how interns spend their time.

Survey 2 was sent on the alternate days and asked the same first question as Survey 1, as well as the trainee's ratings (too little, just right, too much) for [1] time spent in educational conference and related activities, [2] sense of ownership of patients, [3] work intensity, and [4] continuity of care. Data for these questions are related to satisfaction and complement the iCOMPARE's end-of-year survey (described below) and ACGME survey (described above).

Trainees were entered into an incentive lottery designed so that in each 2-week cycle, one intern and one resident at each of the 63 IM programs were each awarded either a $25 or $100 Amazon gift card if they had completed their survey during that period. After the first cycle, the cycle response rate ranged between 39% and 42%.

iCOMPARE end-of-year surveys. All trainees and program directors received an iCOMPARE study-specific survey in May-June 2015 (baseline) and again in May-June 2016 (post-intervention), administered via Lime Survey (https://www.limesurvey.org/). Invitations with personalized links were emailed during May of 2015 and May of 2016 with up to 6 reminders to nonrespondents. At the end of the intervention year, a $2,500 cash incentive was provided to each of the 9 programs with the highest response rates.

The trainee survey was administered to all trainees with only slight differences between versions for interns and PGY2 and higher trainees. The instrument was initially developed for the FIRST trial[26] and included items on trainee satisfaction, experience of duty hours, supervision, fatigue management, and

For peer review only - http://bmjopen.bmj.com/site/about/guidelines.xhtml

resident and patient safety, and ended with the Maslach Burnout Inventory-Human Services Survey (MBI-HSS).[40] The MBI-HSS is a 22-item rating scale assessing three domains: emotional exhaustion (9 items), depersonalization (5 items), and lack of personal accomplishment (8 items). Items are answered on a frequency scale of 0 to 6 where 0 indicates never and 6 indicates every day.

The program director survey was modeled from an earlier survey to program directors [41] and included items on resident and faculty workload, resident morale, continuity, education, patient safety, and program finances and administration.

Aim 3 – Sleep and alertness

Outcomes for the third aim include sleep duration and both subjective and objective measures of alertness among interns at 6 sites randomized to STD and 6 sites randomized to FLEX. At each of these 12 sites, program coordinators recruited interns scheduled to be on general medicine, medical intensive care, cardiology, or cardiac care rotations. Each participating program received $8,000 to cover the costs associated with scheduling interns for data acquisition and managing study equipment. Data collection spanned November 5, 2015 to May 31, 2016. The 12 programs in the Sleep and Alertness Sub-study were selected from across the country and represented programs of various sizes. 457 interns at these 12 programs participated in the consent process; 432 (94.5%) provided written consent, 12 of whom were not eligible due to rotation schedules. Of the remaining 420, 398 (94.8%) provided data for the study and 22 (5.3%) discontinued participation

Participating interns underwent 14-days of continuous measurements of rest and activity via wrist actigraphs (model wGT3X-BT, The Actigraph Corp., Pensacola, FL).[42-44] Interns were instructed to wear the actigraph continuously, even on days off, except during activities that might damage it (e.g., water immersion or contact sports) or that would impede the delivery of clinical care. They were asked

For peer review only - http://bmjopen.bmj.com/site/about/guidelines.xhtml

to remove the actigraph for up to 2 waking hours for recharging on days 1 and 7. Each participating intern received a $10 Amazon gift card for each day for which data were received.

Each morning, the intern completed a brief online survey including the name of the shift that the intern was working; a log reflecting sleep times, sleep quality, and experiences of excessive sleepiness; and the Karolinska Sleepiness Scale.[45] The interns then completed a 3-minute Psychomotor Vigilance Test (PVT-B) [46,47]. All surveys and assessments were completed on a study-supplied Android Smartphone (Samsung Galaxy SIII Neo). The PVT-B is based on simple reaction time to stimuli occurring at random inter-stimulus intervals and is the gold standard for measuring the neurobehavioral effects of acute and chronic sleep loss and circadian misalignment.[48] Actigraphy, survey, and PVT-B data were automatically encrypted and uploaded to a secure server and checked daily for protocol adherence and potential technical issues with the equipment. If problems were detected or no data were received, interns were contacted by the study team to resolve any difficulties interns were having with equipment. Details of scoring are presented in the Supplementary Appendix: Appendix Materials 3: Sleep Actigraphy Scoring and Supplementary Appendix Figure 1 and Figure 2.

Statistical Considerations

Non-inferiority tests will be one-sided and superiority tests will be two-sided. All primary analyses will compare the FLEX and STD treatment groups as randomized, regardless of adherence to the assigned duty hour standards, according to the intention-to-treat principle. Since directors at programs assigned to FLEX have considerable latitude in design of trainee schedules, we expect variation amongst the duty-hour schedules followed in the FLEX group. Protocol-specified secondary analyses addressing the degree of difference between FLEX and STD schedules will be completed. We will also report mortality results adjusting for the clinical condition associated with patient's principal diagnosis as well as demographic

For peer review only - http://bmjopen.bmj.com/site/about/guidelines.xhtml

For peer review only

variables and comorbidities determined using a 6-month look-back period. Similar approaches will be used for the other patient safety outcomes specified in hypotheses H1b-H1e.

The outcome measures for the education hypotheses (H2a-H2d) and the sleep and alertness hypotheses (H3a-H3b) are person-level (intern, trainee, or faculty) measures. If we have repeated measures of an outcome on an individual (e.g., an intern has observations of time spent in direct patient care over multiple shifts, an intern has observations of time spend sleeping over more than 1 day) we will analyze the mean of the repeated measures on the individual, which eliminates within-person correlations. Each outcome will be the response variable in a mixed effects linear or logistic regression model with a random intercept for each program cluster and a single fixed term for treatment group as implemented in SAS (Cary, NC) or STATA (College Station, TX). When pre-trial year data are available, additional analyses will adjust an individual's trial year mean response for the pre-trial year program-level mean response (i.e., a difference of differences analysis). The pre-trial program level mean response must be used since the trainees and faculty providing data in the pre-trial year are not the same trainees and faculty providing data in the trial year, but are at the same IM program. Specifically we have pre-trial data for Medicare patient level safety analyses, and for some Education outcomes (the ACGME end-of-year surveys, ITE score data from ACP, and the iCOMPARE end-of-year surveys).

Dissemination

Plans for dissemination include submitting results for the various aims to academic meetings and peer-reviewed publications as they become available. The Data and Safety Monitoring Board will comment on manuscripts reporting results related to the primary hypotheses before journal submission.

Discussion

For peer review only - http://bmjopen.bmj.com/site/about/guidelines.xhtml

iCOMPARE is a one-year, cluster-randomized, pragmatic trial designed to evaluate the availability of an alternative resident duty hour schedule for effects on patient safety, resident education, and intern sleep and alertness. iCOMPARE's design is similar to that of the FIRST trial conducted among surgical trainees. What distinguishes both of these trials from prior work investigating effects of duty hour schedules, in addition to their large size, comprehensive approach, and immediate policy relevance, is that they help elevate the standards of evidence applied to graduate medical education policy more generally in the United States and abroad. Although randomized trials provide strong evidence for the causal inference required for good policy, they typically answer narrow questions. Those questions today revolve around the length of shifts within an 80-hour work week. Inside the field of graduate medical education, these issues have been hotly debated, albeit the 80-hours in the U.S. far exceed the limits set for other counties with similar educational systems .[3.4]

iCOMPARE has some limitations. The measurement of patient safety relies predominantly on mortality effects, an important but incomplete measure of relevant clinical outcomes. The measurement of education relies considerably on existing and conventional tests and surveys, and those developed specifically for this study, whereas education is more dimensionalized and, indeed, its own value is perhaps better reflected in changes in how well physicians perform through their careers, unmeasured in this study. Nevertheless, this pragmatic study of health care policy targets measures that are likely to be influential in policy decisions about resident duty hours and available in a time frame when the answers to those questions remain relevant.

Some may wonder why duty hours or shift lengths were not imposed on the intervention group and why, instead, the intervention group merely had permission to use more flexibility in their scheduling. The design of this study means that the potency of the duty hour changes actually implemented by programs may be less extreme than what was permitted by the intervention, adding noise to or

For peer review only - http://bmjopen.bmj.com/site/about/guidelines.xhtml

potentially weakening the overall observed effect. Those concerns are valid to the extent the question is: What happens to patient safety, education, or sleep and alertness when residents are forced to work in certain shifts? But the question at hand is: What happens when programs are allowed flexibility in their scheduling of shifts? The outcome of those policy decisions is, in its implemented state, a product of the flexibility of the rules and the degree to which individual programs take advantage of that flexibility. In some ways, this study design is consistent with that of effectiveness trials of drugs, where the anticipated effect is a product not just of whether one was randomized to the study drug, but also whether one was adherent to the drug. In the real world, adherence is relevant. Context is similarly relevant. The effects we observe in this trial also depend critically on the oversight and supervision provided to interns by more senior residents and on other safety nets built into the environment of hospital practice. While those safety nets potentially blunt observed effects, they take this study beyond the in vitro relevance of laboratory study to a pragmatic context.

With the results of the FIRST trial demonstrating non-inferiority in patient outcomes when a more flexible schedule was available, the ACGME issued new duty hour standards, effective July 2018, that correspond to the intervention arm of iCOMPARE.[49]

Given that surgery and internal medicine are large fields with many residents caring for many patients, it is important to study the duty hour rules in both specialties as surgical and medical training programs differ in structure, process, culture, the kinds of residents they attract, the patients they serve, and the duties of trainees.

Conclusions

There is considerable interest in understanding concerns in the US health care system, such as why US health care is so expensive, why its overall effects on health lag behind those of peer nations in particular with respect to safety, and why its individual effects are so unevenly distributed. In contrast,

For peer review only - http://bmjopen.bmj.com/site/about/guidelines.xhtml

relatively little effort has been devoted to studying how physicians are trained—surprising because although physicians are certainly not the only reason for the concerns, it seems evident that physician training must play an important role in most outcomes related to US health care. In that broad context, iCOMPARE promises narrow but substantive evidence to inform resident duty hour standards in internal medicine and signals new interest in methodologically stronger research in medical education.

For peer review only

For peer review only - http://bmjopen.bmj.com/site/about/guidelines.xhtml

References

1. Friedman RC, Bigger JT, Kornfeld DS. The intern and sleep loss. *N Engl J Med* 1971;285(4):201–3.
2. Asch DA, Parker RM. The Libby Zion case: One step forward or two steps backward? *N Engl J Med* 1988;318:771-5.
3. Resident Duty Hours: Enhancing Sleep, Supervision and Safety, Appendix C: International Experiences Limiting Duty Hours. Pp 339-361, National Academies Press: Washington, DC. 2009
4. Temple J. Resident duty hours around the globe: Where are we now? *BMC Med Educ* 2014;14(u 1):S8
5. Ulmer C, Wolman DM, Johns MME, eds. Resident Duty Hours: Enhancing Sleep, Supervision, and Safety. National Academies Press: Washington, DC. 2009.
6. Landrigan CP, Rothschild JM, Cronin JW, et al. Effects of reducing interns' work hours on serious medical errors in intensive care units. *N Engl J Med* 2004;351(18):1838-48.
7. Asch DA, Bilimoria KY, Desai SV. Resident duty hours and medical education policy — raising the evidence bar. *N Engl J Med* 2017;376:1704-6.
8. Petersen LA, Brennan TA, O'Neil AC, Cook EF, Lee TH. Does housestaff discontinuity of care increase the risk for preventable adverse events? *Ann Intern Med* 1994;121(11):866-72.
9. Kitch BT, Cooper JB, Zapol WM, et al. Handoffs causing patient harm: a survey of medical and surgical house staff. *Jt Comm J Qual Patient Saf* 2008;34(10):563-70.
10. Volpp KG, Rosen AK, Rosenbaum PR, et al. Mortality among hospitalized Medicare beneficiaries in the first 2 years following ACGME resident duty hour reform. *JAMA* 2007;298:975-83.
11. Volpp KG, Rosen AK, Rosenbaum PR, et al. Mortality among patients in VA hospitals in the first 2 years following ACGME resident duty hour reform. *JAMA* 2007;298:984-92.
12. Prasad M, Iwashyna TJ, Christie JD, et al. Effect of work-hours regulations on intensive care unit

For peer review only - http://bmjopen.bmj.com/site/about/guidelines.xhtml

mortality in United States teaching hospitals. *Crit Care Med* 2009; 37:2564-69.

13. Rosen AK, Loveland SA, Romano PS, et al. Effects of resident duty hour reform on surgical and procedural patient safety indicators among hospitalized Veterans Health Administration and Medicare patients. *Med Care* 2009; 47:723-31.

14. Silber JH, Rosenbaum PR, Rosen AK, et al. Prolonged hospital stay and the resident duty hour rules of 2003. *Med Care* 2009; 47:1191-200.

15. Volpp KG, Rosen AK, Rosenbaum PR, et al. Did duty hour reform lead to better outcomes among the highest risk patients? *J Gen Intern Med* 2009; 24:1149-55.

16. Press MJ, Silber JH, Rosen AK, et al. The impact of resident duty hour reform on hospital readmission rates among Medicare beneficiaries. *J Gen Intern Med* 2011; 26:405-11.

17. Navathe AS, Silber JH, Small DS, et al. Teaching hospital financial status and patient outcomes following ACGME duty hour reform. *Health Serv Res* 2013; 48:476-98.

18. Volpp KG, Small DS, Romano PS, et al. Teaching hospital five-year mortality trends in the wake of duty hour reforms. *J Gen Intern Med* 2013; 28:1048-55.

19. Patel MS, Volpp KG, Small DS, et al. Association of the 2011 ACGME resident duty hour reforms with mortality and readmissions among hospitalized Medicare patients. *JAMA* 2014; 312:2364-73.

20. Block L, Habicht R, Wu AW, et al. In the wake of the 2003 and 2011 duty hours regulations, how do internal medicine interns spend their time? *J Gen Intern Med* 2013;28(8):1042-47.

21. Desai SV, Feldman L, Brown L, et al. Effect of the 2011 vs 2003 duty hour regulation-compliant models on sleep duration, trainee education, and continuity of patient care among internal medicine house staff: a randomized trial. *JAMA Intern Med* 2013;173(8):649-55.

22. Sen S, Kranzler HR, Didwania AK, et al. Effects of the 2011 duty hour reforms on interns and their patients: a prospective longitudinal cohort study. *JAMA Intern Med* 2013;173(8):657-63.

For peer review only - http://bmjopen.bmj.com/site/about/guidelines.xhtml

For peer review only

23. Silber JH, Romano PS, Itani KM, et al. Assessing the effects of the 2003 resident duty hours reform on internal medicine board scores. *Acad Med* 2014; 89:644-51.

24. Drolet BC, Christopher DA, Fischer SA. Residents' response to duty-hour regulations--a follow-up national survey. *N Engl J Med* 2012;366(24):e35.

25. Drolet BC, Khokhar MT, Fischer SA. The 2011 duty-hour requirements--a survey of residency program directors. *N Engl J Med* 2013;368(8):694-97.

26. Drolet BC, Spalluto LB, Fischer SA. Residents' perspectives on ACGME regulation of supervision and duty hours--a national survey. *N Engl J Med* 2010;363(23):e34.

27. Bilimoria KY, Chung JW, Hedges LV, et al. Development of the flexibility in duty hour requirements for surgical trainees (FIRST) trial protocol: A national cluster-randomized trial of resident duty hour policies. *JAMA Surg* 2016;151:273-81.

28. Bilimoria KY, Chung JW, Hedges LV, et al. National cluster-randomized trial of duty-hour flexibility in surgical training. *N Engl J Med* 2016; 374:713-27.

29. Fletcher KE, Visotcky AM, Slagle JM, et al. The composition of intern work while on call. *J Gen Intern Med* 2012;27(11):1432-7.

30. Block L, Habicht R, Wu AW, et al. In the wake of the 2003 and 2011 duty hours regulations, how do internal medicine interns spend their time? *J Gen Intern Med* 2013;28:1042-7.

31. Volpp KG, Shea JA, Small DS, et al. Effect of a protected sleep period on hours slept during extended overnight in-hospital duty hours among medical interns. *JAMA* 2012;308(21):2208-17.

32. Drye E, Normand SL, Wang Y, et al. Comparison of hospital risk-standardized mortality rates calculated by using in-hospital and 30-day models: implications for hospital profiling. *Ann Intern Med* 2012;156:19-26.

33. Meehan TP, Fine MJ, Krumholz HM, et al. Quality of care, process, and outcomes in elderly patients with pneumonia. *JAMA* 1997;278:2080-4.

For peer review only

For peer review only - http://bmjopen.bmj.com/site/about/guidelines.xhtml

34. Silber JH, Rosenbaum PR, Even-Shoshan O, et al. Length of stay, conditional length of stay, and prolonged stay in pediatric asthma. *Health Serv Res* 2003;38:867-86.

35. Silber JH, Rosenbaum PR, Kelz RR, et al. Medical and financial risks associated with surgery in the elderly obese. *Ann Surg* 2012;256:79-86.

36. Silber JH, Rosenbaum PR, Koziol LF, Sutaria N, Marsh RR, Even-Shoshan O. Conditional length of stay. *Health Serv Res* 1999;34:349-63.

37. Krumholz HM, Lin Z, Keenan PS, et al. Relationship between hospital readmission and mortality rates for patients hospitalized with acute myocardial infarction, heart failure, or pneumonia. *JAMA* 2013;309:587-93.

38. Iezzoni LI, Daley J, Heeren T, et al. Using administrative data to screen hospitals for high complication rates. *Inquiry* 1994;31:40-55.

39. McCarthy EP, Iezzoni LI, Davis RB, et al. Does clinical evidence support ICD-9-CM diagnosis coding of complications? *Med Care* 2000;38:868-76.

40. Maslach C, Jackson SE, Leiter MP. Maslach Burnout Inventory. 3rd Ed. Palo Alto: Consulting Psychologists Press. 1996.

41. Shea JA, Willett LL, Borman KR, et al. Anticipated consequences of the 2011 duty hour standards: A view from surgery and medicine program directors. *Acad Med* 2012:87;895-903.

42. Ancoli-Israel S, Cole R, Alessi C, et al. The role of actigraphy in the study of sleep and circadian rhythms. *Sleep* 2003;26(3):342-92

43. Malmberg B, Kecklund G, Karlson B, et al. Sleep and recovery in physicians on night call: a longitudinal field study. *BMC Health Serv Res* 2010;10(239).

44. Basner M, Dinges DF, Shea JA, et al. Sleep and Alertness in Medical Interns and Residents: An observational study on the role of extended shifts. *Sleep* 2017;40(4). doi: 10.1093/sleep/zsx027

For peer review only - http://bmjopen.bmj.com/site/about/guidelines.xhtml

45. Akerstedt T, Gillberg M. Subjective and objective sleepiness in the active individual. *Int J Neurosci* 1990;52(1-2):29-37.

46. Basner M, Mollicone DJ, Dinges DF. Validity and sensitivity of a brief Psychomotor Vigilance Test (PVT-B) to total and partial sleep deprivation. *Acta Astronautica* 2011;69:949-59.

47. Basner M, Dinges DF. Maximizing sensitivity of the Psychomotor Vigilance Test (PVT) to sleep loss. *Sleep* 2011;34(5):581-91.

48. Dinges DF, Maislin G, Hanowski RJ, et al. Commercial Motor Vehicle (CMV) Driver Restart Study: Final Report. U.S. DoT; Federal Motor Carrier Safety Administration; Office of Analysis, Research, and Technology. 2016.

49. The ACGME Common Program Requirements Section VI revisions from TJ Nasca, MD. ACGME, 2017. (Accessed August 15, 2017, at http://www.acgme.org/Portals/0/PDFs/Nasca-Community/Section-VI-Memo-3-10-17.pdf.)

For peer review only

For peer review only - http://bmjopen.bmj.com/site/about/guidelines.xhtml

Authors' contributions

Judy A. Shea, PhD is a co-investigator. She took the lead in drafting this paper and incorporating all co-authors' suggestions on subsequent drafts. She also worked closely with Drs. Desai and Bellini to design and refine the aims and methods related to the education aim.

Jeffrey H. Silber, MD, PHD took the lead on writing the protocol for the patient safety component of the protocol. He reviewed and commented on multiple versions of this manuscript.

Sanjay V. Desai, MD worked with Drs. Shea and Bellini to develop the details for the education aim and outcomes. He reviewed and commented on multiple versions of this manuscript.

David F Dinges, PhD, took the lead and worked with Dr. Basner to develop the details for the sleep and alertness aim and outcomes. He reviewed and commented on multiple versions of this manuscript.

Lisa M.Bellini, MD worked with Dr. Shea and Desai to develop the details for the education aim and outcomes. She reviewed and commented on multiple versions of this manuscript.

James Tonascia, PhD took the lead and worked with Alice L Sternberg, ScM, Dylan S. Small, PhD and David M Shade, JD to develop the sample size calculations and analytic plan for all study aims. They each reviewed and commented on multiple versions of this manuscript.

Joel T. Katz, MD participated in design of the direct observation portion of the protocol and commented on multiple versions of the manuscript.

Mathias Basner, MD, PhD worked with Dr. Dinges to develop the details for the sleep and alertness aim and outcomes. He reviewed and commented on multiple versions of this manuscript.

For peer review only - http://bmjopen.bmj.com/site/about/guidelines.xhtml

Krisda H. Chaiyachati, MD, MPH took the lead in developing the details for the direct observation portion of the protocol. He reviewed and commented on multiple versions of this manuscript.

Orit Even-Shoshan, MS worked with Dr. Silber to design the patient safety portion of the protocol.

David W. Bates, MD participated in design of the direct observation portion of the protocol and commented on multiple versions of the manuscript.

Kevin G. Volpp, MD, PhD worked on the overall development of the protocol and commented on versions of the manuscript.

David A. Asch, MD, MBA is the principal investigator for the iCOMPARE protocol and has been instrumental in all phases of protocol design and study implementation.

The iCOMPARE Research Group, comprised of all participating program directors and project staff, was instrumental in recruitment and data collection.

This work is supported by the National Heart, Lung, and Blood Institute (NHLBI), project number U01HL125388 to Dr Asch and grant number U01HL126088 to Dr Tonascia. This paper is subject to NIH Public Access Policy

Trial Registration: clinicaltrials.gov Identifier: NCT02274818.

The authors report no competing interests.

For peer review only

Figure 1 Legend: Consort Diagram

Figure 2 Legend: iCOMPARE Trial Timeline.

For peer review only

For peer review only - http://bmjopen.bmj.com/site/about/guidelines.xhtml

Table 1 Study Aims and Hypotheses

Primary Hypothesis:

Specific Aim 1: Examine patient safety and costs of care under STD and FLEX duty hour schedules.

H1a: (Primary hypothesis) 30-day patient mortality under FLEX will not exceed (will not be inferior to) mortality under STD.

Secondary Hypotheses:

H1b: 7-day and 30-day hospital readmission rates under FLEX will not exceed (will not be inferior to) the rates under STD.

H1c: Complication rates, defined by selected AHRQ Patient Safety Indicators, under Flex will not exceed (will not be inferior to) complication rates under STD.

H1d: The rate of prolonged length of stay under FLEX will not exceed (will not be inferior to) the rate of prolonged length of stay under STD.

H1e: Total costs and Medicare payments, under FLEX will not exceed (will not be inferior to) overall costs under STD.

Specific Aim 2: Examine the quality of education under STD and FLEX duty hour schedules.

Secondary Hypotheses:

H2a: Interns in FLEX will spend greater time in direct patient care and education compared to interns in STD.

H2b: Trainees in FLEX will report greater satisfaction with their educational experience (greater ownership, greater continuity and lower burnout) than trainees in STD.

H2c: Faculty in FLEX will report greater satisfaction with their clinical teaching experiences and greater perceptions of safety, teamwork and supervision than faculty in STD.

H2d: Standardized test scores for interns in Flex will not be less than (inferior to) those for interns in STD.

For peer review only

For peer review only - http://bmjopen.bmj.com/site/about/guidelines.xhtml

Specific Aim 3: Examine intern sleep time and alertness under STD and FLEX duty hour schedules.

Secondary Hypotheses:

H3a: Average daily sleep obtained by interns in FLEX will not be less than (will not be inferior to) that of interns in STD, as determined by a 14-day period of sleep monitoring using actigraphy and daily sleep diaries.

H3b: Interns in FLEX will not have (will not be inferior to) greater average subjective sleepiness via the Karolinska Sleepiness Scale (KSS)(24), or lower average behavioral alertness via the Psychomotor Vigilance Test (PVT)(25) than interns in STD, as determined by a 14-day period of morning sleepiness-alertness monitoring.

For peer review only

Table 2. Overview of Usual Care and Intervention Arm Duty Hour Requirements

**Requirement**	**Usual Care (STD; current duty hour standards)**	**Intervention (FLEX; flexible duty hour standards)**
80 hours per week (averaged over 4 weeks)	No change	No change
1 day off per week (averaged over 4 weeks)	No change	No change
In-house call no more than every 3rd night (averaged over 4-weeks)	No change	No change
PGY-1 resident duty hour periods must not exceed 16 hours	No change	Eliminated
PGY-2 and above must not work more than 24 hours with an additional 4 hours permitted for transitions in care	No change	Eliminated
Residents must have 14 hours off after 24 hours in-house duty and at least 8-10 hours off after a regular shift	No change	Eliminated

Adapted from Bilimoria KY, Chung JW, Hedges LV, et al. Development of the flexibility in duty hour requirements for surgical trainees (FIRST) trial protocol. JAMA Surg. 2016;151:273-81.

For peer review only

For peer review only - http://bmjopen.bmj.com/site/about/guidelines.xhtml

Table 3: Operationalization of Primary Education Measures

**Hypothesis**	**Primary Data Source**	**Who**	**Definition**
2a – time in direct patient care and education	Time-Motion sub-study	PGY1	percent of observed shift minutes spent in each activity
2b – Trainee satisfaction	ACGME year-end trainee survey	PGY1-3	a single item on the ACGME resident survey re: appropriate balance for education with response options of never, rarely, sometimes, often, very often
2c – Program director satisfaction	ACGME core faculty survey	Core faculty	a single item on the ACGME faculty survey that asked about residents'/fellows' workload exceeding the capacity to do the work with response options of never, rarely, sometimes, often, very often
2d- Standardized test scores	ACP in-training examination scores	PGY1	percent of questions answered correctly

PGY = post graduate year, ACGME=Accreditation Council for Graduate Medical Education, ACP=American College of Physicians

For peer review only - http://bmjopen.bmj.com/site/about/guidelines.xhtml

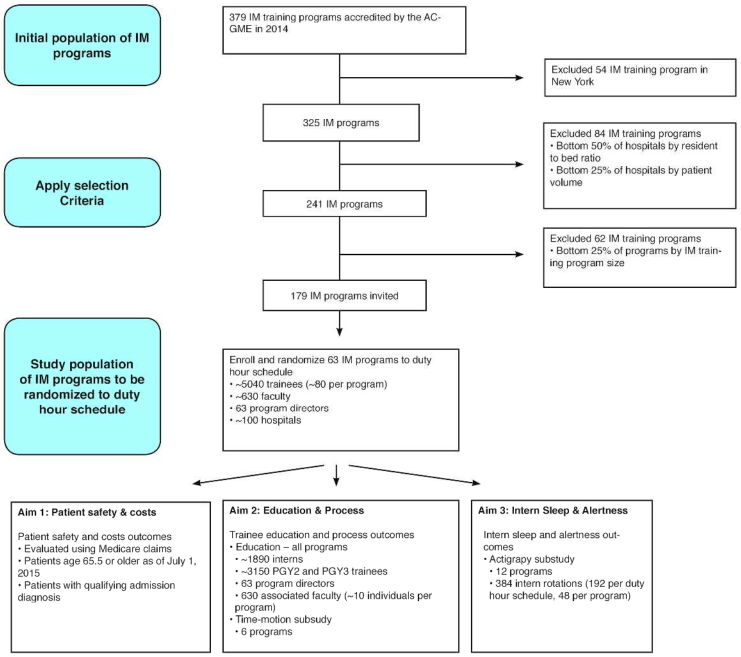

Consort Diagram

279x215mm (300 x 300 DPI)

For peer review only - http://bmjopen.bmj.com/site/about/guidelines.xhtml

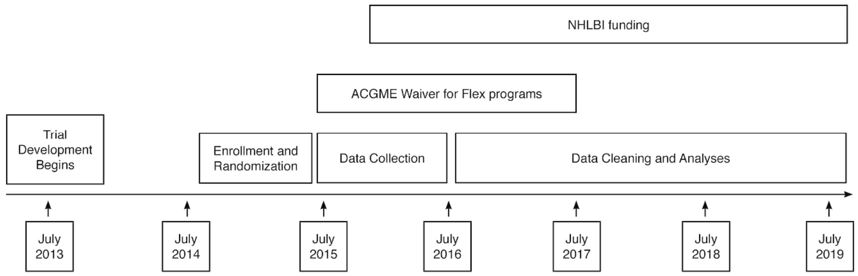

Figure 2 Legend: iCOMPARE Trial Timeline.

279x215mm (300 x 300 DPI)

w only

Development of the Individualized Comparative Effectiveness of Models Optimizing Patient Safety and Resident Education (iCOMPARE) Trial: A Protocol Summary of a National Cluster-Randomized Trial of Resident Duty Hour Policies in Internal Medicine

Judy A. Shea, PhD,[1] Jeffrey H. Silber, MD, PhD,[2] Sanjay V. Desai, MD,[3] David F. Dinges PhD,[4] Lisa M. Bellini, MD,[1] James Tonascia, PhD,[5] Alice L. Sternberg, ScM,[6] Dylan S. Small, PhD,[7] David M. Shade, JD,[6] Joel T. Katz, MD,[8] Mathias Basner, MD, PhD,[4] Krisda H. Chaiyachati, MD, MPH,[1,10] Orit Even-Shoshan, MS,[2] David W. Bates, MD, MSc,[8] Kevin G. Volpp, MD, PhD,[9,10] David A. Asch, MD, MBA[1,10] and the iCOMPARE Research Group

For peer review only

Supplementary Appendix

Table of Contents

Appendix Materials 1. Rationale and ICD-9 Codes for Principal Diagnoses Qualifying Hospital/Patient for Inclusion in iCOMPARE Randomization/Analysis

Appendix Materials 2. Details for Secondary Outcomes for Patient Safety Aim: Prolonged Length of Stay, Readmissions, Complications, and Costs

Appendix Materials 3. Sleep Actigraphy Scoring

Appendix Figure 1. Overview of Actigraphy and PVT Scoring and Review Process

Appendix Figure 2. Sleep Scoring Matrix

For peer review only - http://bmjopen.bmj.com/site/about/guidelines.xhtml

Appendix Materials 1. Rationale and ICD-9 Codes for Principal Diagnoses Qualifying Hospital/Patient for Inclusion in iCOMPARE Randomization/Analysis

The original ICD-9 code list was created by searching for relevant codes for each medical condition that were found to be associated with high mortality rates in our preliminary 2008 data. The outcomes team reviewed the code list and expanded the code list in two ways:

- We consulted the official ICD-9 code book and looked for additional high-volume codes that were related to the medical conditions of interest. The list of proposed expansion codes was reviewed and approved by the coding consultant to the study, Dr. Patrick Romano of the University of California, Davis.
- To account for the presence of ICD-10 codes on claims with discharge dates beginning October 1, 2015, we utilized the General Equivalence Mappings (GEMs) made available by the Centers for Medicare and Medicaid Services, which provide the closest possible approximation of a translation between the ICD-9 and ICD-10 code systems. Our goal was to translate each ICD-10 code back to its most closely equivalent ICD-9 code, and our review of the crosswalk caused us to pick up a small number of additional ICD-9 codes. As with the original code list expansion, these proposed additional codes were reviewed and approved by our coding consultant to the study.

Pneumonia:

481	Pneumococcal Pneumonia [Streptococcus Pneumoniae Pneumonia]
482	Other Bacterial Pneumonia
482.1	Pneumonia Due to Pseudomonas
482.2	Pneumonia Due to Hemophilus Influenzae (H. Influenzae)
482.3	Pneumonia Due to Streptococcus

For peer review only - http://bmjopen.bmj.com/site/about/guidelines.xhtml

482.31	Pneumonia Due to Streptococcus Group A
482.32	Pneumonia Due to Streptococcus Group B
482.39	Pneumonia Due to Other Streptococcus
482.41	Methicillin Susceptible Pneumonia Due to Staphylococcus Aureus
482.42	Methicillin Resistant Pneumonia Due to Staphylococcus Aureus
482.49	Other Staphylococcus Pneumonia
482.82	Pneumonia Due to Escherichia Coli [E.Coli]
482.83	Pneumonia Due to Other Gram-Negative Bacteria
482.84	Pneumonia Due to Legionnaires' Disease
482.89	Pneumonia Due to Other Specified Bacteria
482.4	Pneumonia Due to Staphylococcus
482.9	Bacterial Pneumonia Unspecified
483	Pneumonia Due to Other Specified Organism
483.1	Pneumonia Due to Chlamydia
483.8	Pneumonia Due to Other Specified Organism
485	Bronchopneumonia Organism Unspecified
486	Pneumonia Organism Unspecified

For peer review only

Stroke:

430	Subarachnoid Hemorrhage
431	Intracerebral Hemorrhage
432.0	Nontraumatic Extradural Hemorrhage
432.1	Subdural Hemorrhage
432.9	Unspecified Intracranial Hemorrhage

For peer review only - http://bmjopen.bmj.com/site/about/guidelines.xhtml

433.01	Occlusion and Stenosis of Basilar Artery with Cerebral Infarction
433.11	Occlusion and Stenosis of Carotid Artery with Cerebral Infarction
433.21	Occlusion and Stenosis of Vertebral Artery with Cerebral Infarction
433.31	Occlusion and Stenosis of Multiple and Bilateral Precerebral Arteries with Cerebral Infarction
433.81	Occlusion and Stenosis of Other Specified Precerebral Artery with Cerebral Infarction
433.91	Occlusion and Stenosis of Unspecified Precerebral Artery with Cerebral Infarction
434.01	Cerebral Thrombosis with Cerebral Infarction
434.11	Cerebral Embolism with Cerebral Infarction
434.91	Cerebral Artery Occlusion Unspecified with Cerebral Infarction
436	Acute but Ill-Defined Cerebrovascular Disease

For peer review only

For peer review only - http://bmjopen.bmj.com/site/about/guidelines.xhtml

Acute Myocardial Infarction:

410.01 Acute Myocardial Infarction of Anterolateral Wall Initial Episode of Care

410.11 Acute Myocardial Infarction of Other Anterior Wall Initial Episode of Care

410.21 Acute Myocardial Infarction of Inferolateral Wall Initial Episode of Care

410.31 Acute Myocardial Infarction of Inferoposterior Wall Initial Episode of Care

410.41 Acute Myocardial Infarction of Other Inferior Wall Initial Episode of Care

410.51 Acute Myocardial Infarction of Other Lateral Wall Initial Episode of Care

410.61 True Posterior Wall Infarction Initial Episode of Care

410.71 Subendocardial Infarction Initial Episode of Care

410.81 Acute Myocardial Infarction of Other Specified Sites Initial Episode of Care

410.91 Acute Myocardial Infarction of Unspecified Site Initial Episode of Care

Gastrointestinal Hemmorhage:

456.0 Esophageal Varices with Bleeding

530.7 Gastroesophageal Laceration-Hemorrhage Syndrome

530.82 Esophageal Hemorrhage

531.00 Acute Gastric Ulcer with Hemorrhage Without Obstruction

531.01 Acute Gastric Ulcer with Hemorrhage with Obstruction

531.20 Acute Gastric Ulcer with Hemorrhage and Perforation Without Obstruction

531.21 Acute Gastric Ulcer with Hemorrhage and Perforation with Obstruction

531.40 Chronic or Unspecified Gastric Ulcer with Hemorrhage Without Obstruction

531.41 Chronic or Unspecified Gastric Ulcer with Hemorrhage with Obstruction

531.60 Chronic or Unspecified Gastric Ulcer with Hemorrhage and Perforation Without Obstruction

For peer review only - http://bmjopen.bmj.com/site/about/guidelines.xhtml

531.61	Chronic or Unspecified Gastric Ulcer with Hemorrhage and Perforation with Obstruction
532.00	Acute Duodenal Ulcer with Hemorrhage Without Obstruction
532.01	Acute Duodenal Ulcer with Hemorrhage with Obstruction
532.20	Acute Duodenal Ulcer with Hemorrhage and Perforation Without Obstruction
532.21	Acute Duodenal Ulcer with Hemorrhage and Perforation with Obstruction
532.40	Chronic or Unspecified Duodenal Ulcer with Hemorrhage Without Obstruction
532.41	Chronic or Unspecified Duodenal Ulcer with Hemorrhage with Obstruction
532.60	Chronic or Unspecified Duodenal Ulcer with Hemorrhage and Perforation Without Obstruction
532.61	Chronic or Unspecified Duodenal Ulcer with Hemorrhage and Perforation with Obstruction
533.00	Acute Peptic Ulcer of Unspecified Site with Hemorrhage Without Obstruction
533.01	Acute Peptic Ulcer of Unspecified Site with Hemorrhage with Obstruction
533.20	Acute Peptic Ulcer of Unspecified Site with Hemorrhage and Perforation Without Obstruction
533.21	Acute Peptic Ulcer of Unspecified Site with Hemorrhage and Perforation with Obstruction
533.40	Chronic or Unspecified Peptic Ulcer of Unspecified Site with Hemorrhage Without Obstruction
533.41	Chronic or Unspecified Peptic Ulcer of Unspecified Site with Hemorrhage with Obstruction
533.60	Chronic or Unspecified Peptic Ulcer of Unspecified Site with Hemorrhage and Perforation Without Obstruction

For peer review only - http://bmjopen.bmj.com/site/about/guidelines.xhtml

533.61	Chronic or Unspecified Peptic Ulcer of Unspecified Site with Hemorrhage and Perforation with Obstruction
534.00	Acute Gastrojejunal Ulcer with Hemorrhage Without Obstruction
534.01	Acute Gastrojejunal Ulcer with Hemorrhage with Obstruction
534.20	Acute Gastrojejunal Ulcer with Hemorrhage and Perforation Without Obstruction
534.21	Acute Gastrojejunal Ulcer with Hemorrhage and Perforation with Obstruction
534.40	Chronic or Unspecified Gastrojejunal Ulcer with Hemorrhage Without Obstruction
534.41	Chronic or Unspecified Gastrojejunal Ulcer with Hemorrhage with Obstruction
534.60	Chronic or Unspecified Gastrojejunal Ulcer with Hemorrhage and Perforation Without Obstruction
534.61	Chronic or Unspecified Gastrojejunal Ulcer with Hemorrhage and Perforation with Obstruction
535.01	Acute Gastritis with Hemorrhage
535.11	Atrophic Gastritis with Hemorrhage
535.21	Gastric Mucosal Hypertrophy with Hemorrhage
535.31	Alcoholic Gastritis with Hemorrhage
535.41	Other Specified Gastritis with Hemorrhage
535.51	Unspecified Gastritis and Gastroduodenitis with Hemorrhage
535.61	Duodenitis with Hemorrhage
537.83	Angiodysplasia of Stomach and Duodenum with Hemorrhage
562.02	Diverticulosis of Small Intestine with Hemorrhage
562.03	Diverticulitis of Small Intestine with Hemorrhage
562.12	Diverticulosis of Colon with Hemorrhage
562.13	Diverticulitis of Colon with Hemorrhage

For peer review only

For peer review only - http://bmjopen.bmj.com/site/about/guidelines.xhtml

569.3	Hemorrhage of Rectum and Anus
569.85	Angiodysplasia of Intestine with Hemorrhage
578.0	Hematemesis
578.1	Blood In Stool
578.9	Hemorrhage of Gastrointestinal Tract Unspecified

Heart Failure:

398.91	Rheumatic Heart Failure (Congestive)
402.01	Malignant Hypertensive Heart Disease with Heart Failure
402.11	Benign Hypertensive Heart Disease with Heart Failure
402.91	Unspecified Hypertensive Heart Disease with Heart Failure
404.01	Hypertensive Heart and Chronic Kidney Disease Malignant with Heart Failure with Chronic Kidney Disease Stage I Through Stage Iv or Unspecified
404.03	Hypertensive Heart and Chronic Kidney Disease Malignant with Heart Failure with Chronic Kidney Disease Stage V or End Stage Renal Disease
404.11	Hypertensive Heart and Chronic Kidney Disease Benign with Heart Failure with Chronic Kidney Disease Stage I Through Stage Iv or Unspecified
404.13	Hypertensive Heart and Chronic Kidney Disease Benign with Heart Failure with Chronic Kidney Disease Stage V or End Stage Renal Disease
404.91	Hypertensive Heart and Chronic Kidney Disease Unspecified with Heart Failure with Chronic Kidney Disease Stage I Through Stage Iv or Unspecified
404.93	Hypertensive Heart and Chronic Kidney Disease Unspecified with Heart Failure with Chronic Kidney Disease Stage V or End Stage Renal Disease
428	Heart Failure

For peer review only - http://bmjopen.bmj.com/site/about/guidelines.xhtml

For peer review only

428.0	Congestive Heart Failure Unspecified
428.1	Left Heart Failure
428.20	Unspecified Systolic Heart Failure
428.21	Acute Systolic Heart Failure
428.22	Chronic Systolic Heart Failure
428.23	Acute on Chronic Systolic Heart Failure
428.30	Unspecified Diastolic Heart Failure
428.31	Acute Diastolic Heart Failure
428.32	Chronic Diastolic Heart Failure
428.33	Acute on Chronic Diastolic Heart Failure
428.40	Unspecified Combined Systolic and Diastolic Heart Failure
428.41	Acute Combined Systolic and Diastolic Heart Failure
428.42	Chronic Combined Systolic and Diastolic Heart Failure
428.43	Acute on Chronic Combined Systolic and Diastolic Heart Failure
428.9	Heart Failure Unspecified

Septicemia:

038	Septicemia
038.0	Streptococcal Septicemia
038.1	Staphylococcal Septicemia
038.9	Unspecified Septicemia

Kidney Failure:

584	Acute Kidney Failure

For peer review only - http://bmjopen.bmj.com/site/about/guidelines.xhtml

For peer review only

584.9	Acute Kidney Failure Unspecified

Cardiac Disease:

427.31	Atrial Fibrillation
427.41	Ventricular Fibrillation
427.5	Cardiac Arrest

Chronic Obstructive Pulmonary Disease:

490	Bronchitis Not Specified as Acute or Chronic
491.21	Obstructive Chronic Bronchitis with (Acute) Exacerbation
491.22	Obstructive Chronic Bronchitis with Acute Bronchitis

Pancreatitis:

577.0	Acute Pancreatitis
577.9	Unspecified Disease of Pancreas

Acute Respiratory Failure:

518.81	Acute Respiratory Failure
518.84	Acute and Chronic Respiratory Failure
518.89	Other Diseases of Lung Not Elsewhere Classified
519.11	Acute Bronchospasm

Chest Pain:

786.5	Chest Pain

For peer review only

786.59	Other Chest Pain

Cellulitis:

682	Other Cellulitis and Abscess
682.6	Cellulitis and Abscess of Leg Except Foot

Coronary Atherosclerosis:

414.01	Coronary Atherosclerosis of Native Coronary Artery
414.1	Aneurysm and Dissection of Heart

Pulmonary Embolism:

415	Acute Pulmonary Heart Disease
415.12	Septic Pulmonary Embolism
415.19	Other Pulmonary Embolism and Infarction

Syncope:

780.0	Alteration of Consciousness
780.2	Syncope and Collapse
780.3	Convulsions
780.01	Coma

Intestinal Infection:

008	Intestinal Infections Due to Other Organisms
008.45	Intestinal Infection Due to Clostridium Difficile

For peer review only

For peer review only - http://bmjopen.bmj.com/site/about/guidelines.xhtml

008.49	Intestinal Infection Due to Other Organisms

Obstructive Asthma:

493.9	Asthma Unspecified
493.22	Chronic Obstructive Asthma with (Acute) Exacerbation

Bronchitis:

494.1	Bronchiectasis with Acute Exacerbation

For peer review only

For peer review only - http://bmjopen.bmj.com/site/about/guidelines.xhtml

Appendix Materials 2: Details for Secondary Outcomes for Patient Safety Aim: Prolonged Length of Stay, Readmissions, Complications, and Costs

All patient safety measures are based on information made available to researchers by the Centers for Medicare and Medicaid Services (CMS). Health services researchers have conducted research using Medicare claims files for over two decades. This information is uniformly recorded for all Medicare beneficiaries. CMS releases to researchers the de-identified claim files for hospitals' and physicians' services after validating them through an adjudicated process. All the patient safety outcomes are calculated by the outcomes team based on information from the claims data.

The reliability of the data used for calculating patient safety outcomes for mortality, readmissions, and length of stay (LOS) is excellent. The reliability of the data used to calculate complications, costs, and payments is also high, but lower than of the data used in calculating the 30- day mortality, readmissions and LOS outcomes, due to variation across hospitals in the number and may vary across hospitals due in the number of diagnostic and procedure codes recorded in the claim. However, due to the randomized nature of the study, we would expect there to be no difference in these outcomes between the two study arms (FLEX and STD). References regarding the reliability of the various patient safety measures are provided below.

In addition to mortality, the following outcomes measures were collected:

- Readmission: This calculation is based on the admission and discharge dates in the Medicare claims.
- Length of Stay and Prolonged Length of Stay: Calculated based on the admission and discharge dates of the index claim.
- Complications: Calculated using the Agency for Healthcare Research and Quality Patient Safety Indicators (see below).

For peer review only - http://bmjopen.bmj.com/site/about/guidelines.xhtml

- Costs: Calculated using inpatient, revenue center, and Part B claims. We utilize a resource costing method (more details are provided below) to estimate the costs associated with accommodations (general floor and intensive care unit), the operating room, post-discharge emergency room visits, and other services indicated by the presence of Current Procedural Terminology codes (which are translated to Relative Value Units).
- Payments: Calculated using the payment variables that appear in the inpatient, outpatient, and Part B claims. The total amounts paid by Medicare, the beneficiary, and the primary payer are summed. Year-based adjustments for inflation are applied to the payment figures.

The Patient Safety Indicators were calculated by the research team using SAS programs provided by the Agency for Healthcare Research and Quality, which were run on the Medicare claims. Some of the Patient Safety Indicators that were considered "postoperative" or "perioperative" were modified for use with the study's population of medical patients. For these Patient Safety Indicators, the portion of the code that required the patient to have had surgery was deleted.

The following Patient Safety Indicators were used:

- PSI 03 - Pressure ulcer rate
- PSI 06 - Iatrogenic pneumothorax rate
- PSI 07 - Central venous catheter-related blood stream infection rate
- PSI 08 - Postoperative hip fracture rate
- PSI 09 - Perioperative hemorrhage or hematoma rate
- PSI 10 - Postoperative physiologic and metabolic derangement rate
- PSI 11 - Postoperative respiratory failure rate
- PSI 12 - Perioperative pulmonary embolism or deep vein thrombosis rate
- PSI 13 - Postoperative sepsis rate

For peer review only - http://bmjopen.bmj.com/site/about/guidelines.xhtml

References describing the validity of the claims data used to calculate the patient safety outcomes are in the main manuscript and repeated here:

Readmissions:

- Patel MS, Volpp KG, Small DS, et al. Association of the 2011 ACGME resident duty hour reforms with mortality and readmissions among hospitalized Medicare patients. JAMA 2014;312:2364-73.
- Krumholz HM, Lin Z, Keenan PS, et al. Relationship between hospital readmission and mortality rates for patients hospitalized with acute myocardial infarction, heart failure, or pneumonia. JAMA 2013;309:587-93.

Length of Stay and Prolonged Length of Stay:

- Silber JH, Rosenbaum PR, Even-Shoshan O, et al. Length of stay, conditional length of stay, and prolonged stay in pediatric asthma. Health Serv Res 2003;38:867-86.
- Silber JH, Rosenbaum PR, Kelz RR, et al. Medical and financial risks associated with surgery in the elderly obese. Ann Surg 2012;256:79-86.
- Silber JH, Rosenbaum PR, Koziol LF, et al. Conditional length of stay. Health Serv Res 1999;34:349-63.
- Silber JH, Rosenbaum PR, Rosen AK, et al. Prolonged hospital stay and the resident duty hour rules of 2003. Med Care 2009;47:1191-200.

Patient Safety Indicators (description of PSIs):

- Rosen AK, Loveland SA, Romano PS, et al. Effects of resident duty hour reform on surgical and procedural patient safety indicators among hospitalized Veterans Health Administration and Medicare patients. Med Care 2009;47:723-31.

For peer review only - http://bmjopen.bmj.com/site/about/guidelines.xhtml

Patient Safety Indicators (validity):

- Romano PS, Geppert JJ, Davies S, et al. A national profile of patient safety in US hospitals. Health Aff. 2003;22:154–166.
- Rosen AK, Rivard P, Zhao S, et al. Evaluating the patient safety indicators: how well do they perform on Veterans Health Administration data? Med Care. 2005;43:873–884.
- Zhan C, Miller MR. Excess length of stay, charges, and mortality attributable to medical injuries during hospitalization. JAMA. 2003;290:1868–1874.

Costs are calculated using a resource utilization-based method of cost estimation. The following items, which are calculated using the Medicare claims data, are included in the total cost estimate:

- Accommodation costs, which are based on the number of general floor days and the number of intensive care unit days during the index admission. This information comes from the revenue center files.
- Operating room cost, based on the amount of time spent in the operating room (for patients who had a surgical procedure performed). This is determined using Part B claims.
- Emergency room visit fixed costs, based on post-discharge visits to the emergency room. This is determined using Part B claims.
- Costs of services provided, based on Relative Value Units (RVUs), determined using the Current Procedural Terminology codes on bills. This is determined using Part B claims.

In addition, any costs that occurred within 30 days of the index admission date, and all the costs associated with any readmissions that began within 30 days, are also included in the total cost calculation.

For peer review only - http://bmjopen.bmj.com/site/about/guidelines.xhtml

Drugs administered during the hospitalization are included in the cost calculation, but drugs outside the hospital are not. Costs of lawsuits are not available in the Medicare claims data.

Hospital medical errors that resulted in a longer length of stay or the use of additional hospital resources, such as the need to admit a patient to the intensive care unit, would be captured in the cost calculation.

For peer review only

For peer review only - http://bmjopen.bmj.com/site/about/guidelines.xhtml

Appendix Materials 3: Sleep Actigraphy Scoring

According to conventional standards, actigraphy data were classified in 1-minute epochs as wake, sleep, or missing. The first classification was performed by the algorithm of the device manufacturer (Actilife software, version 6.13.3, standard settings, Sadeh scoring algorithm). Off-wrist periods were identified by an algorithm developed by Pulsar Informatics Inc. (Philadelphia, PA), then visually checked, integrated with sleep log information provided by interns via the smartphone and, if necessary, corrected by co-investigators from Pulsar who were experienced with actigraphy scoring of large scale clinical trials. The classification into sleep, wake, and unknown followed the set of rules outlined in Appendix Figure 1. Pulsar's visual scoring of sleep was then independently checked by study investigators who are experts in sleep research. In an iterative process, any discrepancies were documented and then corrected by Pulsar, until agreement with the study investigators was reached. During this visual scoring process, both Pulsar and the study investigators were blinded to study arm (STD or FLEX). Likewise, PVT-B data were inspected by study sleep experts blinded to arm (Appendix Figure 2). PVT-B performance was classified into three categories as [1] adherent (i.e., PVT-B data reflected an effort to do the task correctly, and comments left by the subject did not suggest non-adherence), [2] possibly non-adherent (i.e., PVT-B data reflected a consistently poor effort to do the task correctly, but comments left by the subject did not suggest non-adherence), and [3] non-adherent (i.e., PVT-B data reflected a consistently poor effort to do the task correctly, and comments left by the subject did suggest non-adherence, e.g., performing the task while brushing teeth). Comments left by interns were inspected for distractions and non-fatigue related impairment and flagged accordingly. Comments that could have revealed the study arm were blacked out by Pulsar prior to classification by the study sleep experts.

For peer review only - http://bmjopen.bmj.com/site/about/guidelines.xhtml

Appendix Figure 1. Overview of Actigraphy and PVT Scoring and Review Process

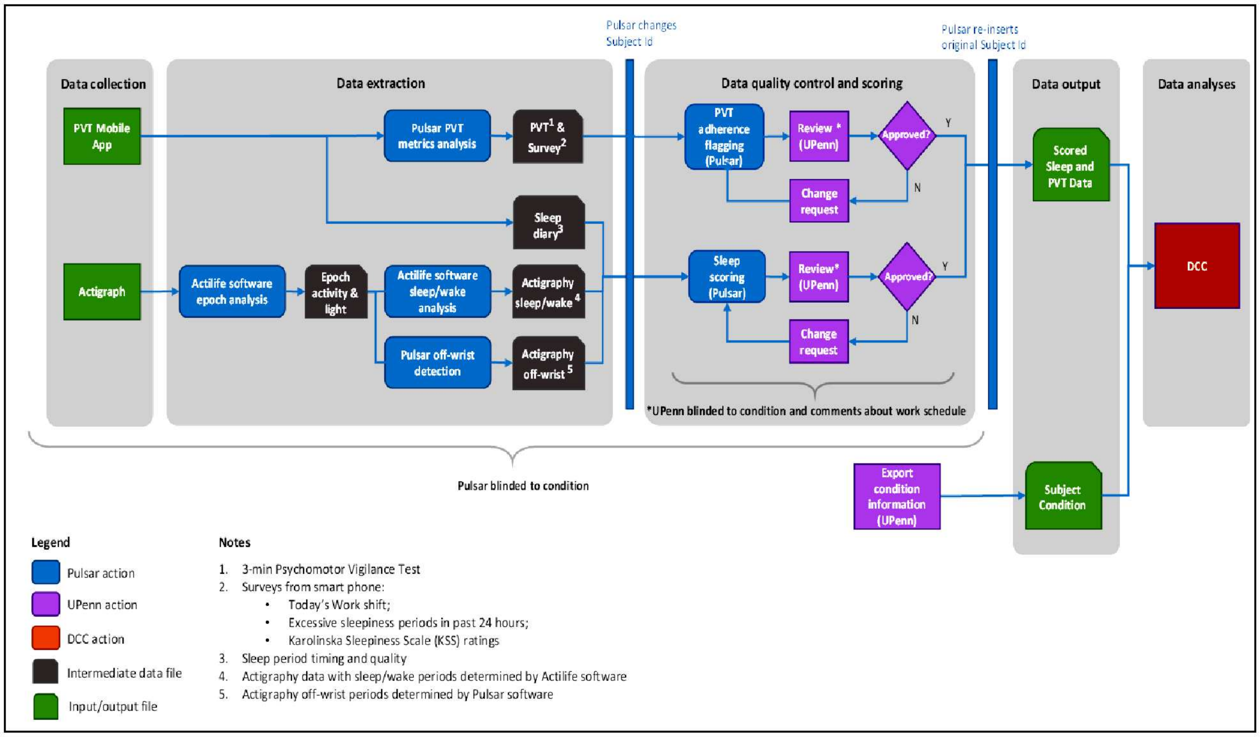

For peer review only - http://bmjopen.bmj.com/site/about/guidelines.xhtml

Appendix Figure 2: Sleep Scoring Matrix

	Sleep Diary had sleep time entered (S)	Sleep Diary did not have sleep time entered (W)
**Actigraphy Reviewed indicated sleep (S)**	Scored as Sleep (S) based on agreement of actigraphy and diary	Scored as Sleep (S) based on actigraphy
**Actigraphy Reviewed indicated wake (W)**	Scored as Wake (W) based on actigraphy	Scored as Wake (W) based on actigraphy
**Actigraphy Reviewed indicated missing or off-wrist (O)**	Scored as Sleep (S) based on diary	Scored as Unknown (U)

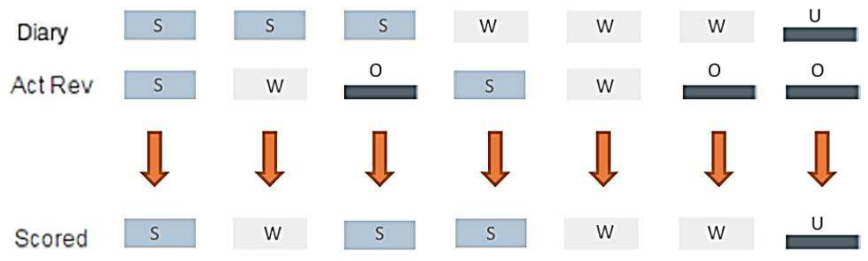

For peer review only - http://bmjopen.bmj.com/site/about/guidelines.xhtml

SPIRIT

STANDARD PROTOCOL ITEMS: RECOMMENDATIONS FOR INTERVENTIONAL TRIALS

SPIRIT 2013 Checklist: Recommended items to address in a clinical trial protocol and related documents*

Section/item	Item No	Description
**Administrative information**		
Title 1	1	Descriptive title identifying the study design, population, interventions, and, if applicable, trial acronym
Trial registration 2,4	2a	Trial identifier and registry name. If not yet registered, name of intended registry
	2b	All items from the World Health Organization Trial Registration Data Set
Protocol version	3	Date and version identifier NA
Funding 2	4	Sources and types of financial, material, and other support
Roles and responsibilities 1	5a	Names, affiliations, and roles of protocol contributors
	5b	Name and contact information for the trial sponsor
*	5c	Role of study sponsor and funders, if any, in study design; collection, management, analysis, and interpretation of data; writing of the report; and the decision to submit the report for publication, including whether they will have ultimate authority over any of these activities
*	5d	Composition, roles, and responsibilities of the coordinating centre, steering committee, endpoint adjudication committee, data management team, and other individuals or groups overseeing the trial, if applicable (see Item 21a for data monitoring committee)
**Introduction**		
Background and rationale 5-6	6a	Description of research question and justification for undertaking the trial, including summary of relevant studies (published and unpublished) examining benefits and harms for each intervention
	6b	Explanation for choice of comparators NA
Objectives 7	7	Specific objectives or hypotheses
Trial design 8,11	8	Description of trial design including type of trial (eg, parallel group, crossover, factorial, single group), allocation ratio, and framework (eg, superiority, equivalence, noninferiority, exploratory)

*In protocol, could be added to summary

For peer review only - http://bmjopen.bmj.com/site/about/guidelines.xhtml

Methods: Participants, interventions, and outcomes

Study setting	9	Description of study settings (eg, community clinic, academic hospital) and list of countries where data will be collected. Reference to where list of study sites can be obtained	8
Eligibility criteria	10	Inclusion and exclusion criteria for participants. If applicable, eligibility criteria for study centres and individuals who will perform the interventions (eg, surgeons, psychotherapists)	9-11
Interventions	11a	Interventions for each group with sufficient detail to allow replication, including how and when they will be administered	Table2, 30
	11b	Criteria for discontinuing or modifying allocated interventions for a given trial participant (eg, drug dose change in response to harms, participant request, or improving/worsening disease)	*
	11c	Strategies to improve adherence to intervention protocols, and any procedures for monitoring adherence (eg, drug tablet return, laboratory tests)	
	11d	Relevant concomitant care and interventions that are permitted or prohibited during the trial	
Outcomes	12	Primary, secondary, and other outcomes, including the specific measurement variable (eg, systolic blood pressure), analysis metric (eg, change from baseline, final value, time to event), method of aggregation (eg, median, proportion), and time point for each outcome. Explanation of the clinical relevance of chosen efficacy and harm outcomes is strongly recommended	Table3, 31, 12-17
Participant timeline	13	Time schedule of enrolment, interventions (including any run-ins and washouts), assessments, and visits for participants. A schematic diagram is highly recommended (see Figure)	12, Figure 2
Sample size	14	Estimated number of participants needed to achieve study objectives and how it was determined, including clinical and statistical assumptions supporting any sample size calculations	8-9
Recruitment	15	Strategies for achieving adequate participant enrolment to reach target sample size	10

Methods: Assignment of interventions (for controlled trials)

Allocation:

Sequence generation	16a	Method of generating the allocation sequence (eg, computer-generated random numbers), and list of any factors for stratification. To reduce predictability of a random sequence, details of any planned restriction (eg, blocking) should be provided in a separate document that is unavailable to those who enrol participants or assign interventions	11

For peer review only - http://bmjopen.bmj.com/site/about/guidelines.xhtml

Allocation concealment mechanism	16b	Mechanism of implementing the allocation sequence (eg, central telephone; sequentially numbered, opaque, sealed envelopes), describing any steps to conceal the sequence until interventions are assigned	
Implementation	16c*	Who will generate the allocation sequence, who will enrol participants, and who will assign participants to interventions	
Blinding (masking)	17a	Who will be blinded after assignment to interventions (eg, trial participants, care providers, outcome assessors, data analysts), and how	
	17b	If blinded, circumstances under which unblinding is permissible, and procedure for revealing a participant's allocated intervention during the trial	
**Methods: Data collection, management, and analysis**			
Data collection methods	18a	Plans for assessment and collection of outcome, baseline, and other trial data, including any related processes to promote data quality (eg, duplicate measurements, training of assessors) and a description of study instruments (eg, questionnaires, laboratory tests) along with their reliability and validity, if known. Reference to where data collection forms can be found, if not in the protocol	10, 12-16
	18b	Plans to promote participant retention and complete follow-up, including list of any outcome data to be collected for participants who discontinue or deviate from intervention protocols	NA
Data management	19*	Plans for data entry, coding, security, and storage, including any related processes to promote data quality (eg, double data entry; range checks for data values). Reference to where details of data management procedures can be found, if not in the protocol	
Statistical methods	20a	Statistical methods for analysing primary and secondary outcomes. Reference to where other details of the statistical analysis plan can be found, if not in the protocol	17
	20b	Methods for any additional analyses (eg, subgroup and adjusted analyses)	
	20c*	Definition of analysis population relating to protocol non-adherence (eg, as randomised analysis), and any statistical methods to handle missing data (eg, multiple imputation)	
**Methods: Monitoring**			
Data monitoring	21a	Composition of data monitoring committee (DMC); summary of its role and reporting structure; statement of whether it is independent from the sponsor and competing interests; and reference to where further details about its charter can be found, if not in the protocol. Alternatively, an explanation of why a DMC is not needed	6-7

	21b	Description of any interim analyses and stopping guidelines, including who will have access to these interim results and make the final decision to terminate the trial
Harms	22	Plans for collecting, assessing, reporting, and managing solicited and spontaneously reported adverse events and other unintended effects of trial interventions or trial conduct
Auditing	23	Frequency and procedures for auditing trial conduct, if any, and whether the process will be independent from investigators and the sponsor

Ethics and dissemination

Research ethics approval	24 11	Plans for seeking research ethics committee/institutional review board (REC/IRB) approval
Protocol amendments	25 NA	Plans for communicating important protocol modifications (eg, changes to eligibility criteria, outcomes, analyses) to relevant parties (eg, investigators, REC/IRBs, trial participants, trial registries, journals, regulators)
Consent or assent	26a	Who will obtain informed consent or assent from potential trial participants or authorised surrogates, and how (see Item 32)
	26b	Additional consent provisions for collection and use of participant data and biological specimens in ancillary studies, if applicable
Confidentiality	27	How personal information about potential and enrolled participants will be collected, shared, and maintained in order to protect confidentiality before, during, and after the trial
Declaration of interests	28	Financial and other competing interests for principal investigators for the overall trial and each study site
Access to data	29	Statement of who will have access to the final trial dataset, and disclosure of contractual agreements that limit such access for investigators
Ancillary and post-trial care	30	Provisions, if any, for ancillary and post-trial care, and for compensation to those who suffer harm from trial participation
Dissemination policy	31a	Plans for investigators and sponsor to communicate trial results to participants, healthcare professionals, the public, and other relevant groups (eg, via publication, reporting in results databases, or other data sharing arrangements), including any publication restrictions
	31b	Authorship eligibility guidelines and any intended use of professional writers
	31c	Plans, if any, for granting public access to the full protocol, participant-level dataset, and statistical code

Appendices

Informed consent materials	32	Model consent form and other related documentation given to participants and authorised surrogates
Biological specimens	33	Plans for collection, laboratory evaluation, and storage of biological specimens for genetic or molecular analysis in the current trial and for future use in ancillary studies, if applicable

*It is strongly recommended that this checklist be read in conjunction with the SPIRIT 2013 Explanation & Elaboration for important clarification on the items. Amendments to the protocol should be tracked and dated. The SPIRIT checklist is copyrighted by the SPIRIT Group under the Creative Commons "Attribution-NonCommercial-NoDerivs 3.0 Unported" license.

BMJ Open

Development of the Individualized Comparative Effectiveness of Models Optimizing Patient Safety and Resident Education (iCOMPARE) Trial: A Protocol Summary of a National Cluster-Randomized Trial of Resident Duty Hour Policies in Internal Medicine

Journal:	*BMJ Open*
Manuscript ID	bmjopen-2018-021711.R2
Article Type:	Protocol
Date Submitted by the Author:	03-Aug-2018

Complete List of Authors:	Shea, Judy; University of Pennsylvania Perelman School of Medicine, Medicine<br>Silber, JH; The Children's Hospital of Philadelphia, Pediatrics<br>Desai, Sanjay; The Johns Hopkins University, Medicine<br>Dinges, David ; University of Pennsylvania Perelman School of Medicine, Psychiatry<br>Bellini, Lisa; University of Pennsylvania Perelman School of Medicine, Medicine<br>Tonascia, James; John Hopkins , Biostatistics<br>Sternberg, Alice; Johns Hopkins Hospital and Health System, Epidemiology<br>Small, Dylan; University of Pennsylvania, Wharton Statistics<br>Shade, David ; Johns Hopkins University Bloomberg School of Public Health, Epidemiology<br>Katz, Joel; Brigham and Women's Hospital , Medicine<br>Basner, Mathias; University of Pennsylvania Perelman School of Medicine, Psychiatry<br>Chaiyachati, Krisda; University of Pennsylvania Perelman School of Medicine, Medicine ; Cpl Michael J Crescenz VA Medical Center<br>Even-Shoshan, Orit; Children's Hospital of Philadelphia , Pediatrics<br>Bates, David ; Brigham and Women's Hospital, Medicine<br>Volpp, Kevin ; University of Pennsylvania Perelman School of Medicine, Medical Ethics and Policy; Cpl Michael J Crescenz VA Medical Center<br>Asch, David; University of Pennsylvania Perelman School of Medicine, Medicine ; Cpl Michael J Crescenz VA Medical Center<br>Research Group , iCOMPARE

**Primary Subject Heading**:	Medical education and training
Secondary Subject Heading:	Medical education and training, Evidence based practice, Health services research
Keywords:	resident education, duty hours, resident work hours

SCHOLARONE™
Manuscripts

For peer review only

Development of the Individualized Comparative Effectiveness of Models Optimizing Patient Safety and Resident Education (iCOMPARE) Trial: A Protocol Summary of a National Cluster-Randomized Trial of Resident Duty Hour Policies in Internal Medicine

Judy A. Shea, PhD,[1] Jeffrey H. Silber, MD, PhD,[2] Sanjay V. Desai, MD,[3] David F. Dinges PhD,[4] Lisa M. Bellini, MD,[1] James Tonascia, PhD,[5] Alice L. Sternberg, ScM,[6] Dylan S. Small, PhD,[7] David M. Shade, JD,[6] Joel T. Katz, MD,[8] Mathias Basner, MD, PhD,[4] Krisda H. Chaiyachati, MD, MPH,[1,10] Orit Even-Shoshan, MS,[2] David W. Bates, MD, MSc,[8] Kevin G. Volpp, MD, PhD,[9,10] David A. Asch, MD, MBA[1,10] and the iCOMPARE Research Group

[1]Department of Medicine, University of Pennsylvania, Philadelphia PA, USA

[2]Department of Pediatrics, Children's Hospital of Philadelphia, Philadelphia PA, USA

[3]Department of Medicine, the Johns Hopkins University, Baltimore MD, USA

[4]Department of Psychiatry, University of Pennsylvania, Philadelphia PA, USA

[5]Department of Biostatistics, the Johns Hopkins University, Baltimore MD, USA

[6]Department of Epidemiology, the Johns Hopkins University, Baltimore MD, USA

[7]Wharton Statistics Department, University of Pennsylvania, Philadelphia PA, USA

[8]Department of Medicine, Brigham and Women's Hospital, Boston, MA, USA

[9]Department of Medical Ethics and Policy, University of Pennsylvania, Philadelphia PA, USA

[10]the Cpl Michael J Crescenz VA Medical Center, Philadelphia PA

For peer review only - http://bmjopen.bmj.com/site/about/guidelines.xhtml

Acknowledgements: The iCOMPARE team extends their appreciation to the participating Program Directors, Jeremy M Asch, BA, Amanda K Bertram, MS, Michele M Carlin BA, Sara C Coats, BS, Adrian J. Ecker, Kelsey A. Gangemi, MPH, Alexander S. Hill, BS, Susan K. Malone, PhD, RN, Alyssa M. Yeager, MD and Pulsar Informatics for their support of the trial. The trial was funded by grants from the National Heart, Lung, and Blood Institute (NHLBI); grant number U01HL125388 to Dr. Asch and grant number U01HL126088 to Dr. Tonascia) and the Accreditation Council for Graduate Medical Education.

Corresponding author:
Judy A. Shea, Ph.D.
1317 Blockley Hall
423 Guardian Drive
Philadelphia, PA 19104-6021
215 573-5111
215 573-8778 (fax)
sheaja@pennmedicine.upenn.edu

This paper is subject to NIH Public Access Policy

Trial Registration: clinicaltrials.gov Identifier: NCT02274818

Running title: iCOMPARE Trial of Resident Duty Hour Policies

Number of references: 49

Number of tables: 3

Number of figures: 2

Number of supplementary appendices: 1

Word Count: 5116

Word count abstract: 274

For peer review only

Key words: resident education, duty hours, resident work hours,

For peer review only - http://bmjopen.bmj.com/site/about/guidelines.xhtml

Abstract

Introduction: Medical trainees' duty hours have received attention globally; restrictions in Europe, New Zealand and some Canadian provinces are much lower than the 80-hours per week enforced in the United States (U.S.). In the U.S., resident duty hours have been implemented without evidence simultaneously reflecting competing concerns about patient safety and physician education. The objective is to prospectively evaluate the implications of alternative resident duty hour rules for patient safety, trainee education, and intern sleep and alertness.

Methods and Analysis. 63 U.S. internal medicine training programs were randomly assigned 1:1 to the 2011 Accreditation Council for Graduate Medical Education resident duty hour rules or to rules more flexible in intern shift length, and number of hours off between shifts for academic year 2015-2016. The primary outcome is calculated for each program as the difference in 30 day mortality rate among Medicare beneficiaries with any of several pre-specified principal diagnoses in the intervention year minus 30-day mortality in the pre-intervention year among Medicare beneficiaries with any of several pre-specified principal diagnoses. Additional safety outcomes include readmission rates, prolonged length of stay, and costs. Measures derived from trainees' and faculty responses to surveys and from time-motion studies of interns compare the educational experiences of residents. Measures derived from wrist actigraphy, subjective ratings and psychomotor vigilance testing compare the sleep and alertness of interns. Differences between duty hour groups in outcomes will be assessed by intention-to-treat analyses.

Ethics and dissemination. The University of Pennsylvania Institutional Review Board (IRB) approved the protocol and served as the IRB of record for 40 programs that agreed to sign an Institutional Affiliation Agreement. Twenty-three programs opted for a local review process.

For peer review only - http://bmjopen.bmj.com/site/about/guidelines.xhtml

Trial Registration: clinicaltrials.gov Identifier: NCT02274818

Article Summary:

Strengths and limitations of this study:

iCOMPARE is the largest randomized trial examining the impact of duty hours in internal medicine training programs.

The aims are broad and include patient safety, trainees' sleep and alertness and a host of education outcomes.

The protocol draws on a number of data collection methods including real time direct observation of trainee activities, existing and study administered surveys, robust recording of sleep periods over a 14-day period and patient medical record data.

The lag between the observation period and data release for patient medical record data is substantial.

iCOMPARE promises narrow but substantive evidence to inform resident duty hour standards in internal medicine and signals new interest in methodologically stronger research in medical education.

For peer review only - http://bmjopen.bmj.com/site/about/guidelines.xhtml

Introduction

The long hours worked by resident physicians received some academic attention in the 1970s in the United States (U.S.) [1]. It was not until 1984 when those long hours became publicly linked with concerns about patient safety.[2] Safety concerns resonated with a public who found it self-evident that the often 30-hour shifts of resident physicians would lead to fatigue, and that fatigue would lead to errors that would harm patients. Notably, the possible link between duty hours and patient safety was not just a U.S. concern. Many countries began to limit hours in the 1990s. For example, New Zealand has had a limit of 72 hours a week since 1985. [3] The European Working Time Directive has included doctors in training in their limits of 48 hours per week since 2000 (but Denmark has a normal work week of 37 hours). Australia and many Canadian provinces set limits on some shift lengths given concerns for patient and trainee safety. [4] The U.S. was a little slower to come to the table. Although regulation of duty hours across all specialties occurred first in New York State as a reaction to the Libby Zion case, [2] it was not until 2003 when the Accreditation Council for Graduate Medical Education (ACGME)—the organization that oversees resident education in the U.S.—limited residents to 80 hours of work per week averaged over 4 weeks, and limited the length of individual shifts to 24 hours, with an additional 4 hours to safely transfer care to the next resident. Partly on the basis of an Institute of Medicine report and a trial from Brigham and Women's Hospital,[5,6] those regulations were further tightened for first year residents (interns) in 2011, limiting their maximum shift length to 16 hours. This change prompted a charged debate.[7] Proponents argued that the restrictions did not go far enough. Others argued that the regulations were overly restrictive and inflexible, and harbored increased risk to patients by increasing patient handoffs.[8,9]

Meanwhile, large observational studies, using data from Medicare and the Veterans Administration across millions of hospitalizations, found essentially no difference in important patient outcomes

For peer review only - http://bmjopen.bmj.com/site/about/guidelines.xhtml

following implementation of either the 2003 [10-18] or 2011 duty hour standards.[19] Several studies associated the 2011 standards with less direct patient contact, increased perception of medical errors, increased transitions of care, decreased educational opportunities, and only modestly increased quantities of sleep.[20-22] Others found no changes in trainees' educational test scores.[23] Program directors and trainees expressed concern that the rules reduced training quality and increased rather than decreased medical errors.[24-26] The increasing recognition of the importance of supervision – with separate mandates implemented by the ACGME over the same period – added further uncertainty to the debate. In the end, a well-meaning effort to manage resident fatigue was perceived by many to promote burnout, increase handoffs, decrease educational opportunities and delay the professional maturation required to produce competent, independent physicians. Currently, the evidence available to resolve these controversies is limited to a patchwork of laboratory and in vivo studies of sleep deprivation, large scale epidemiological observations of patient outcomes, surveys of resident and educator opinions, and often single-center trials of unique residency duty hour designs that focused on workload or sleep but not patient outcomes.

In this context, we created the iCOMPARE (individualized Comparative Effectiveness of Models Optimizing Patient Safety and Resident Education) trial, a cluster-randomized trial carried out by internal medicine (IM) residency programs in the U.S. during the 2015-2016 academic year. Participating residency programs were randomized to one of two groups: 1) maintain standard (STD) duty hour rules, or 2) permit a more flexible (FLEX) set of duty hour rules, noted principally for removing the 16-hour shift length restriction for interns and allowing them to work up to 24 hours with an additional 4 hours for care transitions. In contrast to prior work, iCOMPARE was designed to simultaneously assess the impact of duty hour rules on patient safety, resident education, and intern sleep and alertness.

For peer review only - http://bmjopen.bmj.com/site/about/guidelines.xhtml

While the iCOMPARE trial was being planned, leaders in surgical education developed a roughly parallel trial, the Flexibility in Duty Hours Requirements for Surgical Trainees (FIRST) trial, which was fielded in the 2014-2015 academic year. The design of the FIRST trial and its initial results have been published.[27,28] Here we describe the design of the iCOMPARE trial.

Funding and Organization

The iCOMPARE trial is funded primarily by the National Heart, Lung, and Blood Institute (NHLBI), with additional funding from the ACGME. The NHLBI appointed an independent Data and Safety Monitoring Board (DSMB) to advise the Institute regarding the trial's progress, monitor data quality and safeguard the interests of study participants.

Study Aims and Hypotheses

Study hypotheses are presented in Table 1. The primary hypothesis for the trial is that 30-day any-site patient mortality under FLEX will not exceed (will not be inferior to) 30-day patient mortality under STD, measured as the difference in difference across STD and FLEX programs between a program's 30-day patient mortality rate in the trial year and that rate in the pre-trial year (i.e., 30-day patient mortality rate at a program during the trial year minus 30-day patient mortality rate at the program in the pre-trial year). Secondary outcomes related to patient safety include 7-day and 30-day hospital readmission rates, complication rates, the probability of a prolonged length of stay, total resource utilization, and Medicare payments.

ICOMPARE's education hypotheses are that trainees in the FLEX arm will spend more time in direct patient care and education and have greater satisfaction with educational experiences compared to their STD arm peers; that standardized test scores for interns in FLEX will not be lower than such scores for interns in the STD arm; that faculty in the FLEX arm will report greater satisfaction with their clinical

For peer review only - http://bmjopen.bmj.com/site/about/guidelines.xhtml

teaching experiences and greater perceptions of safety, teamwork and supervision than faculty in the STD arm.

ICOMPARE's sleep hypotheses are that the average daily sleep will not be less among interns in the FLEX arm compared to those in the STD arm, and that interns in the FLEX arm will not have greater subjective sleepiness or lower behavioral alertness than interns in the STD arm.

Methods and Analysis

Study Design

iCOMPARE is a cluster-randomized trial to compare two alternative duty hour standards in 63 IM training programs in the United States fielded in academic year 2015-2016. Table 2 compares the duty hour standards between the FLEX and STD arms.

Cluster randomization occurred at the level of the residency program, the level at which duty hour policies are implemented. Although duty hour standards are mandated, individual programs vary considerably in how they schedule their trainees within those standards. The trial is pragmatic in that the intervention arm effectively represents the exposure of residency programs to an alternative set of duty hour standards. The exposure is the policy change, not the actual duty hours that are implemented in response. This approach is akin to clinical trials of outpatient pharmaceuticals in which the exposure is the prescription of the control or intervention drug, regardless of participant adherence.

Hypotheses regarding how interns spend their time require detailed time-motion observations, and hypotheses about interns' sleep and alertness require detailed observations of sleep cycles and psychomotor vigilance—both accomplished through sub-studies deployed in a sample of participating programs.

Sample Size and Power

For peer review only - http://bmjopen.bmj.com/site/about/guidelines.xhtml

For peer review only

The non-inferiority 30-day patient mortality hypothesis (H1a) is the trial's primary hypothesis. Defining the outcome as the trial year rate minus the pre-trial year rate adjusts each program's outcome for secular trends in 30-day mortality, as well as for differential program-to-program patient risk profiles. Using a two-sample t-test for non-inferiority of the between group mean year over year difference in 30-day mortality and assuming 80% power, Type I error of 5%, a non-inferiority margin of 1%, pooled SD for the outcome of 1.5%, and a 30-day mortality rate of 11%, we calculated a required sample of 58 programs, 29 per treatment group. The pooled SD of the outcome (year over year difference in 30-day mortality) and the 30-day mortality rate in the STD group were estimated using available Medicare claims from 2007-2008 for the population of target IM programs (i.e., all IM Programs meeting the trial's size and population criteria).

This number of programs and the associated trainees and faculty provide excellent power for analyses investigating the education hypotheses which use person-level outcomes. Assuming 30 interns, 50 second year or higher trainees, 10 faculty members, and 1 director per program, iCOMPARE was projected to include 1740 interns, 2900 second year or higher trainees, 580 faculty members, and 58 program directors.

Hypothesis H2a addresses the Flex versus STD difference in percent of time spent by interns in direct patient care and was assessed with a Time-Motion study. The sample size of the Time-Motion study is 6 programs (3 FLEX, 3 STD; 60 interns, 10 interns per program). Preliminary data [29,30] suggested that the mean percent time spent in direct patient care in STD programs would be 13% ± 4%. The Time-Motion study has at least 80% power to detect a 3% difference between FLEX and STD in time spent in direct care.

For peer review only - http://bmjopen.bmj.com/site/about/guidelines.xhtml

Hypothesis H3a addresses the FLEX versus STD difference in average intern daily sleep over a 14-day period. Targeting 90% power, one-sided Type I error of 5%, a non-inferiority margin of 0.5 hours, and assuming expected average daily sleep in STD of 6.9 ± 1.5 hours [31], the sample size for the Sleep and Alertness study was calculated to be 290 interns (145 per treatment group) and increased to 384 interns (192 per treatment group) to anticipate data loss related to non-adherence and dropouts.

Study Population and Inclusion Criteria

The CONSORT diagram is provided in Figure 1. In 2014 there were 379 IM ACGME-accredited training programs in the country not on probation. The 54 programs in New York State were excluded because that state's legislated duty hour standards were not subject to the waiver required for the intervention arm. Since the patient safety outcomes would be measured in Medicare patients, Veterans Administration hospitals did not qualify for inclusion. Sufficiently precise estimates of our patient safety outcome measures necessitated exclusion of 84 programs training only at hospitals in the bottom 50% by trainee-to-bed ratio or the bottom 25% by patient volume in the measured diagnoses. In about 50% of teaching hospitals, the number of residents per bed is so few that the residents' impact on patient care is minimal, and so any changes in the impact of those residents' schedules would be too insignificant to measure.

An additional 62 programs in the lowest quartile of number of trainees were excluded to ensure measurement of enough trainees. Two other programs in the lowest quartile of number of trainees were allowed by the Steering Committee. One program was approved for inclusion despite deviations in size of its two affiliated hospitals because together the two hospitals met the qualifying hospital size. A second program was approved for inclusion because the hospital it was affiliated with had far exceeded the trainee-to-bed ratio even though the patient volume was slightly low. A total of 179 programs were eligible for inclusion.

For peer review only - http://bmjopen.bmj.com/site/about/guidelines.xhtml

Recruitment

Directors of eligible programs were invited to participate by letters of invitation and informational sessions during the Fall 2014 and Spring 2015 Association of Program Directors in Internal Medicine (APDIM) meetings. Participation required agreement to support study needs and a participation agreement signed by the Designated Institutional Official indicating approval from the institution's Graduate Medical Education Committee. Program directors indicated the hospitals, and the trainee rotations within those hospitals, which would participate in iCOMPARE. Each program was reviewed by the study team to confirm that at least one of the hospitals indicated by the program director qualified for inclusion in the trial. The study team checked the list of hospitals provided by the program director for shared staffing with other training programs eligible for and interested in iCOMPARE so that those programs could be randomized together and avoid having different duty hour schedules for trainees at a common hospital. This occurred for 3 pairs of hospitals. In total, 63 programs agreed to participate and were randomized.

Patient and Public Involvement

The study was based on a fundamental concern about the impact of training schedules on patient, trainee and program director experiences. However, no patients were directly involved in the design and conduct of the study. Program directors from each participating program were involved and essential to recruitment which was at the training program level. Program directors were fully informed about the intervention and individual trainees had the opportunity to decline participation in surveys and other education outcomes. Results will be presented at national meetings to program directors and published in peer-reviewed journals.

For peer review only - http://bmjopen.bmj.com/site/about/guidelines.xhtml

Institutional Review Board Determinations

The University of Pennsylvania Institutional Review Board (IRB) approved the iCOMPARE protocol and served as the IRB of record for all participating programs that agreed to sign an Institutional Affiliation Agreement. Forty programs chose this option and submitted the required paperwork. Twenty-three programs opted for a local review process.

Randomization

Randomization was 1:1 into the STD and FLEX arms. Participating programs in iCOMPARE were not masked to their assignment. Sixty programs completed the application process quickly and were randomized in November 2014 so that they could provide applicants interviewing for positions in academic year 2015-2016 information about the duty hour arm assignment. Randomization of programs ended in March 2015, judged to be the latest date by which a program could be randomized to the FLEX arm and have adequate time to adjust their residency schedules in anticipation of the 2015-2016 intervention year.

Data Sources

Hypothesis testing required outcome data from patients, trainees, and program directors and faculty. Because of the available and highly reliable patient mortality information (both in hospital and after discharge) in the Medicare fee-for-service (FFS) program, the iCOMPARE patient population was limited to Medicare FFS beneficiaries with a qualifying principal diagnosis on hospital admission (Supplementary Appendix: Appendix Materials 1); these diagnoses were chosen for their common treatment on internal medicine services (excluding oncology and neurology diagnoses) and their elevated mortality rates. Because similar data are not available for patients enrolled in a Medicare managed care program,

For peer review only - http://bmjopen.bmj.com/site/about/guidelines.xhtml

patients enrolled in a Medicare managed care program six months before or one month post discharge for the index hospital admission were excluded.

Randomized programs were further invited to participate in the observational Time-Motion and the Sleep and Alertness sub-studies, described below.

Timeline

Trial development began in 2013. The Medicare data required for the analyses of the patient safety outcomes are not expected to be available until mid 2018 (Figure 2).

Outcomes, Measures, and Data Collection

Aim 1 – Patient Safety

Patient safety measures are derived from Medicare data, collected as part of routine clinical practice and administration and obtained through ResDAC (https://www.resdac.org/). The primary outcome is based on 30-day (from admission) all-location mortality, a patient-centered measure unaffected by length of hospitalization.[10,11,15,18,19,32,33] Secondary outcomes include prolonged length of stay,[14,34-36] 30-day readmission rate, [19,37] complication rate,[13,34,38,39] and resource utilization measures (cost) [17,35] and payment among fee-for-service Medicare beneficiaries with specific diagnoses. Details regarding justification for and operationalization of these outcomes are provided in Supplementary Appendix: Appendix Materials 2.

Aim 2 – Education

Education measures are derived from multiple sources. The primary education measures are specified in Table 3 and come from the Time-Motion sub-study, the ACGME year-end trainee and core faculty surveys, and the interns' In-Training Examination (ITE) scores provided by the American College of

For peer review only - http://bmjopen.bmj.com/site/about/guidelines.xhtml

Physicians (ACP). Secondary education measures include iCOMPARE-generated surveys and survey data provided by the Association of Program Directors in Internal Medicine (APDIM).

Time-Motion sub-study. We recruited 3 IM programs randomized to STD and 3 randomized to FLEX to participate in direct observations of some of their interns, targeting programs in the mid-Atlantic region for operational convenience; programs with tertiary hospitals as well as community-based programs were included in both arms. Participating programs received $3,000 to support these sub-study activities. We recruited interns rotating on General Medicine services between March-May 2016. Eligible and interested interns provided written consent. Among the 129 interns invited to participate, 120 (93%) consented.

Twenty-three observers (medical students and undergraduates) were trained to follow participating interns. They used a custom-built tablet-based software program to document start and stop times for various intern activities: direct patient care, indirect patient care, education, rounds, work, handoffs, and miscellaneous, each with various subcategories reflecting greater specificity of tasks. For example, direct patient care had subcategories for patient interactions, family interactions, and physical contact (e.g., physical exam). At least one activity had to be selected at all times, although more than one could be selected to reflect multitasking. At the start and stop of the shift an observer completed brief surveys that summarized total patient census numbers for that intern, including the numbers of transfers, discharges, admissions, and patients received at the beginning of a shift and handed-off at the end.

Shifts were selected at each site aiming to capture 30 shifts in a proportion mirroring – specific for each site – how interns generally spend their time on a general medicine inpatient rotation in a given week. Observers shadowed approximately 8-10 shifts, for 1-3 shifts per intern. Extended shifts (e.g., 24 hour call cycles) were often split by two observers. A 10% sample of shifts was observed simultaneously by two observers to estimate inter-rater reliability. The methods for the Time-Motion sub-study were

For peer review only - http://bmjopen.bmj.com/site/about/guidelines.xhtml

based on previous research in terms of the structure of observations (e.g. categories used), process of observations (e.g. platform used and scheduling strategies), and the training of observers [27,28]. Inter-rater reliability was high for pairs of observers using a training video (median kappa = .67 and median agreement of 90%) as well as the real-time observations (median kappa of .74 and median agreement of 89%). Interns received a $50 Amazon gift card for each observed shift.

ACGME end-of-year survey data. The ACGME provided iCOMPARE with summary data derived from their end-of-year surveys of trainees and core faculty in May 2014, May 2015, and May 2016; all data provided by the ACGME to iCOMPARE was at the level of treatment group arm (FLEX or STD) and year collected (2014, 2015, 2016). All data were stripped of program identifiers and individual respondent identifiers. iCOMPARE received summary treatment-group level information for the single question items specified in hypotheses H2b and H2c and many content areas (combined responses over several questions covering related content) that comprise secondary education outcomes.

ITE score data from ACP. The ACP provided iCOMPARE with trainee level ITE scores from 2015 and 2016 for those trainees who had given permission to share their score for research purposes. Identifying information for a score was limited to PGY year and program identifier.

APDIM annual survey data. The APDIM provided data from its survey of program directors in fall 2015 and fall 2016. The deidentified data set did not include name of program or program director but did have a flag for STD or FLEX or not in study.

End-of-shift Surveys. From August 31, 2015 to April 26, 2016, iCOMPARE conducted 16 two-week cycles of daily surveys administered to all trainees at all participating programs. At the start of each cycle, we randomized each trainee into one of 14 groups and then allocated each group to a day of the 2-week period to receive a survey. Thus, each trainee was surveyed once every two weeks.

For peer review only - http://bmjopen.bmj.com/site/about/guidelines.xhtml

Surveys alternated between two sets of questions. Survey 1 was sent every other day and asked: [1] the name of the rotation a trainee was on in the past 24 hours, specifying details such as inpatient or not, type of inpatient rotation, at main teaching hospital or other setting, or not in hospital; and if the trainee was on an inpatient rotation; [2] number of new patient evaluations completed in past 24 hours; [3] number of handoffs experienced in past 24 hours; and [4] the number of patients for which the trainee was the primary provider. These questions provide another view of how interns spend their time.

Survey 2 was sent on the alternate days and asked the same first question as Survey 1, as well as the trainee's ratings (too little, just right, too much) for [1] time spent in educational conference and related activities, [2] sense of ownership of patients, [3] work intensity, and [4] continuity of care. Data for these questions are related to satisfaction and complement the iCOMPARE's end-of-year survey (described below) and ACGME survey (described above).

Trainees were entered into an incentive lottery designed so that in each 2-week cycle, one intern and one resident at each of the 63 IM programs were each awarded either a $25 or $100 Amazon gift card if they had completed their survey during that period. After the first cycle, the cycle response rate ranged between 39% and 42%.

<ins>iCOMPARE end-of-year surveys</ins>. All trainees and program directors received an iCOMPARE study-specific survey in May-June 2015 (baseline) and again in May-June 2016 (post-intervention), administered via Lime Survey (https://www.limesurvey.org/). Invitations with personalized links were emailed during May of 2015 and May of 2016 with up to 6 reminders to nonrespondents. At the end of the intervention year, a $2,500 cash incentive was provided to each of the 9 programs with the highest response rates.

The trainee survey was administered to all trainees with only slight differences between versions for interns and PGY2 and higher trainees. The instrument was initially developed for the FIRST trial[26] and included items on trainee satisfaction, experience of duty hours, supervision, fatigue management, and

For peer review only - http://bmjopen.bmj.com/site/about/guidelines.xhtml

resident and patient safety, and ended with the Maslach Burnout Inventory-Human Services Survey (MBI-HSS).[40] The MBI-HSS is a 22-item rating scale assessing three domains: emotional exhaustion (9 items), depersonalization (5 items), and lack of personal accomplishment (8 items). Items are answered on a frequency scale of 0 to 6 where 0 indicates never and 6 indicates every day.

The program director survey was modeled from an earlier survey to program directors [41] and included items on resident and faculty workload, resident morale, continuity, education, patient safety, and program finances and administration.

Aim 3 – Sleep and alertness

Outcomes for the third aim include sleep duration and both subjective and objective measures of alertness among interns at 6 sites randomized to STD and 6 sites randomized to FLEX. At each of these 12 sites, program coordinators recruited interns scheduled to be on general medicine, medical intensive care, cardiology, or cardiac care rotations. Each participating program received $8,000 to cover the costs associated with scheduling interns for data acquisition and managing study equipment. Data collection spanned November 5, 2015 to May 31, 2016. The 12 programs in the Sleep and Alertness Sub-study were selected from across the country and represented programs of various sizes. 457 interns at these 12 programs participated in the consent process; 432 (94.5%) provided written consent, 12 of whom were not eligible due to rotation schedules. Of the remaining 420, 398 (94.8%) provided data for the study and 22 (5.3%) discontinued participation

Participating interns underwent 14-days of continuous measurements of rest and activity via wrist actigraphs (model wGT3X-BT, The Actigraph Corp., Pensacola, FL).[42-44] Interns were instructed to wear the actigraph continuously, even on days off, except during activities that might damage it (e.g., water immersion or contact sports) or that would impede the delivery of clinical care. They were asked

For peer review only - http://bmjopen.bmj.com/site/about/guidelines.xhtml

to remove the actigraph for up to 2 waking hours for recharging on days 1 and 7. Each participating intern received a $10 Amazon gift card for each day for which data were received.

Each morning, the intern completed a brief online survey including the name of the shift that the intern was working; a log reflecting sleep times, sleep quality, and experiences of excessive sleepiness; and the Karolinska Sleepiness Scale.[45] The interns then completed a 3-minute Psychomotor Vigilance Test (PVT-B) [46,47]. All surveys and assessments were completed on a study-supplied Android Smartphone (Samsung Galaxy SIII Neo). The PVT-B is based on simple reaction time to stimuli occurring at random inter-stimulus intervals and is the gold standard for measuring the neurobehavioral effects of acute and chronic sleep loss and circadian misalignment.[48] Actigraphy, survey, and PVT-B data were automatically encrypted and uploaded to a secure server and checked daily for protocol adherence and potential technical issues with the equipment. If problems were detected or no data were received, interns were contacted by the study team to resolve any difficulties interns were having with equipment. Details of scoring are presented in the Supplementary Appendix: Appendix Materials 3: Sleep Actigraphy Scoring and Supplementary Appendix Figure 1 and Figure 2.

Statistical Considerations

Non-inferiority tests will be one-sided and superiority tests will be two-sided. All primary analyses will compare the FLEX and STD treatment groups as randomized, regardless of adherence to the assigned duty hour standards, according to the intention-to-treat principle. Since directors at programs assigned to FLEX have considerable latitude in design of trainee schedules, we expect variation amongst the duty-hour schedules followed in the FLEX group. Protocol-specified secondary analyses addressing the degree of difference between FLEX and STD schedules will be completed. We will also report mortality results adjusting for the clinical condition associated with patient's principal diagnosis as well as demographic

For peer review only - http://bmjopen.bmj.com/site/about/guidelines.xhtml

variables and comorbidities determined using a 6-month look-back period. Similar approaches will be used for the other patient safety outcomes specified in hypotheses H1b-H1e.

The outcome measures for the education hypotheses (H2a-H2d) and the sleep and alertness hypotheses (H3a-H3b) are person-level (intern, trainee, or faculty) measures. If we have repeated measures of an outcome on an individual (e.g., an intern has observations of time spent in direct patient care over multiple shifts, an intern has observations of time spend sleeping over more than 1 day) we will analyze the mean of the repeated measures on the individual, which eliminates within-person correlations. Each outcome will be the response variable in a mixed effects linear or logistic regression model with a random intercept for each program cluster and a single fixed term for treatment group as implemented in SAS (Cary, NC) or STATA (College Station, TX). When pre-trial year data are available, additional analyses will adjust an individual's trial year mean response for the pre-trial year program-level mean response (i.e., a difference of differences analysis). The pre-trial program level mean response must be used since the trainees and faculty providing data in the pre-trial year are not the same trainees and faculty providing data in the trial year, but are at the same IM program.   Specifically we have pre-trial data for Medicare patient level safety analyses, and for some Education outcomes (the ACGME end-of-year surveys, ITE score data from ACP, and the iCOMPARE end-of-year surveys).

For peer review only

Dissemination

Plans for dissemination include submitting results for the various aims to academic meetings and peer-reviewed publications as they become available. The Data and Safety Monitoring Board will comment on manuscripts reporting results related to the primary hypotheses before journal submission.

Discussion

For peer review only - http://bmjopen.bmj.com/site/about/guidelines.xhtml

iCOMPARE is a one-year, cluster-randomized, pragmatic trial designed to evaluate the availability of an alternative resident duty hour schedule for effects on patient safety, resident education, and intern sleep and alertness. iCOMPARE's design is similar to that of the FIRST trial conducted among surgical trainees. What distinguishes both of these trials from prior work investigating effects of duty hour schedules, in addition to their large size, comprehensive approach, and immediate policy relevance, is that they help elevate the standards of evidence applied to graduate medical education policy more generally in the United States and abroad. Although randomized trials provide strong evidence for the causal inference required for good policy, they typically answer narrow questions. Those questions today revolve around the length of shifts within an 80-hour work week. Inside the field of graduate medical education, these issues have been hotly debated, albeit the 80-hours in the U.S. far exceed the limits set for other counties with similar educational systems .[3.4]

iCOMPARE has some limitations. The measurement of patient safety relies predominantly on mortality effects, an important but incomplete measure of relevant clinical outcomes. The measurement of education relies considerably on existing and conventional tests and surveys, and those developed specifically for this study, whereas education is more dimensionalized and, indeed, its own value is perhaps better reflected in changes in how well physicians perform through their careers, unmeasured in this study. Nevertheless, this pragmatic study of health care policy targets measures that are likely to be influential in policy decisions about resident duty hours and available in a time frame when the answers to those questions remain relevant.

Some may wonder why duty hours or shift lengths were not imposed on the intervention group and why, instead, the intervention group merely had permission to use more flexibility in their scheduling. The design of this study means that the potency of the duty hour changes actually implemented by programs may be less extreme than what was permitted by the intervention, adding noise to or

For peer review only - http://bmjopen.bmj.com/site/about/guidelines.xhtml

potentially weakening the overall observed effect. Those concerns are valid to the extent the question is: What happens to patient safety, education, or sleep and alertness when residents are forced to work in certain shifts? But the question at hand is: What happens when programs are allowed flexibility in their scheduling of shifts? The outcome of those policy decisions is, in its implemented state, a product of the flexibility of the rules and the degree to which individual programs take advantage of that flexibility. In some ways, this study design is consistent with that of effectiveness trials of drugs, where the anticipated effect is a product not just of whether one was randomized to the study drug, but also whether one was adherent to the drug. In the real world, adherence is relevant. Context is similarly relevant. The effects we observe in this trial also depend critically on the oversight and supervision provided to interns by more senior residents and on other safety nets built into the environment of hospital practice. While those safety nets potentially blunt observed effects, they take this study beyond the in vitro relevance of laboratory study to a pragmatic context.

With the results of the FIRST trial demonstrating non-inferiority in patient outcomes when a more flexible schedule was available, the ACGME issued new duty hour standards, effective July 2018, that correspond to the intervention arm of iCOMPARE.[49]

Given that surgery and internal medicine are large fields with many residents caring for many patients, it is important to study the duty hour rules in both specialties as surgical and medical training programs differ in structure, process, culture, the kinds of residents they attract, the patients they serve, and the duties of trainees.

Conclusions

There is considerable interest in understanding concerns in the US health care system, such as why US health care is so expensive, why its overall effects on health lag behind those of peer nations in particular with respect to safety, and why its individual effects are so unevenly distributed. In contrast,

For peer review only - http://bmjopen.bmj.com/site/about/guidelines.xhtml

relatively little effort has been devoted to studying how physicians are trained—surprising because although physicians are certainly not the only reason for the concerns, it seems evident that physician training must play an important role in most outcomes related to US health care. In that broad context, iCOMPARE promises narrow but substantive evidence to inform resident duty hour standards in internal medicine and signals new interest in methodologically stronger research in medical education.

For peer review only

For peer review only - http://bmjopen.bmj.com/site/about/guidelines.xhtml

References

1. Friedman RC, Bigger JT, Kornfeld DS. The intern and sleep loss. *N Engl J Med* 1971;285(4):201–3.
2. Asch DA, Parker RM. The Libby Zion case: One step forward or two steps backward? *N Engl J Med* 1988;318:771-5.
3. Resident Duty Hours: Enhancing Sleep, Supervision and Safety, Appendix C: International Experiences Limiting Duty Hours. Pp 339-361, National Academies Press: Washington, DC. 2009
4. Temple J. Resident duty hours around the globe: Where are we now? *BMC Med Educ* 2014;14(u 1):S8
5. Ulmer C, Wolman DM, Johns MME, eds. Resident Duty Hours: Enhancing Sleep, Supervision, and Safety. National Academies Press: Washington, DC. 2009.
6. Landrigan CP, Rothschild JM, Cronin JW, et al. Effects of reducing interns' work hours on serious medical errors in intensive care units. *N Engl J Med* 2004;351(18):1838-48.
7. Asch DA, Bilimoria KY, Desai SV. Resident duty hours and medical education policy — raising the evidence bar. *N Engl J Med* 2017;376:1704-6.
8. Petersen LA, Brennan TA, O'Neil AC, Cook EF, Lee TH. Does housestaff discontinuity of care increase the risk for preventable adverse events? *Ann Intern Med* 1994;121(11):866-72.
9. Kitch BT, Cooper JB, Zapol WM, et al. Handoffs causing patient harm: a survey of medical and surgical house staff. *Jt Comm J Qual Patient Saf* 2008;34(10):563-70.
10. Volpp KG, Rosen AK, Rosenbaum PR, et al. Mortality among hospitalized Medicare beneficiaries in the first 2 years following ACGME resident duty hour reform. *JAMA* 2007;298:975-83.
11. Volpp KG, Rosen AK, Rosenbaum PR, et al. Mortality among patients in VA hospitals in the first 2 years following ACGME resident duty hour reform. *JAMA* 2007;298:984-92.
12. Prasad M, Iwashyna TJ, Christie JD, et al. Effect of work-hours regulations on intensive care unit

For peer review only - http://bmjopen.bmj.com/site/about/guidelines.xhtml

mortality in United States teaching hospitals. *Crit Care Med* 2009; 37:2564-69.

13. Rosen AK, Loveland SA, Romano PS, et al. Effects of resident duty hour reform on surgical and procedural patient safety indicators among hospitalized Veterans Health Administration and Medicare patients. *Med Care* 2009; 47:723-31.
14. Silber JH, Rosenbaum PR, Rosen AK, et al. Prolonged hospital stay and the resident duty hour rules of 2003. *Med Care* 2009; 47:1191-200.
15. Volpp KG, Rosen AK, Rosenbaum PR, et al. Did duty hour reform lead to better outcomes among the highest risk patients? *J Gen Intern Med* 2009; 24:1149-55.
16. Press MJ, Silber JH, Rosen AK, et al. The impact of resident duty hour reform on hospital readmission rates among Medicare beneficiaries. *J Gen Intern Med* 2011; 26:405-11.
17. Navathe AS, Silber JH, Small DS, et al. Teaching hospital financial status and patient outcomes following ACGME duty hour reform. *Health Serv Res* 2013; 48:476-98.
18. Volpp KG, Small DS, Romano PS, et al. Teaching hospital five-year mortality trends in the wake of duty hour reforms. *J Gen Intern Med* 2013; 28:1048-55.
19. Patel MS, Volpp KG, Small DS, et al. Association of the 2011 ACGME resident duty hour reforms with mortality and readmissions among hospitalized Medicare patients. *JAMA* 2014; 312:2364-73.
20. Block L, Habicht R, Wu AW, et al. In the wake of the 2003 and 2011 duty hours regulations, how do internal medicine interns spend their time? *J Gen Intern Med* 2013;28(8):1042-47.
21. Desai SV, Feldman L, Brown L, et al. Effect of the 2011 vs 2003 duty hour regulation-compliant models on sleep duration, trainee education, and continuity of patient care among internal medicine house staff: a randomized trial. *JAMA Intern Med* 2013;173(8):649-55.
22. Sen S, Kranzler HR, Didwania AK, et al. Effects of the 2011 duty hour reforms on interns and their patients: a prospective longitudinal cohort study. *JAMA Intern Med* 2013;173(8):657-63.

For peer review only - http://bmjopen.bmj.com/site/about/guidelines.xhtml

23. Silber JH, Romano PS, Itani KM, et al. Assessing the effects of the 2003 resident duty hours reform on internal medicine board scores. *Acad Med* 2014; 89:644-51.

24. Drolet BC, Christopher DA, Fischer SA. Residents' response to duty-hour regulations--a follow-up national survey. *N Engl J Med* 2012;366(24):e35.

25. Drolet BC, Khokhar MT, Fischer SA. The 2011 duty-hour requirements--a survey of residency program directors. *N Engl J Med* 2013;368(8):694-97.

26. Drolet BC, Spalluto LB, Fischer SA. Residents' perspectives on ACGME regulation of supervision and duty hours--a national survey. *N Engl J Med* 2010;363(23):e34.

27. Bilimoria KY, Chung JW, Hedges LV, et al. Development of the flexibility in duty hour requirements for surgical trainees (FIRST) trial protocol: A national cluster-randomized trial of resident duty hour policies. *JAMA Surg* 2016;151:273-81.

28. Bilimoria KY, Chung JW, Hedges LV, et al. National cluster-randomized trial of duty-hour flexibility in surgical training. *N Engl J Med* 2016; 374:713-27.

29. Fletcher KE, Visotcky AM, Slagle JM, et al. The composition of intern work while on call. *J Gen Intern Med* 2012;27(11):1432-7.

30. Block L, Habicht R, Wu AW, et al. In the wake of the 2003 and 2011 duty hours regulations, how do internal medicine interns spend their time? *J Gen Intern Med* 2013;28:1042-7.

31. Volpp KG, Shea JA, Small DS, et al. Effect of a protected sleep period on hours slept during extended overnight in-hospital duty hours among medical interns. *JAMA* 2012;308(21):2208-17.

32. Drye E, Normand SL, Wang Y, et al. Comparison of hospital risk-standardized mortality rates calculated by using in-hospital and 30-day models: implications for hospital profiling. *Ann Intern Med* 2012;156:19-26.

33. Meehan TP, Fine MJ, Krumholz HM, et al. Quality of care, process, and outcomes in elderly patients with pneumonia. *JAMA* 1997;278:2080-4.

For peer review only - http://bmjopen.bmj.com/site/about/guidelines.xhtml

34. Silber JH, Rosenbaum PR, Even-Shoshan O, et al. Length of stay, conditional length of stay, and prolonged stay in pediatric asthma. *Health Serv Res* 2003;38:867-86.

35. Silber JH, Rosenbaum PR, Kelz RR, et al. Medical and financial risks associated with surgery in the elderly obese. *Ann Surg* 2012;256:79-86.

36. Silber JH, Rosenbaum PR, Koziol LF, Sutaria N, Marsh RR, Even-Shoshan O. Conditional length of stay. *Health Serv Res* 1999;34:349-63.

37. Krumholz HM, Lin Z, Keenan PS, et al. Relationship between hospital readmission and mortality rates for patients hospitalized with acute myocardial infarction, heart failure, or pneumonia. *JAMA* 2013;309:587-93.

38. Iezzoni LI, Daley J, Heeren T, et al. Using administrative data to screen hospitals for high complication rates. *Inquiry* 1994;31:40-55.

39. McCarthy EP, Iezzoni LI, Davis RB, et al. Does clinical evidence support ICD-9-CM diagnosis coding of complications? *Med Care* 2000;38:868-76.

40. Maslach C, Jackson SE, Leiter MP. Maslach Burnout Inventory. 3rd Ed. Palo Alto: Consulting Psychologists Press. 1996.

41. Shea JA, Willett LL, Borman KR, et al. Anticipated consequences of the 2011 duty hour standards: A view from surgery and medicine program directors. *Acad Med* 2012:87;895-903.

42. Ancoli-Israel S, Cole R, Alessi C, et al. The role of actigraphy in the study of sleep and circadian rhythms. *Sleep* 2003;26(3):342-92

43. Malmberg B, Kecklund G, Karlson B, et al. Sleep and recovery in physicians on night call: a longitudinal field study. *BMC Health Serv Res* 2010;10(239).

44. Basner M, Dinges DF, Shea JA, et al. Sleep and Alertness in Medical Interns and Residents: An observational study on the role of extended shifts. *Sleep* 2017;40(4). doi: 10.1093/sleep/zsx027

For peer review only - http://bmjopen.bmj.com/site/about/guidelines.xhtml

45. Akerstedt T, Gillberg M. Subjective and objective sleepiness in the active individual. *Int J Neurosci* 1990;52(1-2):29-37.

46. Basner M, Mollicone DJ, Dinges DF. Validity and sensitivity of a brief Psychomotor Vigilance Test (PVT-B) to total and partial sleep deprivation. *Acta Astronautica* 2011;69:949-59.

47. Basner M, Dinges DF. Maximizing sensitivity of the Psychomotor Vigilance Test (PVT) to sleep loss. *Sleep* 2011;34(5):581-91.

48. Dinges DF, Maislin G, Hanowski RJ, et al. Commercial Motor Vehicle (CMV) Driver Restart Study: Final Report. U.S. DoT; Federal Motor Carrier Safety Administration; Office of Analysis, Research, and Technology. 2016.

49. The ACGME Common Program Requirements Section VI revisions from TJ Nasca, MD. ACGME, 2017. (Accessed August 15, 2017, at http://www.acgme.org/Portals/0/PDFs/Nasca-Community/Section-VI-Memo-3-10-17.pdf.)

For peer review only

For peer review only - http://bmjopen.bmj.com/site/about/guidelines.xhtml

Authors' contributions

Judy A. Shea, PhD is a co-investigator. She took the lead in drafting this paper and incorporating all co-authors' suggestions on subsequent drafts. She also worked closely with Drs. Desai and Bellini to design and refine the aims and methods related to the education aim.

Jeffrey H. Silber, MD, PHD took the lead on writing the protocol for the patient safety component of the protocol. He reviewed and commented on multiple versions of this manuscript.

Sanjay V. Desai, MD worked with Drs. Shea and Bellini to develop the details for the education aim and outcomes. He reviewed and commented on multiple versions of this manuscript.

David F Dinges, PhD, took the lead and worked with Dr. Basner to develop the details for the sleep and alertness aim and outcomes. He reviewed and commented on multiple versions of this manuscript.

Lisa M.Bellini, MD worked with Dr. Shea and Desai to develop the details for the education aim and outcomes. She reviewed and commented on multiple versions of this manuscript.

James Tonascia, PhD took the lead and worked with Alice L Sternberg, ScM, Dylan S. Small, PhD and David M Shade, JD to develop the sample size calculations and analytic plan for all study aims. They each reviewed and commented on multiple versions of this manuscript.

Joel T. Katz, MD participated in design of the direct observation portion of the protocol and commented on multiple versions of the manuscript.

Mathias Basner, MD, PhD worked with Dr. Dinges to develop the details for the sleep and alertness aim and outcomes. He reviewed and commented on multiple versions of this manuscript.

For peer review only - http://bmjopen.bmj.com/site/about/guidelines.xhtml

Krisda H. Chaiyachati, MD, MPH took the lead in developing the details for the direct observation portion of the protocol. He reviewed and commented on multiple versions of this manuscript.

Orit Even-Shoshan, MS worked with Dr. Silber to design the patient safety portion of the protocol.

David W. Bates, MD participated in design of the direct observation portion of the protocol and commented on multiple versions of the manuscript.

Kevin G. Volpp, MD, PhD worked on the overall development of the protocol and commented on versions of the manuscript.

David A. Asch, MD, MBA is the principal investigator for the iCOMPARE protocol and has been instrumental in all phases of protocol design and study implementation.

The iCOMPARE Research Group, comprised of all participating program directors and project staff, was instrumental in recruitment and data collection.

This work is supported by the National Heart, Lung, and Blood Institute (NHLBI), project number U01HL125388 to Dr Asch and grant number U01HL126088 to Dr Tonascia. This paper is subject to NIH Public Access Policy

Trial Registration: clinicaltrials.gov Identifier: NCT02274818.

The authors report no competing interests.

For peer review only

For peer review only - http://bmjopen.bmj.com/site/about/guidelines.xhtml

Table 1 Study Aims and Hypotheses

Primary Hypothesis:

Specific Aim 1: Examine patient safety and costs of care under STD and FLEX duty hour schedules.

H1a: *(Primary hypothesis) 30-day patient mortality under FLEX will not exceed (will not be inferior to) mortality under STD.*

Secondary Hypotheses:

H1b: *7-day and 30-day hospital readmission rates under FLEX will not exceed (will not be inferior to) the rates under STD.*

H1c: *Complication rates, defined by selected AHRQ Patient Safety Indicators, under Flex will not exceed (will not be inferior to) complication rates under STD.*

H1d: *The rate of prolonged length of stay under FLEX will not exceed (will not be inferior to) the rate of prolonged length of stay under STD.*

H1e: *Total costs and Medicare payments, under FLEX will not exceed (will not be inferior to) overall costs under STD.*

Specific Aim 2: Examine the quality of education under STD and FLEX duty hour schedules.

Secondary Hypotheses:

H2a: *Interns in FLEX will spend greater time in direct patient care and education compared to interns in STD.*

H2b: *Trainees in FLEX will report greater satisfaction with their educational experience (greater ownership, greater continuity and lower burnout) than trainees in STD.*

H2c: *Faculty in FLEX will report greater satisfaction with their clinical teaching experiences and greater perceptions of safety, teamwork and supervision than faculty in STD.*

H2d: *Standardized test scores for interns in Flex will not be less than (inferior to) those for interns in STD.*

For peer review only - http://bmjopen.bmj.com/site/about/guidelines.xhtml

Specific Aim 3: Examine intern sleep time and alertness under STD and FLEX duty hour schedules.

<u>Secondary Hypotheses</u>:

H3a: Average daily sleep obtained by interns in FLEX will not be less than (will not be inferior to) that of interns in STD, as determined by a 14-day period of sleep monitoring using actigraphy and daily sleep diaries.

H3b: Interns in FLEX will not have (will not be inferior to) greater average subjective sleepiness via the Karolinska Sleepiness Scale (KSS)(24), or lower average behavioral alertness via the Psychomotor Vigilance Test (PVT)(25) than interns in STD, as determined by a 14-day period of morning sleepiness-alertness monitoring.

For peer review only

For peer review only - http://bmjopen.bmj.com/site/about/guidelines.xhtml

Table 2. Overview of Usual Care and Intervention Arm Duty Hour Requirements

**Requirement**	**Usual Care (STD; current duty hour standards)**	**Intervention (FLEX; flexible duty hour standards)**
80 hours per week (averaged over 4 weeks)	No change	No change
1 day off per week (averaged over 4 weeks)	No change	No change
In-house call no more than every 3rd night (averaged over 4-weeks)	No change	No change
PGY-1 resident duty hour periods must not exceed 16 hours	No change	Eliminated
PGY-2 and above must not work more than 24 hours with an additional 4 hours permitted for transitions in care	No change	Eliminated
Residents must have 14 hours off after 24 hours in-house duty and at least 8-10 hours off after a regular shift	No change	Eliminated

Adapted from Bilimoria KY, Chung JW, Hedges LV, et al. Development of the flexibility in duty hour requirements for surgical trainees (FIRST) trial protocol. JAMA Surg. 2016;151:273-81.

For peer review only

For peer review only - http://bmjopen.bmj.com/site/about/guidelines.xhtml

Table 3: Operationalization of Primary Education Measures

Hypothesis	Primary Data Source	Who	Definition
2a – time in direct patient care and education	Time-Motion sub-study	PGY1	percent of observed shift minutes spent in each activity
2b – Trainee satisfaction	ACGME year-end trainee survey	PGY1-3	a single item on the ACGME resident survey re: appropriate balance for education with response options of never, rarely, sometimes, often, very often
2c – Program director satisfaction	ACGME core faculty survey	Core faculty	a single item on the ACGME faculty survey that asked about residents'/fellows' workload exceeding the capacity to do the work with response options of never, rarely, sometimes, often, very often
2d- Standardized test scores	ACP in-training examination scores	PGY1	percent of questions answered correctly

PGY = post graduate year, ACGME=Accreditation Council for Graduate Medical Education, ACP=American College of Physicians

For peer review only - http://bmjopen.bmj.com/site/about/guidelines.xhtml

Figure 1 Legend: Consort Diagram

Figure 2 Legend: iCOMPARE Trial Timeline.

For peer review only

For peer review only - http://bmjopen.bmj.com/site/about/guidelines.xhtml

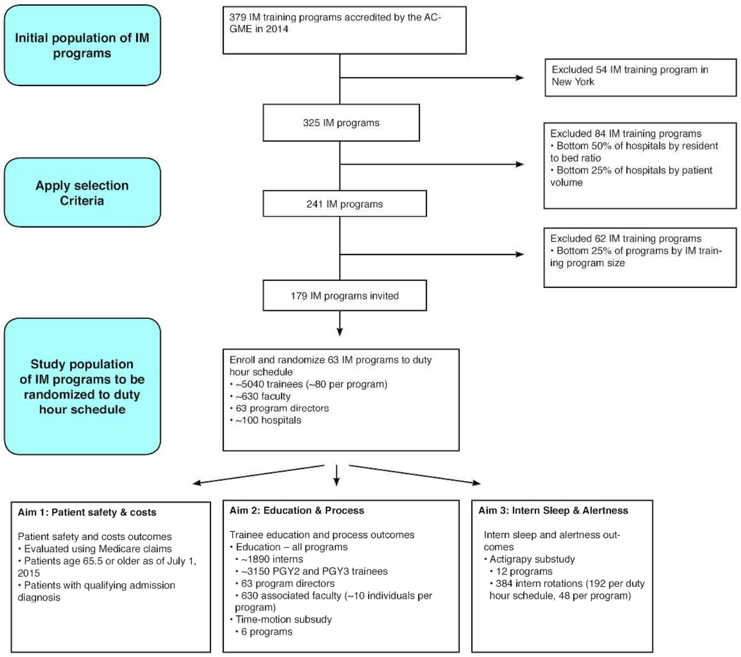

Consort Diagram

279x215mm (300 x 300 DPI)

w only

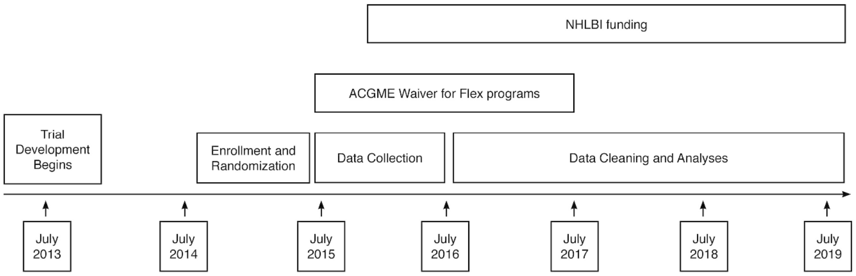

Figure 2 Legend: iCOMPARE Trial Timeline.

279x215mm (300 x 300 DPI)

Development of the Individualized Comparative Effectiveness of Models Optimizing Patient Safety and Resident Education (iCOMPARE) Trial: A Protocol Summary of a National Cluster-Randomized Trial of Resident Duty Hour Policies in Internal Medicine

Judy A. Shea, PhD,[1] Jeffrey H. Silber, MD, PhD,[2] Sanjay V. Desai, MD,[3] David F. Dinges PhD,[4] Lisa M. Bellini, MD,[1] James Tonascia, PhD,[5] Alice L. Sternberg, ScM,[6] Dylan S. Small, PhD,[7] David M. Shade, JD,[6] Joel T. Katz, MD,[8] Mathias Basner, MD, PhD,[4] Krisda H. Chaiyachati, MD, MPH,[1,10] Orit Even-Shoshan, MS,[2] David W. Bates, MD, MSc,[8] Kevin G. Volpp, MD, PhD,[9,10] David A. Asch, MD, MBA[1,10] and the iCOMPARE Research Group

For peer review only

Supplementary Appendix

Table of Contents

Appendix Materials 1. Rationale and ICD-9 Codes for Principal Diagnoses Qualifying Hospital/Patient for Inclusion in iCOMPARE Randomization/Analysis

Appendix Materials 2. Details for Secondary Outcomes for Patient Safety Aim: Prolonged Length of Stay, Readmissions, Complications, and Costs

Appendix Materials 3. Sleep Actigraphy Scoring

Appendix Figure 1. Overview of Actigraphy and PVT Scoring and Review Process

Appendix Figure 2. Sleep Scoring Matrix

For peer review only - http://bmjopen.bmj.com/site/about/guidelines.xhtml

Appendix Materials 1. Rationale and ICD-9 Codes for Principal Diagnoses Qualifying Hospital/Patient for Inclusion in iCOMPARE Randomization/Analysis

The original ICD-9 code list was created by searching for relevant codes for each medical condition that were found to be associated with high mortality rates in our preliminary 2008 data. The outcomes team reviewed the code list and expanded the code list in two ways:

- We consulted the official ICD-9 code book and looked for additional high-volume codes that were related to the medical conditions of interest. The list of proposed expansion codes was reviewed and approved by the coding consultant to the study, Dr. Patrick Romano of the University of California, Davis.
- To account for the presence of ICD-10 codes on claims with discharge dates beginning October 1, 2015, we utilized the General Equivalence Mappings (GEMs) made available by the Centers for Medicare and Medicaid Services, which provide the closest possible approximation of a translation between the ICD-9 and ICD-10 code systems. Our goal was to translate each ICD-10 code back to its most closely equivalent ICD-9 code, and our review of the crosswalk caused us to pick up a small number of additional ICD-9 codes. As with the original code list expansion, these proposed additional codes were reviewed and approved by our coding consultant to the study.

Pneumonia:

481	Pneumococcal Pneumonia [Streptococcus Pneumoniae Pneumonia]
482	Other Bacterial Pneumonia
482.1	Pneumonia Due to Pseudomonas
482.2	Pneumonia Due to Hemophilus Influenzae (H. Influenzae)
482.3	Pneumonia Due to Streptococcus

For peer review only - http://bmjopen.bmj.com/site/about/guidelines.xhtml

482.31	Pneumonia Due to Streptococcus Group A
482.32	Pneumonia Due to Streptococcus Group B
482.39	Pneumonia Due to Other Streptococcus
482.41	Methicillin Susceptible Pneumonia Due to Staphylococcus Aureus
482.42	Methicillin Resistant Pneumonia Due to Staphylococcus Aureus
482.49	Other Staphylococcus Pneumonia
482.82	Pneumonia Due to Escherichia Coli [E.Coli]
482.83	Pneumonia Due to Other Gram-Negative Bacteria
482.84	Pneumonia Due to Legionnaires' Disease
482.89	Pneumonia Due to Other Specified Bacteria
482.4	Pneumonia Due to Staphylococcus
482.9	Bacterial Pneumonia Unspecified
483	Pneumonia Due to Other Specified Organism
483.1	Pneumonia Due to Chlamydia
483.8	Pneumonia Due to Other Specified Organism
485	Bronchopneumonia Organism Unspecified
486	Pneumonia Organism Unspecified

Stroke:

430	Subarachnoid Hemorrhage
431	Intracerebral Hemorrhage
432.0	Nontraumatic Extradural Hemorrhage
432.1	Subdural Hemorrhage
432.9	Unspecified Intracranial Hemorrhage

For peer review only - http://bmjopen.bmj.com/site/about/guidelines.xhtml

433.01	Occlusion and Stenosis of Basilar Artery with Cerebral Infarction
433.11	Occlusion and Stenosis of Carotid Artery with Cerebral Infarction
433.21	Occlusion and Stenosis of Vertebral Artery with Cerebral Infarction
433.31	Occlusion and Stenosis of Multiple and Bilateral Precerebral Arteries with Cerebral Infarction
433.81	Occlusion and Stenosis of Other Specified Precerebral Artery with Cerebral Infarction
433.91	Occlusion and Stenosis of Unspecified Precerebral Artery with Cerebral Infarction
434.01	Cerebral Thrombosis with Cerebral Infarction
434.11	Cerebral Embolism with Cerebral Infarction
434.91	Cerebral Artery Occlusion Unspecified with Cerebral Infarction
436	Acute but Ill-Defined Cerebrovascular Disease

For peer review only

For peer review only - http://bmjopen.bmj.com/site/about/guidelines.xhtml

Acute Myocardial Infarction:

410.01 Acute Myocardial Infarction of Anterolateral Wall Initial Episode of Care

410.11 Acute Myocardial Infarction of Other Anterior Wall Initial Episode of Care

410.21 Acute Myocardial Infarction of Inferolateral Wall Initial Episode of Care

410.31 Acute Myocardial Infarction of Inferoposterior Wall Initial Episode of Care

410.41 Acute Myocardial Infarction of Other Inferior Wall Initial Episode of Care

410.51 Acute Myocardial Infarction of Other Lateral Wall Initial Episode of Care

410.61 True Posterior Wall Infarction Initial Episode of Care

410.71 Subendocardial Infarction Initial Episode of Care

410.81 Acute Myocardial Infarction of Other Specified Sites Initial Episode of Care

410.91 Acute Myocardial Infarction of Unspecified Site Initial Episode of Care

Gastrointestinal Hemmorhage:

456.0 Esophageal Varices with Bleeding

530.7 Gastroesophageal Laceration-Hemorrhage Syndrome

530.82 Esophageal Hemorrhage

531.00 Acute Gastric Ulcer with Hemorrhage Without Obstruction

531.01 Acute Gastric Ulcer with Hemorrhage with Obstruction

531.20 Acute Gastric Ulcer with Hemorrhage and Perforation Without Obstruction

531.21 Acute Gastric Ulcer with Hemorrhage and Perforation with Obstruction

531.40 Chronic or Unspecified Gastric Ulcer with Hemorrhage Without Obstruction

531.41 Chronic or Unspecified Gastric Ulcer with Hemorrhage with Obstruction

531.60 Chronic or Unspecified Gastric Ulcer with Hemorrhage and Perforation Without Obstruction

For peer review only - http://bmjopen.bmj.com/site/about/guidelines.xhtml

531.61	Chronic or Unspecified Gastric Ulcer with Hemorrhage and Perforation with Obstruction
532.00	Acute Duodenal Ulcer with Hemorrhage Without Obstruction
532.01	Acute Duodenal Ulcer with Hemorrhage with Obstruction
532.20	Acute Duodenal Ulcer with Hemorrhage and Perforation Without Obstruction
532.21	Acute Duodenal Ulcer with Hemorrhage and Perforation with Obstruction
532.40	Chronic or Unspecified Duodenal Ulcer with Hemorrhage Without Obstruction
532.41	Chronic or Unspecified Duodenal Ulcer with Hemorrhage with Obstruction
532.60	Chronic or Unspecified Duodenal Ulcer with Hemorrhage and Perforation Without Obstruction
532.61	Chronic or Unspecified Duodenal Ulcer with Hemorrhage and Perforation with Obstruction
533.00	Acute Peptic Ulcer of Unspecified Site with Hemorrhage Without Obstruction
533.01	Acute Peptic Ulcer of Unspecified Site with Hemorrhage with Obstruction
533.20	Acute Peptic Ulcer of Unspecified Site with Hemorrhage and Perforation Without Obstruction
533.21	Acute Peptic Ulcer of Unspecified Site with Hemorrhage and Perforation with Obstruction
533.40	Chronic or Unspecified Peptic Ulcer of Unspecified Site with Hemorrhage Without Obstruction
533.41	Chronic or Unspecified Peptic Ulcer of Unspecified Site with Hemorrhage with Obstruction
533.60	Chronic or Unspecified Peptic Ulcer of Unspecified Site with Hemorrhage and Perforation Without Obstruction

For peer review only - http://bmjopen.bmj.com/site/about/guidelines.xhtml

533.61	Chronic or Unspecified Peptic Ulcer of Unspecified Site with Hemorrhage and Perforation with Obstruction
534.00	Acute Gastrojejunal Ulcer with Hemorrhage Without Obstruction
534.01	Acute Gastrojejunal Ulcer with Hemorrhage with Obstruction
534.20	Acute Gastrojejunal Ulcer with Hemorrhage and Perforation Without Obstruction
534.21	Acute Gastrojejunal Ulcer with Hemorrhage and Perforation with Obstruction
534.40	Chronic or Unspecified Gastrojejunal Ulcer with Hemorrhage Without Obstruction
534.41	Chronic or Unspecified Gastrojejunal Ulcer with Hemorrhage with Obstruction
534.60	Chronic or Unspecified Gastrojejunal Ulcer with Hemorrhage and Perforation Without Obstruction
534.61	Chronic or Unspecified Gastrojejunal Ulcer with Hemorrhage and Perforation with Obstruction
535.01	Acute Gastritis with Hemorrhage
535.11	Atrophic Gastritis with Hemorrhage
535.21	Gastric Mucosal Hypertrophy with Hemorrhage
535.31	Alcoholic Gastritis with Hemorrhage
535.41	Other Specified Gastritis with Hemorrhage
535.51	Unspecified Gastritis and Gastroduodenitis with Hemorrhage
535.61	Duodenitis with Hemorrhage
537.83	Angiodysplasia of Stomach and Duodenum with Hemorrhage
562.02	Diverticulosis of Small Intestine with Hemorrhage
562.03	Diverticulitis of Small Intestine with Hemorrhage
562.12	Diverticulosis of Colon with Hemorrhage
562.13	Diverticulitis of Colon with Hemorrhage

For peer review only

569.3	Hemorrhage of Rectum and Anus
569.85	Angiodysplasia of Intestine with Hemorrhage
578.0	Hematemesis
578.1	Blood In Stool
578.9	Hemorrhage of Gastrointestinal Tract Unspecified

Heart Failure:

398.91	Rheumatic Heart Failure (Congestive)
402.01	Malignant Hypertensive Heart Disease with Heart Failure
402.11	Benign Hypertensive Heart Disease with Heart Failure
402.91	Unspecified Hypertensive Heart Disease with Heart Failure
404.01	Hypertensive Heart and Chronic Kidney Disease Malignant with Heart Failure with Chronic Kidney Disease Stage I Through Stage Iv or Unspecified
404.03	Hypertensive Heart and Chronic Kidney Disease Malignant with Heart Failure with Chronic Kidney Disease Stage V or End Stage Renal Disease
404.11	Hypertensive Heart and Chronic Kidney Disease Benign with Heart Failure with Chronic Kidney Disease Stage I Through Stage Iv or Unspecified
404.13	Hypertensive Heart and Chronic Kidney Disease Benign with Heart Failure with Chronic Kidney Disease Stage V or End Stage Renal Disease
404.91	Hypertensive Heart and Chronic Kidney Disease Unspecified with Heart Failure with Chronic Kidney Disease Stage I Through Stage Iv or Unspecified
404.93	Hypertensive Heart and Chronic Kidney Disease Unspecified with Heart Failure with Chronic Kidney Disease Stage V or End Stage Renal Disease
428	Heart Failure

For peer review only

For peer review only - http://bmjopen.bmj.com/site/about/guidelines.xhtml

428.0	Congestive Heart Failure Unspecified
428.1	Left Heart Failure
428.20	Unspecified Systolic Heart Failure
428.21	Acute Systolic Heart Failure
428.22	Chronic Systolic Heart Failure
428.23	Acute on Chronic Systolic Heart Failure
428.30	Unspecified Diastolic Heart Failure
428.31	Acute Diastolic Heart Failure
428.32	Chronic Diastolic Heart Failure
428.33	Acute on Chronic Diastolic Heart Failure
428.40	Unspecified Combined Systolic and Diastolic Heart Failure
428.41	Acute Combined Systolic and Diastolic Heart Failure
428.42	Chronic Combined Systolic and Diastolic Heart Failure
428.43	Acute on Chronic Combined Systolic and Diastolic Heart Failure
428.9	Heart Failure Unspecified

Septicemia:

038	Septicemia
038.0	Streptococcal Septicemia
038.1	Staphylococcal Septicemia
038.9	Unspecified Septicemia

Kidney Failure:

584	Acute Kidney Failure

For peer review only - http://bmjopen.bmj.com/site/about/guidelines.xhtml

584.9	Acute Kidney Failure Unspecified

Cardiac Disease:

427.31	Atrial Fibrillation
427.41	Ventricular Fibrillation
427.5	Cardiac Arrest

Chronic Obstructive Pulmonary Disease:

490	Bronchitis Not Specified as Acute or Chronic
491.21	Obstructive Chronic Bronchitis with (Acute) Exacerbation
491.22	Obstructive Chronic Bronchitis with Acute Bronchitis

Pancreatitis:

577.0	Acute Pancreatitis
577.9	Unspecified Disease of Pancreas

Acute Respiratory Failure:

518.81	Acute Respiratory Failure
518.84	Acute and Chronic Respiratory Failure
518.89	Other Diseases of Lung Not Elsewhere Classified
519.11	Acute Bronchospasm

Chest Pain:

786.5	Chest Pain

For peer review only

For peer review only - http://bmjopen.bmj.com/site/about/guidelines.xhtml

786.59	Other Chest Pain

Cellulitis:

682	Other Cellulitis and Abscess
682.6	Cellulitis and Abscess of Leg Except Foot

Coronary Atherosclerosis:

414.01	Coronary Atherosclerosis of Native Coronary Artery
414.1	Aneurysm and Dissection of Heart

Pulmonary Embolism:

415	Acute Pulmonary Heart Disease
415.12	Septic Pulmonary Embolism
415.19	Other Pulmonary Embolism and Infarction

Syncope:

780.0	Alteration of Consciousness
780.2	Syncope and Collapse
780.3	Convulsions
780.01	Coma

Intestinal Infection:

008	Intestinal Infections Due to Other Organisms
008.45	Intestinal Infection Due to Clostridium Difficile

For peer review only

For peer review only - http://bmjopen.bmj.com/site/about/guidelines.xhtml

008.49	Intestinal Infection Due to Other Organisms

Obstructive Asthma:

493.9	Asthma Unspecified
493.22	Chronic Obstructive Asthma with (Acute) Exacerbation

Bronchitis:

494.1	Bronchiectasis with Acute Exacerbation

For peer review only

Appendix Materials 2: Details for Secondary Outcomes for Patient Safety Aim: Prolonged Length of Stay, Readmissions, Complications, and Costs

All patient safety measures are based on information made available to researchers by the Centers for Medicare and Medicaid Services (CMS). Health services researchers have conducted research using Medicare claims files for over two decades. This information is uniformly recorded for all Medicare beneficiaries. CMS releases to researchers the de-identified claim files for hospitals' and physicians' services after validating them through an adjudicated process. All the patient safety outcomes are calculated by the outcomes team based on information from the claims data.

The reliability of the data used for calculating patient safety outcomes for mortality, readmissions, and length of stay (LOS) is excellent. The reliability of the data used to calculate complications, costs, and payments is also high, but lower than of the data used in calculating the 30- day mortality, readmissions and LOS outcomes, due to variation across hospitals in the number and may vary across hospitals due in the number of diagnostic and procedure codes recorded in the claim. However, due to the randomized nature of the study, we would expect there to be no difference in these outcomes between the two study arms (FLEX and STD). References regarding the reliability of the various patient safety measures are provided below.

In addition to mortality, the following outcomes measures were collected:

- Readmission: This calculation is based on the admission and discharge dates in the Medicare claims.
- Length of Stay and Prolonged Length of Stay: Calculated based on the admission and discharge dates of the index claim.
- Complications: Calculated using the Agency for Healthcare Research and Quality Patient Safety Indicators (see below).

For peer review only - http://bmjopen.bmj.com/site/about/guidelines.xhtml

- Costs: Calculated using inpatient, revenue center, and Part B claims. We utilize a resource costing method (more details are provided below) to estimate the costs associated with accommodations (general floor and intensive care unit), the operating room, post-discharge emergency room visits, and other services indicated by the presence of Current Procedural Terminology codes (which are translated to Relative Value Units).
- Payments: Calculated using the payment variables that appear in the inpatient, outpatient, and Part B claims. The total amounts paid by Medicare, the beneficiary, and the primary payer are summed. Year-based adjustments for inflation are applied to the payment figures.

The Patient Safety Indicators were calculated by the research team using SAS programs provided by the Agency for Healthcare Research and Quality, which were run on the Medicare claims. Some of the Patient Safety Indicators that were considered "postoperative" or "perioperative" were modified for use with the study's population of medical patients. For these Patient Safety Indicators, the portion of the code that required the patient to have had surgery was deleted.

The following Patient Safety Indicators were used:

- PSI 03 - Pressure ulcer rate
- PSI 06 - Iatrogenic pneumothorax rate
- PSI 07 - Central venous catheter-related blood stream infection rate
- PSI 08 - Postoperative hip fracture rate
- PSI 09 - Perioperative hemorrhage or hematoma rate
- PSI 10 - Postoperative physiologic and metabolic derangement rate
- PSI 11 - Postoperative respiratory failure rate
- PSI 12 - Perioperative pulmonary embolism or deep vein thrombosis rate
- PSI 13 - Postoperative sepsis rate

For peer review only

For peer review only - http://bmjopen.bmj.com/site/about/guidelines.xhtml

References describing the validity of the claims data used to calculate the patient safety outcomes are in the main manuscript and repeated here:

Readmissions:

- Patel MS, Volpp KG, Small DS, et al. Association of the 2011 ACGME resident duty hour reforms with mortality and readmissions among hospitalized Medicare patients. JAMA 2014;312:2364-73.
- Krumholz HM, Lin Z, Keenan PS, et al. Relationship between hospital readmission and mortality rates for patients hospitalized with acute myocardial infarction, heart failure, or pneumonia. JAMA 2013;309:587-93.

Length of Stay and Prolonged Length of Stay:

- Silber JH, Rosenbaum PR, Even-Shoshan O, et al. Length of stay, conditional length of stay, and prolonged stay in pediatric asthma. Health Serv Res 2003;38:867-86.
- Silber JH, Rosenbaum PR, Kelz RR, et al. Medical and financial risks associated with surgery in the elderly obese. Ann Surg 2012;256:79-86.
- Silber JH, Rosenbaum PR, Koziol LF, et al. Conditional length of stay. Health Serv Res 1999;34:349-63.
- Silber JH, Rosenbaum PR, Rosen AK, et al. Prolonged hospital stay and the resident duty hour rules of 2003. Med Care 2009;47:1191-200.

Patient Safety Indicators (description of PSIs):

- Rosen AK, Loveland SA, Romano PS, et al. Effects of resident duty hour reform on surgical and procedural patient safety indicators among hospitalized Veterans Health Administration and Medicare patients. Med Care 2009;47:723-31.

For peer review only - http://bmjopen.bmj.com/site/about/guidelines.xhtml

Patient Safety Indicators (validity):

- Romano PS, Geppert JJ, Davies S, et al. A national profile of patient safety in US hospitals. Health Aff. 2003;22:154–166.
- Rosen AK, Rivard P, Zhao S, et al. Evaluating the patient safety indicators: how well do they perform on Veterans Health Administration data? Med Care. 2005;43:873–884.
- Zhan C, Miller MR. Excess length of stay, charges, and mortality attributable to medical injuries during hospitalization. JAMA. 2003;290:1868–1874.

Costs are calculated using a resource utilization-based method of cost estimation. The following items, which are calculated using the Medicare claims data, are included in the total cost estimate:

- Accommodation costs, which are based on the number of general floor days and the number of intensive care unit days during the index admission. This information comes from the revenue center files.
- Operating room cost, based on the amount of time spent in the operating room (for patients who had a surgical procedure performed). This is determined using Part B claims.
- Emergency room visit fixed costs, based on post-discharge visits to the emergency room. This is determined using Part B claims.
- Costs of services provided, based on Relative Value Units (RVUs), determined using the Current Procedural Terminology codes on bills. This is determined using Part B claims.

In addition, any costs that occurred within 30 days of the index admission date, and all the costs associated with any readmissions that began within 30 days, are also included in the total cost calculation.

For peer review only - http://bmjopen.bmj.com/site/about/guidelines.xhtml

Drugs administered during the hospitalization are included in the cost calculation, but drugs outside the hospital are not. Costs of lawsuits are not available in the Medicare claims data.

Hospital medical errors that resulted in a longer length of stay or the use of additional hospital resources, such as the need to admit a patient to the intensive care unit, would be captured in the cost calculation.

For peer review only

Appendix Materials 3: Sleep Actigraphy Scoring

According to conventional standards, actigraphy data were classified in 1-minute epochs as wake, sleep, or missing. The first classification was performed by the algorithm of the device manufacturer (Actilife software, version 6.13.3, standard settings, Sadeh scoring algorithm). Off-wrist periods were identified by an algorithm developed by Pulsar Informatics Inc. (Philadelphia, PA), then visually checked, integrated with sleep log information provided by interns via the smartphone and, if necessary, corrected by co-investigators from Pulsar who were experienced with actigraphy scoring of large scale clinical trials. The classification into sleep, wake, and unknown followed the set of rules outlined in Appendix Figure 1. Pulsar's visual scoring of sleep was then independently checked by study investigators who are experts in sleep research. In an iterative process, any discrepancies were documented and then corrected by Pulsar, until agreement with the study investigators was reached. During this visual scoring process, both Pulsar and the study investigators were blinded to study arm (STD or FLEX). Likewise, PVT-B data were inspected by study sleep experts blinded to arm (Appendix Figure 2). PVT-B performance was classified into three categories as [1] adherent (i.e., PVT-B data reflected an effort to do the task correctly, and comments left by the subject did not suggest non-adherence), [2] possibly non-adherent (i.e., PVT-B data reflected a consistently poor effort to do the task correctly, but comments left by the subject did not suggest non-adherence), and [3] non-adherent (i.e., PVT-B data reflected a consistently poor effort to do the task correctly, and comments left by the subject did suggest non-adherence, e.g., performing the task while brushing teeth). Comments left by interns were inspected for distractions and non-fatigue related impairment and flagged accordingly. Comments that could have revealed the study arm were blacked out by Pulsar prior to classification by the study sleep experts.

For peer review only - http://bmjopen.bmj.com/site/about/guidelines.xhtml

Appendix Figure 1. Overview of Actigraphy and PVT Scoring and Review Process

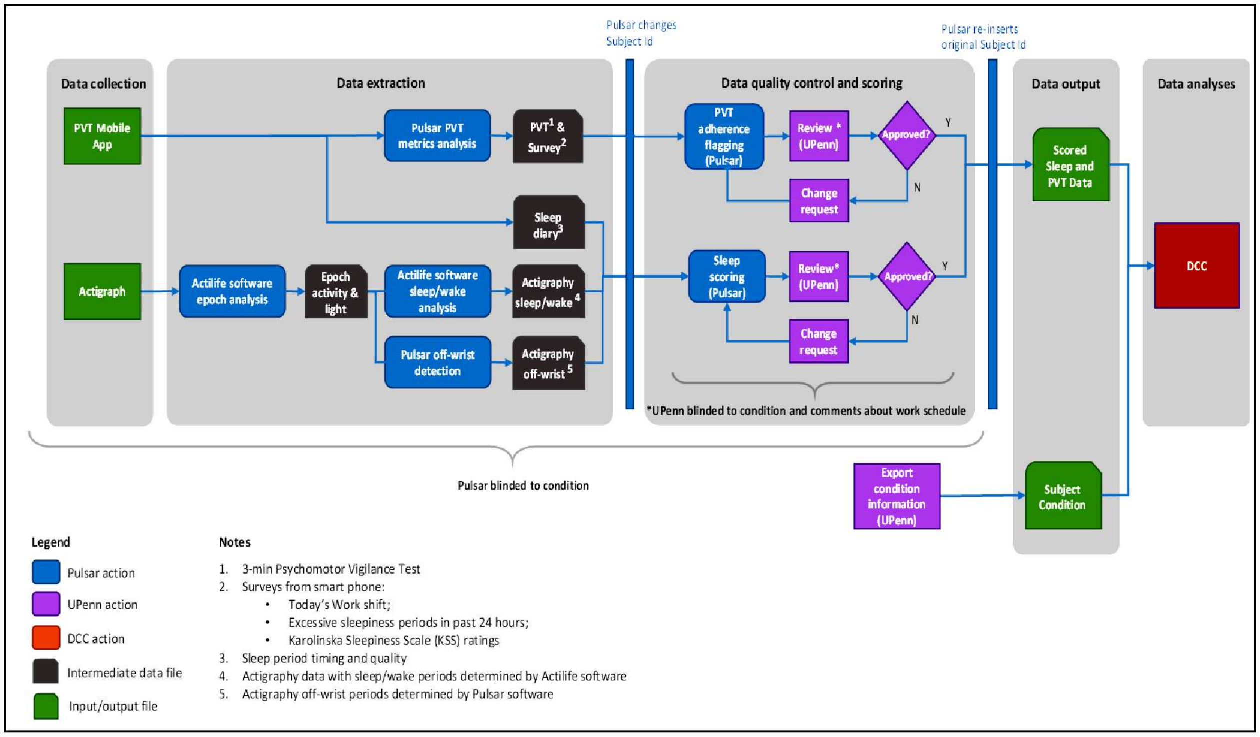

For peer review only - http://bmjopen.bmj.com/site/about/guidelines.xhtml

Appendix Figure 2: Sleep Scoring Matrix

	**Sleep Diary had sleep time entered (S)**	**Sleep Diary did not have sleep time entered (W)**
**Actigraphy Reviewed indicated sleep (S)**	Scored as Sleep (S) based on agreement of actigraphy and diary	Scored as Sleep (S) based on actigraphy
**Actigraphy Reviewed indicated wake (W)**	Scored as Wake (W) based on actigraphy	Scored as Wake (W) based on actigraphy
**Actigraphy Reviewed indicated missing or off-wrist (O)**	Scored as Sleep (S) based on diary	Scored as Unknown (U)

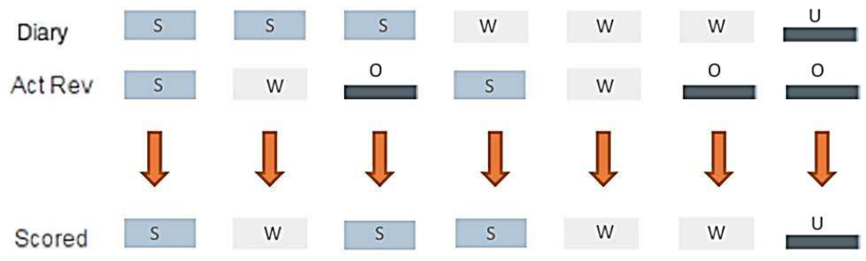

SPIRIT

STANDARD PROTOCOL ITEMS: RECOMMENDATIONS FOR INTERVENTIONAL TRIALS

SPIRIT 2013 Checklist: Recommended items to address in a clinical trial protocol and related documents*

Section/item	Item No	Description
**Administrative information**		
Title 1	1	Descriptive title identifying the study design, population, interventions, and, if applicable, trial acronym
Trial registration 2,4	2a	Trial identifier and registry name. If not yet registered, name of intended registry
	2b	All items from the World Health Organization Trial Registration Data Set
Protocol version	3	Date and version identifier NA
Funding 2	4	Sources and types of financial, material, and other support
Roles and responsibilities 1	5a	Names, affiliations, and roles of protocol contributors
	5b	Name and contact information for the trial sponsor
*	5c	Role of study sponsor and funders, if any, in study design; collection, management, analysis, and interpretation of data; writing of the report; and the decision to submit the report for publication, including whether they will have ultimate authority over any of these activities
*	5d	Composition, roles, and responsibilities of the coordinating centre, steering committee, endpoint adjudication committee, data management team, and other individuals or groups overseeing the trial, if applicable (see Item 21a for data monitoring committee)
**Introduction**		
Background and rationale 5-6	6a	Description of research question and justification for undertaking the trial, including summary of relevant studies (published and unpublished) examining benefits and harms for each intervention
	6b	Explanation for choice of comparators NA
Objectives 7	7	Specific objectives or hypotheses
Trial design 8,11	8	Description of trial design including type of trial (eg, parallel group, crossover, factorial, single group), allocation ratio, and framework (eg, superiority, equivalence, noninferiority, exploratory)

* In protocol, could be added to summary

For peer review only - http://bmjopen.bmj.com/site/about/guidelines.xhtml

Methods: Participants, interventions, and outcomes

Study setting	8	9	Description of study settings (eg, community clinic, academic hospital) and list of countries where data will be collected. Reference to where list of study sites can be obtained
Eligibility criteria	9-11	10	Inclusion and exclusion criteria for participants. If applicable, eligibility criteria for study centres and individuals who will perform the interventions (eg, surgeons, psychotherapists)
Interventions	– Table 2, 30	11a	Interventions for each group with sufficient detail to allow replication, including how and when they will be administered
	*	11b	Criteria for discontinuing or modifying allocated interventions for a given trial participant (eg, drug dose change in response to harms, participant request, or improving/worsening disease)
		11c	Strategies to improve adherence to intervention protocols, and any procedures for monitoring adherence (eg, drug tablet return, laboratory tests)
		11d	Relevant concomitant care and interventions that are permitted or prohibited during the trial
Outcomes	– Table 3, 31, 12-17	12	Primary, secondary, and other outcomes, including the specific measurement variable (eg, systolic blood pressure), analysis metric (eg, change from baseline, final value, time to event), method of aggregation (eg, median, proportion), and time point for each outcome. Explanation of the clinical relevance of chosen efficacy and harm outcomes is strongly recommended
Participant timeline	12, Figure 2	13	Time schedule of enrolment, interventions (including any run-ins and washouts), assessments, and visits for participants. A schematic diagram is highly recommended (see Figure)
Sample size	8-9	14	Estimated number of participants needed to achieve study objectives and how it was determined, including clinical and statistical assumptions supporting any sample size calculations
Recruitment	10	15	Strategies for achieving adequate participant enrolment to reach target sample size

Methods: Assignment of interventions (for controlled trials)

Allocation:

Sequence generation	11	16a	Method of generating the allocation sequence (eg, computer-generated random numbers), and list of any factors for stratification. To reduce predictability of a random sequence, details of any planned restriction (eg, blocking) should be provided in a separate document that is unavailable to those who enrol participants or assign interventions

Allocation concealment mechanism	16b	Mechanism of implementing the allocation sequence (eg, central telephone; sequentially numbered, opaque, sealed envelopes), describing any steps to conceal the sequence until interventions are assigned	
Implementation	16c*	Who will generate the allocation sequence, who will enrol participants, and who will assign participants to interventions	
Blinding (masking)	17a	Who will be blinded after assignment to interventions (eg, trial participants, care providers, outcome assessors, data analysts), and how	
	17b	If blinded, circumstances under which unblinding is permissible, and procedure for revealing a participant's allocated intervention during the trial	

Methods: Data collection, management, and analysis

Data collection methods	18a	Plans for assessment and collection of outcome, baseline, and other trial data, including any related processes to promote data quality (eg, duplicate measurements, training of assessors) and a description of study instruments (eg, questionnaires, laboratory tests) along with their reliability and validity, if known. Reference to where data collection forms can be found, if not in the protocol	10, 12-16
	18b	Plans to promote participant retention and complete follow-up, including list of any outcome data to be collected for participants who discontinue or deviate from intervention protocols	NA
Data management	19*	Plans for data entry, coding, security, and storage, including any related processes to promote data quality (eg, double data entry; range checks for data values). Reference to where details of data management procedures can be found, if not in the protocol	
Statistical methods	20a	Statistical methods for analysing primary and secondary outcomes. Reference to where other details of the statistical analysis plan can be found, if not in the protocol	17
	20b	Methods for any additional analyses (eg, subgroup and adjusted analyses)	
	20c*	Definition of analysis population relating to protocol non-adherence (eg, as randomised analysis), and any statistical methods to handle missing data (eg, multiple imputation)	

Methods: Monitoring

Data monitoring	21a	Composition of data monitoring committee (DMC); summary of its role and reporting structure; statement of whether it is independent from the sponsor and competing interests; and reference to where further details about its charter can be found, if not in the protocol. Alternatively, an explanation of why a DMC is not needed	6-7

	21b	Description of any interim analyses and stopping guidelines, including who will have access to these interim results and make the final decision to terminate the trial	
Harms	22	Plans for collecting, assessing, reporting, and managing solicited and spontaneously reported adverse events and other unintended effects of trial interventions or trial conduct	
Auditing	23	Frequency and procedures for auditing trial conduct, if any, and whether the process will be independent from investigators and the sponsor	
**Ethics and dissemination**			
Research ethics approval	24	Plans for seeking research ethics committee/institutional review board (REC/IRB) approval	11
Protocol amendments	25	Plans for communicating important protocol modifications (eg, changes to eligibility criteria, outcomes, analyses) to relevant parties (eg, investigators, REC/IRBs, trial participants, trial registries, journals, regulators)	NA
Consent or assent	26a	Who will obtain informed consent or assent from potential trial participants or authorised surrogates, and how (see Item 32)	
	26b	Additional consent provisions for collection and use of participant data and biological specimens in ancillary studies, if applicable	
Confidentiality	27	How personal information about potential and enrolled participants will be collected, shared, and maintained in order to protect confidentiality before, during, and after the trial	
Declaration of interests	28	Financial and other competing interests for principal investigators for the overall trial and each study site	
Access to data	29	Statement of who will have access to the final trial dataset, and disclosure of contractual agreements that limit such access for investigators	
Ancillary and post-trial care	30	Provisions, if any, for ancillary and post-trial care, and for compensation to those who suffer harm from trial participation	
Dissemination policy	31a	Plans for investigators and sponsor to communicate trial results to participants, healthcare professionals, the public, and other relevant groups (eg, via publication, reporting in results databases, or other data sharing arrangements), including any publication restrictions	
	31b	Authorship eligibility guidelines and any intended use of professional writers	
	31c	Plans, if any, for granting public access to the full protocol, participant-level dataset, and statistical code	6

For peer review only - http://bmjopen.bmj.com/site/about/guidelines.xhtml

**Appendices**		
Informed consent materials	32	Model consent form and other related documentation given to participants and authorised surrogates
Biological specimens	33	Plans for collection, laboratory evaluation, and storage of biological specimens for genetic or molecular analysis in the current trial and for future use in ancillary studies, if applicable

*It is strongly recommended that this checklist be read in conjunction with the SPIRIT 2013 Explanation & Elaboration for important clarification on the items. Amendments to the protocol should be tracked and dated. The SPIRIT checklist is copyrighted by the SPIRIT Group under the Creative Commons "Attribution-NonCommercial-NoDerivs 3.0 Unported" license.
